# Microwave-Assisted Kabachnik–Fields Reaction with Amino Alcohols as the Amine Component

**DOI:** 10.3390/molecules24081640

**Published:** 2019-04-25

**Authors:** Ádám Tajti, Enikő Szatmári, Franc Perdih, György Keglevich, Erika Bálint

**Affiliations:** 1 Department of Organic Chemistry and Technology, Budapest University of Technology and Economics, 1521 Budapest, Hungary; tajti.adam@mail.bme.hu (Á.T.); szatmari.eniko93@gmail.com (E.S.); gkeglevich@mail.bme.hu (G.K.); 2 Faculty of Chemistry and Chemical Technology, University of Ljubljana, SI-1000 Ljubljana, Slovenia; franc.perdih@fkkt.uni-lj.si

**Keywords:** α-aminophosphonate derivatives, Kabachnik–Fields reaction, amino alcohols, microwave, X-ray diffraction

## Abstract

In this paper, the microwave (MW)-assisted catalyst-free and mostly solvent-free Kabachnik–Fields reaction of amino alcohols, paraformaldehyde, and various >P(O)H reagents (dialkyl phosphites, ethyl phenyl-*H*-phosphinate, and secondary phosphine oxides) is reported. The synthesis of *N*-2-hydroxyethyl-α-aminophosphonate derivatives was optimized in respect of the temperature, the reaction time, and the molar ratio of the starting materials. A few by-products were also identified. *N*,*N*-Bis(phosphinoylmethyl)amines containing a hydroxyethyl group were also prepared by the double Kabachnik–Fields reaction of ethanolamine with an excess of paraformaldehyde and secondary phosphine oxides. The crystal structure of a 2-hydroxyethyl-α-aminophosphine oxide and a bis(phosphinoylmethyl)ethanolamine was studied by X-ray analysis.

## 1. Introduction

α-Aminophosphonate derivatives are among the most important organophosphorus compounds [1]. Due to the P–C–N moiety in the α-aminophosphonic skeleton, these compounds can be considered as the P-analogues of natural α-amino acids, which may mean a potential biological activity [2].

α-Aminophosphonates and α-aminophosphine oxides containing a reactive group may show special properties as compared to regular derivatives. In case of the closely related α-aminophosphines [3], a -COOH function on the molecule made possible further transformations and ensured a linkage for a polymer support [4,5].

Several reactive end groups can be easily built on the α-aminophosphonate skeleton. A possible reactive function is the hydroxyl group, which can be alkylated, acylated, or even phosphorylated [6]. Another option is the carboxylic function, which may also mean a possibility for further functionalizations [7]. α-Aminophosphonates containing a carboxylic group may be synthesized starting from amino acids [8,9].

The conventional preparations of α-aminophosphonates and related derivatives are the Kabachnik–Fields (phospha-Mannich) reaction, in which an amine, an oxo-compound, and a >P(O)H derivative react with each other [10,11], and the aza-Pudovik reaction, in which a >P(O)H reagent is added on the C=N double bond of an imine [12].

Over the seven decades from its discovery, several exotic catalysts and/or solvents have been tried out in the Kabachnik–Fields reaction [13,14]. However, in most cases, catalyst-free and often solvent-free approaches could be performed applying the microwave (MW) technique [9,13,15,16,17,18].

The Kabachnik–Fields condensation of amino alcohols is a less studied area. The condensation of ethanolamine was investigated with oxo compounds (e.g., paraformaldehyde [19], benzaldehyde [20], or acetone [19]) and dialkyl phosphites. A MW-assisted, Al_2_O_3_-catalyzed variation was also reported; however, the reactions were carried out in a kitchen MW oven [21]. Using a non-professional MW device, the precise measurement of the reaction parameters is practically impossible [22]. The phospha-Mannich reaction of propanolamine was performed in toluene [23]. In two instances, *N*-alkylamino alcohols served as the amine component, and the reactions were carried out in a solvent for a long reaction time [24,25]. A few special amino alcohols, such as 2-amino-2-methylpropanole [26] or (*R*)-2-phenylglycinol [27] were also tried out in the condensation.

The double Kabachnik–Fields reaction of an amino alcohol was mentioned in only one example [28]. The reaction was carried out for 12 h in THF.

The utilization of secondary phosphine oxides as the P-component was reported in one instance [29]. In this example, the conventional heating and the MW technique were compared, and the latter was found to be more efficient. However, it should be noted that the MW-assisted reactions were performed in a kitchen MW oven.

In this paper, we introduce the synthesis of 2-hydroxyethyl-α-aminophosphonates and 2-hydroxyethyl-α-aminophoshine oxides by the MW-assisted Kabachnik–Fields condensation of amino alcohols, paraformaldehyde, and >P(O)H reagents, such as dialkyl phosphites, ethyl phenyl-*H*-phosphinate, and secondary phosphine oxides. We also aimed at developing a green, catalyst-free and mostly solvent-free synthesis, as well as at the preparation of new derivatives.

## 2. Results and Discussion

**Scheme 1:**
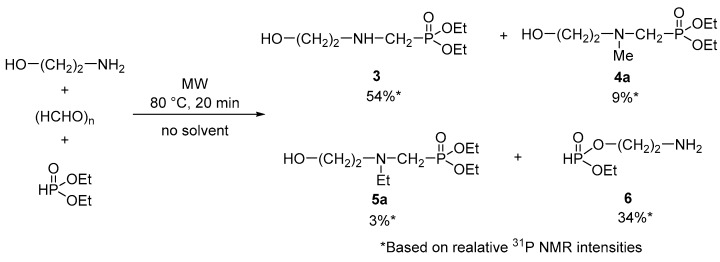
Condensation of ethanolamine, paraformaldehyde, and diethyl phosphite.

In the first step, the Kabachnik–Fields reaction of ethanolamine, paraformaldehyde, and diethyl phosphite was studied at 80 °C for 20 min in the absence of a solvent and a catalyst (Scheme 1). Although full conversion was achieved, beside the expected α-aminophosphonate (**3**), the mixture comprised 9% of the *N*-methylated-α-aminophosphonate (**4a**) and 3% of the *N*-ethylated-α-aminophosphonate (**5a**), as well as 34% of the 2-aminoethyl ethyl phosphite (**6**) based on ^31^P NMR.

Formation of the *N*-methyl-α-aminophosphonate (**4a**) may be explained with the methylation by the paraformaldehyde, which side reaction was also observed in similar Kabachnik–Fields reactions of ethyl octyl phosphite [18] or alkyl phenyl-*H*-phosphinates [16]. The *N*-ethylated by-product (**5a**) may have formed in the alkylation of compound **3** by diethyl phosphite, which is also a known side reaction during similar transformations [30]. The 2-aminoethyl ethyl phosphite (**6**), which was present in the highest proportion of 34%, is probably the product of the alcoholysis of diethyl phosphite by ethanolamine [31,32].

In the next series of experiments, the condensation of *N*-methylethanolamine, paraformaldehyde, and diethyl phosphite was investigated (Table 1). Carrying out the reaction at 60 °C for 20 min, the conversion was 85%, and the mixture comprised 96% of the desired *N*-hydroxyethyl-*N*-methyl-α-aminophosphonate (**4a**) and 4% of *H*-phosphonate **7** formed in the alcoholysis of diethyl phosphite by *N*-methylethanolamine (Table 1, Entry 1). In accordance to our previous experiences [32], the *N*-methyl-ethanolamine was much less active in the alcoholysis than the ethanolamine. Repeating the experiment at 80 °C, the reaction reached a full conversion, and the ratio of products **4a** and **7** was 95:5, respectively (Table 1, Entry 2). The comparative thermal experiment under similar conditions was less selective, since 81% of the desired product (**4a**) and 19% of compound **7** were present in the mixture (Table 1, Entry 3). At 100 °C for 10 min, the ratio of the by-product (**7**) increased (Table 1, Entry 4). Based on the results obtained, the temperature of 80 °C and the reaction time of 20 min were found to be the optimum conditions (Table 1, Entry 2).

Next, the condensation of *N*-alkylethanolamines, paraformaldehyde, and dialkyl phosphites or ethyl phenyl-*H*-phosphinate was studied under the optimized conditions (80 °C and 20 min). The reactions were complete in all the cases. Using *N*-methylethanolamine, the diethyl (*N*-2-hydroxyethyl)(*N*-methyl)aminomethylphosphonate (**4a**) was isolated in a yield of 78% (Table 2, Entry 1). Changing for dibutyl phosphite, the desired product (**4b**) was obtained in a yield of 87% after column chromatography (Table 2, Entry 2). The ethyl phenyl-*H*-phosphinate was also tried out as the phosphorus reagent; however, the α-aminophosphinate (**4c**) could be prepared in a slightly lower yield (67%) as compared to the α-aminophosphonates (Table 2, Entry 3). Carrying out the experiments starting from *N*-ethylethanolamine, the condensations took place similarly (Table 2, Entries 4-6). The diethyl (**5a**) and the dibutyl 2-ethyl-2-hydroxyethyl-α-aminophoshhonate (**5b**) were prepared in yields of 72% and 79%, respectively; while using ethyl phenyl *H*-phosphinate as the P-reagent, product **5c** was isolated in a yield of 64%.

The transformations were also performed using secondary phosphine oxides (Table 3 and Table 4). In these reactions, some acetonitrile had to be used to overcome the heterogeneity. First, the condensation of ethanolamine, paraformaldehyde, and diphenylphosphine oxide was investigated (Table 3). In these experiments, only the desired product (**8a**) was formed, and no by-product was observed. Carrying out the reaction at 80 °C for 20 min, the α-aminophosphine oxide (**8a**) was obtained in a conversion of 58% (Table 3, Entry 1). Prolonging the irradiation to 30 min, a significantly higher conversion (86%) could be reached (Table 3, Entry 2). Any further increase in the reaction time did not cause a significant change in the conversion (Table 3, Entry 3). Performing the three-component reaction at 100 °C, the conversion was already 92% after 20 min; using a reaction time of 30 min, the condensation was complete (Table 3, entries 4 and 5).

The condensation of ethanolamine with paraformaldehyde and other secondary phosphine oxides was also carried out (Table 4, entries 1–3). Using the optimized conditions (100 °C, 30 min), the reactions were complete in all the cases. Applying diphenylphosphine oxide as the P-component, the corresponding 2-hydroxyethyl-α-aminophosphine oxide (**8a**) was obtained in a yield of 96% after column chromatography (Table 4, Entry 1). Changing for bis(*p*-tolyl)phosphine oxide or bis(3,5-dimethylphenyl)phosphine oxide, the reactions took place similarly, and the desired products (**8b** or **8c**) were isolated in yields of 89% and 95%, respectively (Table 4, entries 2 and 3). The condensation was also extended for using *N*-alkylethanolamines (*N*-methyl-, *N*-ethyl-, or *N*-benzylethanolamine), and the corresponding α-aminophosphine oxide derivatives (**9**–**11a**–**c**) were obtained in high yields (88–96%) (Table 4, entries 4–12).

In the next round, the double Kabachnik–Fields condensation of ethanolamine with an excess of paraformaldehyde and secondary phosphine oxides was studied (Table 5). As the first experiment, the ethanolamine was reacted with two equivalents of the paraformaldehyde and the diphenylphosphine oxide at 100 °C for 1 h (Table 5, Entry 1). It was found that the mono α-aminophosphine oxide (**8a**) was the main product (75%), while the desired *N*,*N*-bis(diphenylphosphinoylmethyl)ethanolamine (**12a**) was present in a proportion of only 25%. Increasing the temperature to 120 °C, the ratio of product **12a** increased to 34% (Table 5, Entry 2). Prolonging the reaction time to 1.5 h, the composition did not change significantly (Table 5, Entry 3). As the next step, the effect of the molar ratio of starting materials was investigated (Table 5, entries 4–6). By using 2.5 equivalents of the diphenylphosphine oxide, the ratio of the mono (**8a**) and the bis product (**12a**) remained almost unchanged (Table 5, Entry 4). When both reagents (the paraformaldehyde and the diphenylphosphine oxide) were used in 2.5 equivalents quantity, the proportion of product **12a** increased significantly (Table 5, Entry 5). Further increase in the molar ratios to three equivalents allowed a full transformation toward the *N*,*N*-bis(diphenylphosphinoylmethyl)ethanolamine (**12a**), which was obtained in a yield of 95% after purification (Table 5, Entry 6). The double Kabachnik–Fields reaction was also carried out starting from bis(*p*-tolyl)phosphine oxide or bis(3,5-dimethylphenyl)phosphine oxide using the optimized conditions (Table 5, entries 7 and 8). The *p*-tolyl-substituted product (**12b**) was synthesized in a yield of 93%, while product **12c** could be isolated in a yield of 91%.

**Figure 1:**
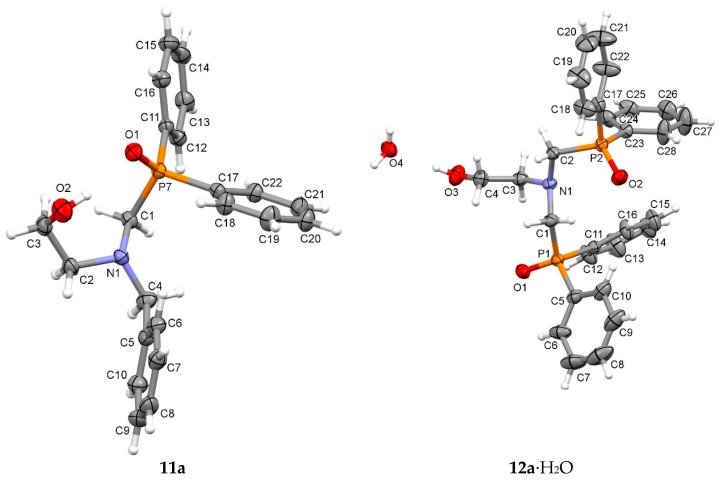
X-ray structures and atom numbering of compounds **11a** and **12a**∙H_2_O. Probability ellipsoids are drawn at the 30% level.

In addition to the spectroscopic analysis, we have determined the crystal structure of **11a** and **12a**∙H_2_O by single-crystal XRD analysis (Figure 1). In the structure of **11a**, an intramolecular O–H···O=P hydrogen bond is present between the hydroxy group as the donor and the P=O group as the acceptor. Moreover, intermolecular C–H···O=P interactions enable the formation of hydrogen-bonded chains, which are connected into layers via C–H∙∙∙π interactions (Figure S1, Table S1). In the structure of **12a**∙H_2_O, an O–H···O hydrogen bond is present between the hydroxy group (donor) and the water hydrate molecule (acceptor). The H_2_O forms two more O–H···O=P hydrogen bonds with P=O groups of two adjacent molecules, resulting in the formation of wavy layers enhanced by C–H∙∙∙O interactions. These layers are connected into a supramolecular structure via additional C–H∙∙∙O interactions (Figure S2, Table S1).

## 3. Materials and Methods

### 3.1. General

The reactions were carried out in a 300-W CEM Discover focused microwave reactor (CEM Microwave Technology Ltd., Buckingham, UK) equipped with a pressure controller using 10–50 W irradiation under isothermal conditions.

HPLC-MS measurements were performed with an Agilent 1200 liquid chromatography system coupled with a 6130 quadrupole mass spectrometer equipped with an ESI ion source (Agilent Technologies, Palo Alto, CA, USA). Analysis was performed at 40 °C on a Gemini C18 column (150 mm × 4.6 mm, 3 µm; Phenomenex, Torrance, CA, USA) with a mobile phase flow rate of 0.6 mL/min. The composition of eluent A was 0.1% (NH_4_)(HCOO) in water; eluent B was 0.1% (NH_4_)(HCOO) and 8% water in acetonitrile. 0–3 min. 5% B, 3–13 min. gradient, 13–20 min. 95% B. The injection volume was 5 µL. The chromatographic profile was registered at 254 nm. The MSD operating parameters were as follows: positive ionization mode, scan spectra from *m/z* 100 to 1000, drying gas temperature 300 °C, nitrogen flow rate 12 L/min, nebulizer pressure 60 psi, and capillary voltage 4000 V.

High-resolution mass spectrometric measurements were performed using a Q-TOF Premier mass spectrometer in positive electrospray mode.

The ^31^P, ^1^H, ^13^C, and NMR spectra were taken in CDCl_3_ solution on a Bruker AV-300 spectrometer (Bruker AXS GmBH, Karlsruhe, Germany) operating at 121.5 MHz, 75.5 MHz, and 300 MHz, respectively. Chemical shifts are downfield relative to 85% H_3_PO_4_ and TMS.

### 3.2. General Procedure for the Synthesis of the 2-Hydroxyethyl-α-aminophosphonates and -α-Aminophosphinates

The mixture of 1.0 mmol of amino alcohol [ethanolamine (0.06 mL), *N*-methylethanolamine (0.08 mL), or *N*-ethylethanolamine (0.10 mL)], 1.0 mmol of paraformaldehyde (0.03 g), and 1.0 mmol of >P(O)H reagent [diethyl phosphite (0.13 mL), dibutyl phosphite (0.20 mL), or ethyl phenyl-*H*-phosphinate (0.17 g)] was irradiated in a sealed tube at 80 °C for 20 min in a CEM Discover Microwave reactor equipped with a pressure controller. The crude product was purified by flash column chromatography using silica gel and dichloromethane–methanol 9:1 as the eluent. Thus, the following products were prepared:

*Diethyl (N-2-hydroxyethyl)(N-methyl)aminomethylphosphonate* (**4a**): Yield: 78% (0.18 g), yellow oil; ^31^P NMR (CDCl_3_) δ: 26.6; ^13^C NMR (CDCl_3_) δ: 16.5 (d, ^3^*J*_CP_ = 5.9, OCH_2_*C*H_3_), 44.8 (d, ^3^*J*_CP_ = 6.5, NCH_3_), 52.5 (d, ^1^*J*_CP_ = 165.6, CH_2_P), 59.3 (HOCH_2_), 60.9 (d, ^3^*J*_CP_ = 10.9, CH_2_*C*H_2_N), 62.2 (d, ^2^*J*_CP_ = 7.1, O*C*H_2_CH_3_); ^1^H NMR (CDCl_3_) δ: 1.34 (t, *J*_HH_ = 7.1, 6H, OCH_2_C*H*_3_), 2.48 (s, 3H, NCH_3_), 2.69 (t, *J*_HH_ = 5.2, 2H, CH_2_N), 2.87 (d, *J*_HP_ = 10.6, 2H, CH_2_P), 3.62 (t, *J*_HH_ = 5.2, 2H, HOC*H*_2_), 4.06–4.25 (m, 4H, OC*H*_2_CH_3_); [M + H]^+^_found_ = 226.1201, C_8_H_21_NO_4_P requires 226.1208.

*Dibutyl (N-2-hydroxyethyl)(N-methyl)aminomethylphosphonate* (**4b**): Yield: 84% (0.24 g), yellow oil; ^31^P NMR (CDCl_3_) δ: 25.7; ^13^C NMR (CDCl_3_) δ: 13.6 (CH_2_*C*H_3_), 18.7 (*C*H_2_CH_3_), 32.6 (d, ^3^*J*_CP_ = 5.8, *C*H_2_CH_2_CH_3_), 44.7 (d, ^3^*J*_CP_ = 6.5, NCH_3_), 52.3 (d, ^1^*J*_CP_ = 165.2, CH_2_P), 59.2 (HOCH_2_), 60.9 (d, ^3^*J*_CP_ = 10.5, CH_2_*C*H_2_N) 65.9 (d, ^2^*J*_CP_ = 7.1, O*C*H_2_CH_2_CH_2_); ^1^H NMR (CDCl_3_) δ: 0.94 (t, *J*_HH_ = 7.4, 6H, CH_2_C*H_3_*), 1.33-1.47 (m, 4H, C*H*_2_CH_3_), 1.62–1.72 (m, 4H, C*H*_2_CH_2_CH_3_), 2.48 (s, 3H, NCH_3_), 2.68 (t, *J*_HH_ = 5.1, 2H, CH_2_N), 2.87 (d, *J*_HP_ = 10.7, 2H, CH_2_P), 3.61 (t, *J*_HH_ = 5.1, 2H, HOC*H*_2_), 4.02–4.14 (m, 4H, OC*H*_2_CH_2_CH_2_); [M + H]^+^_found_ = 282.1814, C_12_H_29_NO_4_P requires 282.1834.

*Ethyl ([N-2-hydroxyethyl][N-methyl]aminomethyl)(phenyl)phosphinate* (**4c**): Yield: 67% (0.17 g), yellow oil; ^31^P NMR (CDCl_3_) δ: 39.5; ^13^C NMR (CDCl_3_) δ: 16.6 (d, ^3^*J*_CP_ = 6.1, OCH_2_*C*H_3_), 45.1 (d, ^3^*J*_CP_ = 5.3, NCH_3_), 55.9 (d, ^1^*J*_CP_ = 122.5, CH_2_P), 59.3 (HOCH_2_) 61.1 (d, ^2^*J*_CP_ = 6.8, O*C*H_2_CH_2_), 61.5 (d, ^3^*J*_CP_ = 10.2, *C*H_2_N), 128.8 (d, ^3^*J*_CP_ = 12.3, C_3_), 130.2 (d, ^1^*J*_CP_ = 122.3, C_1_), 132.0 (d, ^2^*J*_CP_ = 9.6, C_2_), 132.6 (d, *J*_CP_ = 2.7, C_4_); ^1^H NMR (CDCl_3_) δ: 1.32 (t, *J*_HH_ = 7.0, 3H, OCH_2_C*H_3_*), 2.93 (s, 3H, NCH_3_), 2.56-2.69 (m, 2H, CH_2_N), 2.95-3.06 (m, 2H, CH_2_P), 3.43–3.58 (m, 2H, HOC*H*_2_), 3.89–3.95 (m, 1H, CH_A_, OC*H*_2_CH_3_), 4.09–4.19 (m, 1H, CH_B_, OC*H*_2_CH_3_), 7.47–7.54 (m, 2H, C_2_H), 7.55–7.61 (m, 1H, C_4_H), 7.78–7.86 (m, 2H, C_3_H); [M + H]^+^_found_ = 258.1247, C_12_H_21_NO_3_P requires 258.1259.

*Diethyl (N-ethyl)(N-2-hydroxyethyl)aminomethylphosphonate* (**5a**): Yield: 72% (0.17 g), pale yellow oil; ^31^P NMR (CDCl_3_) δ: 26.4; ^13^C NMR (CDCl_3_) δ: 11.4 (NCH_2_*C*H_3_), 16.5 (d, ^3^*J*_CP_ = 5.7, OCH_2_*C*H_3_), 49.1 (d, ^1^*J*_CP_ = 168.1, CH_2_P), 50.5 (d, ^3^*J*_CP_ = 7.7, N*C*H_2_CH_3_), 57.4 (d, ^3^*J*_CP_ = 8.2, CH_2_*C*H_2_N), 59.8 (HOCH_2_), 62.2 (d, ^2^*J*_CP_ = 7.0, O*C*H_2_CH_3_); ^1^H NMR (CDCl_3_) δ: 1.05 (t, *J*_HH_ = 7.0, 3H, NCH_2_C*H*_3_), 1.34 (t, *J*_HH_ = 7.0, 6H, OCH_2_C*H*_3_), 2.69–2.81 (m, 4H, NC*H*_2_CH_3_, CH_2_C*H_2_*N), 2.91 (d, *J*_HP_ = 10.2, 2H, CH_2_P), 3.58–3.63 (m, 2H, HOC*H*_2_), 4.11–4.21 (m, 4H, OCH_2_CH_3_); [M + H]^+^_found_ = 240.1353, C_9_H_23_NO_4_P requires 240.1365.

*Dibutyl (N-ethyl)(N-2-hydroxyethyl)aminomethylphosphonate* (**5b**): Yield: 79% (0.23 g), pale yellow oil; ^31^P NMR (CDCl_3_) δ: 26.5; ^13^C NMR (CDCl_3_) δ: 11.5 (NCH_2_*C*H_3_), 13.6 (CH_2_CH_2_*C*H_3_), 18.7 (CH_2_*C*H_2_CH_3_), 32.7 (d, ^3^*J*_CP_ = 5.8, OCH_2_*C*H_2_CH_2_), 49.0 (d, ^1^*J*_CP_ = 167.5, CH_2_P), 50.4 (d, ^3^*J*_CP_ = 7.6, N*C*H_2_CH_3_), 57.4 (d, ^3^*J*_CP_ = 8.2, CH_2_*C*H_2_N), 59.7 (HO*C*H_2_), 66.0 (d, ^2^*J*_CP_ = 7.3, O*C*H_2_CH_3_); ^1^H NMR (CDCl_3_) δ: 0.94 (t, *J*_HH_ = 7.4, 6H, CH_2_CH_2_C*H*_3_), 1.05 (t, *J*_HH_ = 7.1, 3H, NCH_2_C*H*_3_), 1.32–1.49 (m, 4H, CH_2_C*H*_2_CH_3_), 1.58–1.73 (m, 4H, C*H*_2_CH_2_CH_3_), 2.66–2.82 (m, 4H, CH_2_C*H*_2_N, NC*H_2_*CH_3_), 2.91 (d, *J*_HH_ = 10.3, 2H, CH_2_P), 3.60 (t, *J*_HH_ = 5.1, 2H, HOC*H*_2_) 3.98–4.16 (m, 4H, OC*H*_2_CH_3_); [M + H]^+^_found_ = 296.1976, C_13_H_31_NO_4_P requires 296.1991.

*Ethyl ([N-ethyl][N-2-hydroxyethyl]aminomethyl)(phenyl)phosphinate* (**5c**): Yield: 64% (0.17 g), pale yellow oil; ^31^P NMR (CDCl_3_) δ: 40.1; ^13^C NMR (CDCl_3_) δ: 11.2 (NCH_2_*C*H_3_), 16.5 (d, ^3^*J*_CP_ = 6.1, OCH_2_*C*H_3_), 50.7 (d, ^3^*J*_CP_ = 6.2, N*C*H_2_CH_3_), 52.8 (d, ^1^*J*_CP_ = 124.0, CH_2_P), 57.7 (d, ^3^*J*_CP_ = 7.8, CH_2_*C*H_2_N), 59.9 (HOCH_2_), 61.1 (d, ^2^*J*_CP_ = 7.0, O*C*H_2_CH_3_), 128.7 (d, ^3^*J*_CP_ = 12.2, C_3_), 130.1 (d, ^1^*J*_CP_ = 121.8, C_1_), 132.0 (d, ^2^*J*_CP_ = 9.6, C_2_) 132.5 (d, *J*_CP_ = 2.8, C_4_); ^1^H NMR (CDCl_3_) δ: 0.86 (t, *J*_HH_ = 7.1, 3H, NCH_2_C*H*_3_), 1.32 (t, *J*_HH_ = 7.1, 3H, OCH_2_C*H*_3_), 2.51–2.62 (m, 2H, CH_2_C*H*_2_N), 2.62-2.74 (m, 2H, NC*H_2_*CH_3_), 2.95–3.10 (m, 2H, CH_2_P), 3.48–3.59 (m, 2H, HOC*H*_2_) 3.88–3.98 (m, 1H, CH_A_, OC*H*_2_CH_3_) 4.10–4.19 (m, 1H, CH_B_, OC*H*_2_CH_3_) 7.47–7.53 (m, 2H, C_2_H), 7.55–7.61 (m, 1H, C_4_H), 7.78–7.84 (m, 2H, C_3_H); [M + H]^+^_found_ = 272.1405, C_13_H_23_NO_3_P requires 272.1416.

### 3.3. General Procedure for the Synthesis of the 2-Hydroxyethyl-α-aminophosphine oxides

The mixture of 1.0 mmol of amino alcohol [ethanolamine (0.06 mL), *N*-methylethanolamine (0.08 mL), *N*-ethylethanolamine (0.10 mL), or *N*-benzylethanolamine (0.14 mL)], 1.0 mmol of paraformaldehyde (0.03 g) and 1.0 mmol of secondary phosphine oxide [diphenylphosphine oxide (0.20 g), bis(*p*-tolyl)phosphine oxide (0.23 g), or bis(3,5-dimethylphenyl)phosphine oxide (0.26 g)] in 2 mL of acetonitrile was irradiated in a sealed tube at 100 °C for 30 min in a CEM Discover Microwave reactor equipped with a pressure controller. The crude product was purified by flash column chromatography using silica gel and dichloromethane–methanol 9:1 as the eluent. Thus, the following products were prepared:

*(2-Hydroxyethylaminomethyl)diphenylphosphine oxide* (**8a**): Yield: 96% (0.27 g), white crystal; Mp: 84–85 °C; ^31^P NMR (CDCl_3_) δ: 30.1; ^13^C NMR (CDCl_3_) δ: 48.6 (d, ^1^*J*_CP_ = 80.2, CH_2_P), 53.0 (d, ^3^*J*_CP_ = 11.6, CH_2_N), 60.6 (HOCH_2_), 128.7 (d, ^3^*J*_CP_ = 11.6, C_3_), 131.1 (d, ^2^*J*_CP_ = 9.3, C_2_), 131.6 (d, ^1^*J*_CP_ = 97.8, C_1_), 132.1 (d, *J*_CP_ = 2.8, C_4_); ^1^H NMR (CDCl_3_) δ: 2.76 (brs, 1H, NH), 2.84 (t, *J*_HH_ = 5.1, 2H, CH_2_N), 3.53 (d, *J*_HP_ = 6.9, 2H, CH_2_P), 3.61 (t, *J*_HH_ = 5.0, 2H, OCH_2_), 7.38–7.60 (m, 6H, C_2_H, C_4_H), 7.68–7.86 (m, 4H, C_3_H); [M + H]^+^_found_ = 276.1146, C_15_H_19_NO_2_P requires 276.1153.

*(2-Hydroxyethylaminomethyl)bis(p-tolyl)phosphine oxide* (**8b**): Yield: 89% (0.27 g), pale yellow viscous oil; ^31^P NMR (CDCl_3_) δ: 30.8; ^13^C NMR (CDCl_3_) δ: 21.6 (C_4_*C*H_3_), 48.6 (d, ^1^*J*_CP_ = 79.7, CH_2_P), 53.0 (d, ^1^*J*_CP_ = 11.7, CH_2_N), 60.5 (HOCH_2_), 128.2 (d, ^1^*J*_CP_ = 100.6, C_1_), 129.5 (d, ^3^*J*_CP_ = 11.9, C_3_), 131.1 (d, ^2^*J*_CP_ = 9.6, C_2_), 142.6 (d, *J*_CP_ = 2.7, C_4_); ^1^H NMR (CDCl_3_) δ: 2.30 (s, 6H, C_4_CH_3_), 2.75 (t, *J*_HH_ = 5.1, 2H, CH_2_N), 2.86 (brs, 1H, NH), 3.40 (d, *J*_HP_ = 6.8, 2H, CH_2_P), 3.51 (t, *J*_HH_ = 5.0, 2H, OCH_2_), 7.12–7.26 (m, 4H, C_2_H), 7.46–7.64 (m, 2H, C_3_H); [M + H]^+^_found_ = 304.1456, C_17_H_23_NO_2_P requires 304.1466.

*(2-Hydroxyethylaminomethyl)bis(3,5-dimethylphenyl)phosphine oxide* (**8c**): Yield: 95% (0.31 g), pale yellow viscous oil; ^31^P NMR (CDCl_3_) δ: 30.5; ^13^C NMR (CDCl_3_) δ: 21.4 (C_3_*C*H_3_), 48.3 (d, ^1^*J*_CP_ = 78.1, CH_2_P), 52.8 (d, ^3^*J*_CP_ = 10.5, CH_2_N), 60.5 (HOCH_2_), 128.5 (d, ^2^*J*_CP_ = 9.2, C_2_), 131.4 (d, ^1^*J*_CP_ = 97.0, C_1_), 133.9 (d, *J*_CP_ = 2.8, C_4_), 138.5 (d, ^3^*J*_CP_ = 12.3, C_3_); ^1^H NMR (CDCl_3_) δ: 2.34 (s, 12H, C_3_CH_3_), 2.52 (brs, 1H, NH), 2.87 (t, *J*_HH_ = 5.0, 2H, CH_2_N), 3.49 (d, *J*_HP_ = 6.3, 2H, CH_2_P), 3.60 (t, *J*_HH_ = 4.9, 2H, OCH_2_), 7.16 (s, 2H, C_4_H), 7.36 (d, *J*_HH_ = 11.7, 4H, C_2_H); [M + H]^+^_found_ = 332.1771, C_19_H_27_NO_2_P requires 332.1779.

*[(N-2-Hydroxyethyl)(N-methyl)aminomethyl]diphenylphosphine oxide* (**9a**): Yield: 95% (0.27 g), pale yellow viscous oil; ^31^P NMR (CDCl_3_) δ: 28.5; ^13^C NMR (CDCl_3_) δ: 45.6 (d, ^3^*J*_CP_ = 5.4, NCH_3_), 56.9 (d, ^1^*J*_CP_ = 89.0, CH_2_P), 59.5 (HOCH_2_), 61.9 (d, ^3^*J*_CP_ = 8.1 CH_2_N), 128.7 (d, ^3^*J*_CP_ = 11.4, C_3_), 131.1 (d, ^2^*J*_CP_ = 9.0, C_2_), 131.7 (d, ^1^*J*_CP_ = 97.0, C_1_), 132.0 (d, *J*_CP_ = 2.7, C_4_); ^1^H NMR (CDCl_3_) δ: 2.35 (s, 3H, NCH_3_), 2.71 (t, *J*_HH_ = 5.0, 2H, CH_2_N), 3.38 (d, *J*_HP_ = 4.6, 2H, CH_2_P), 3.59 (t, *J*_HH_ = 5.0, 2H, OCH_2_), 7.45–7.52 (m, 4H, C_2_H), 7.52–7.57 (m, 2H, C_4_H), 7.75–7.83 (m, 4H, C_3_H); [M + H]^+^_found_ = 290.1300, C_16_H_21_NO_2_P requires 290.1310.

*[(N-2-Hydroxyethyl)(N-methyl)aminomethyl]bis(p-tolyl)phosphine oxide* (**9b**): Yield: 96% (0.30 g), pale yellow viscous oil; ^31^P NMR (CDCl_3_) δ: 29.0; ^13^C NMR (CDCl_3_) δ: 21.6 (*C_4_CH_3_*), 45.6 (d, ^3^*J*_CP_ = 5.5, NCH_3_), 57.1 (d, ^1^*J*_CP_ = 89.1, CH_2_P), 59.5 (HOCH_2_), 61.9 (d, ^3^*J*_CP_ = 8.0, CH_2_N), 128.6 (d, ^1^*J*_CP_ = 99.4, C_1_), 129.4 (d, ^3^*J*_CP_ = 11.8, C_3_), 131.1 (d, ^2^*J*_CP_ = 9.3, C_2_), 142.5 (d, *J*_CP_ = 2.8, C_4_); ^1^H NMR (CDCl_3_) δ: 2.35 (s, 3H, NCH_3_), 2.40 (s, 6H, C_4_CH_3_), 2.70 (t, *J*_HH_ = 5.0, 2H, CH_2_N), 3.33 (d, *J*_HP_ = 4.8, 2H, CH_2_P), 3.58 (t, *J*_HH_ = 5.0, 2H, OCH_2_), 7.20–7.37 (m, 4H, C_4_H), 7.58–7.74 (m, 4H, C_3_H); [M + H]^+^_found_ = 318.1620, C_18_H_25_NO_2_P requires 318.1623.

*(N-2-Hydroxyethyl)(N-methyl)aminomethyl]bis(3,5-dimethylphenyl)phosphine oxide* (**9c**): Yield: 93% (0.32 g), pale yellow viscous oil; ^31^P NMR (CDCl_3_) δ: 28.9; ^13^C NMR (CDCl_3_) δ: 21.3 (C_3_*C*H_3_), 45.7 (d, ^3^*J*_CP_ = 5.0, NCH_3_), 56.8 (d, ^1^*J*_CP_ = 88.3, CH_2_P), 59.4 (OCH_2_), 62.0 (d, ^3^*J*_CP_ = 8.3 CH_2_N), 128.6 (d, ^2^*J*_CP_ = 9.0, C_2_), 131.7 (d, ^1^*J*_CP_ = 96.2, C_1_), 133.7 (d, *J*_CP_ = 2.9, C_4_), 138.4 (d, ^3^*J*_CP_ = 12.0, C_3_); ^1^H NMR (CDCl_3_) δ: 2.35 (s, 12H, C_3_CH_3_), 2.38 (s, 3H, NCH_3_), 2.70 (t, *J*_HH_ = 5.0, 2H, CH_2_N), 3.34 (d, *J*_HP_ = 4.7, 2H, CH_2_P), 3.59 (t, *J*_HH_ = 5.0, 2H, OCH_2_), 7.15 (s, 2H, C_4_H), 7.58–7.74 (d, *J*_HH_ = 11.4, 4H, C_2_H), [M + H]^+^_found_ = 346.1931, C_20_H_29_NO_2_P requires 346.1936.

*[(N-Ethyl)(N-2-hydroxyethyl)aminomethyl]diphenylphosphine oxide* (**10a**): Yield: 93% (0.32 g), white crystal; Mp: 81–82 °C; ^31^P NMR (CDCl_3_) δ: 28.5; ^13^C NMR (CDCl_3_) δ: 11.0 (NCH_2_*C*H_3_), 51.2 (d, ^3^*J*_CP_ = 6.2, N*C*H_2_CH_3_), 54.1 (d, ^1^*J*_CP_ = 90.2, CH_2_P), 58.0 (d, ^3^*J*_CP_ = 5.9, CH_2_N), 60.3 (OCH_2_), 128.6 (d, ^3^*J*_CP_ = 11.4, C_3_), 131.2 (d, ^2^*J*_CP_ = 8.9, C_2_), 131.7 (d, ^1^*J*_CP_ = 96.3, C_1_), 132.0 (d, *J*_CP_ = 2.8, C_4_); ^1^H NMR (CDCl_3_) δ: 0.83 (t, *J*_HH_ = 7.1, 3H, NCH_2_C*H*_3_), 2.55 (q, *J*_HH_ = 7.1, 2H, NC*H*_2_CH_3_), 2.84 (t, *J*_HH_ = 5.1, 2H, CH_2_C*H*_2_N), 3.53 (d, *J*_HP_ = 6.9, 2H, CH_2_P), 3.59 (t, *J*_HH_ = 5.0, 2H, OCH_2_), 7.41–7.61 (m, 6H, C_2_H, C_4_H), 7.68–7.88 (m, 4H, C_3_H); [M + H]^+^_found_ = 304.1457, C_17_H_23_NO_2_P requires 304.1466.

*[(N-Ethyl)(N-2-hydroxyethyl)aminomethyl]bis(p-tolyl)phosphine oxide* (**10b**): Yield: 91% (0.30 g), pale yellow viscous oil; ^31^P NMR (CDCl_3_) δ: 29.1; ^13^C NMR (CDCl_3_) δ: 11.0 (NCH_2_*C*H_3_), 21.6 (C_4_*C*H_3_), 51.2 (d, ^3^*J*_CP_ = 6.4, N*C*H_2_CH_3_), 54.1 (d, ^1^*J*_CP_ = 90.2, CH_2_P), 58.0 (d, ^3^*J*_CP_ = 5.8, CH_2_N), 60.3 (OCH_2_), 128.6 (d, ^1^*J*_CP_ = 99.0, C_1_), 129.4 (d, ^3^*J*_CP_ = 11.8, C_3_), 131.2 (d, ^2^*J*_CP_ = 8.9, C_2_), 142.5 (d, *J*_CP_ = 2.8, C_4_); ^1^H NMR (CDCl_3_) δ: 0.84 (t, *J*_HH_ = 7.1, 3H, NCH_2_C*H*_3_), 2.40 (s, 6H, C_4_CH_3_), 2.56 (q, *J*_HH_ = 7.1, 2H, NC*H*_2_CH_3_), 2.77 (t, *J*_HH_ = 5.0, 2H, CH_2_C*H*_2_N), 3.38 (d, *J*_HP_ = 4.2, 2H, CH_2_P), 3.60 (t, *J*_HH_ = 4.9, 2H, OCH_2_), 7.21–7.33 (m, 4H, C_2_H), 7.59–7.71 (m, 4H, C_3_H); [M + H]^+^_found_ = 332.1771, C_19_H_27_NO_2_P requires 332.1779.

*[(N-Ethyl)(N-2-hydroxyethyl)aminomethyl]bis(3,5-dimethylphenyl)phosphine oxide* (**10c**): Yield: 88% (0.32 g), pale yellow viscous oil; ^31^P NMR (CDCl_3_) δ: 29.1; ^13^C NMR (CDCl_3_) δ: 10.9 (NCH_2_*C*H_3_), 21.3 (C_3_*C*H_3_), 51.1 (d, ^3^*J*_CP_ = 6.0, N*C*H_2_CH_3_), 54.0 (d, ^1^*J*_CP_ = 89.4, CH_2_P), 57.9 (d, ^3^*J*_CP_ = 6.2, CH_2_N), 60.1 (OCH_2_), 128.7 (d, ^2^*J*_CP_ = 9.0, C_2_), 131.6 (d, ^1^*J*_CP_ = 95.9, C_1_), 133.7 (d, *J*_CP_ = 2.9, C_4_), 138.3 (d, ^3^*J*_CP_ = 12.0, C_3_); ^1^H NMR (CDCl_3_) δ: 0.86 (t, *J*_HH_ = 7.0, 3H, NCH_2_C*H*_3_), 2.35 (s, 12H, C_3_CH_3_), 2.57 (q, *J*_HH_ = 7.0, 2H, NC*H*_2_CH_3_), 2.76 (t, *J*_HH_ = 4.9, 2H, CH_2_C*H*_2_N), 3.40 (d, *J*_HP_ = 4.1, 2H, CH_2_P), 3.60 (t, *J*_HH_ = 4.9, 2H, OCH_2_), 7.15 (s, 2H, C_4_H) 7.37 (d, *J*_HH_ = 11.3, 4H, C_2_H); [M + H]^+^_found_ = 360.2075, C_21_H_31_NO_2_P requires 360.2092.

*[(N-Benzyl)(N-2-hydroxyethyl)aminomethyl]diphenylphosphine oxide* (**11a**): Yield: 90% (0.33 g), white crystal; Mp: 105–106 °C; ^31^P NMR (CDCl_3_) δ: 28.9; ^13^C NMR (CDCl_3_) δ: 53.9 (d, ^1^*J*_CP_ = 87.9, CH_2_P), 58.5 (d, ^3^*J*_CP_ = 4.7, CH_2_*C*H_2_N), 60.4 (OCH_2_), 61.7 (d, ^3^*J*_CP_ = 7.7, C_1_*C*H_2_N), 127.2 (C_4_), 128.3 (C_3_), 128.7 (d, ^3^*J*_CP_ = 11.5, C_3_′), 129.0 (C_2_), 131.1 (d, ^2^*J*_CP_ = 9.1, C_2_′), 131.7 (d, ^1^*J*_CP_ = 95.8, C_1_′), 132.0 (d, *J*_CP_ = 2.7, C_4_), 137.9 (C_1_); ^1^H NMR (CDCl_3_) δ: 2.88 (t, *J*_HH_ = 4.9, 2H, CH_2_C*H*_2_N), 3.51 (d, *J*_HP_ = 4.3, 2H, CH_2_P), 3.63 (t, *J*_HH_ = 5.0, 2H, OCH_2_), 3.67 (s, 2H, C_1_CH_2_N), 6.98–7.11 (m, 2H, C_2_H), 7.13–7.25 (m, 3H, C_3_H, C_4_H), 7.38–7.57 (m, 6H, C_2_′H, C_4_′H), 7.61–7.78 (m, 4H, C_3_′H); [M + H]^+^_found_ = 366.1615, C_22_H_25_NO_2_P requires 366.1623.

*[(N-Benzyl)(N-2-hydroxyethyl)aminomethyl]bis(p-tolyl)phosphine oxide* (**11b**): Yield: 89% (0.35 g), pale yellow viscous oil; ^31^P NMR (CDCl_3_) δ: 29.5; ^13^C NMR (CDCl_3_) δ: 21.6 (C_4_′*C*H_3_), 54.0 (d, ^1^*J*_CP_ = 88.1, CH_2_P), 58.4 (d, ^3^*J*_CP_ = 4.9, CH_2_*CH*_2_N), 60.3 (OCH_2_), 61.6 (d, ^3^*J*_CP_ = 7.5, C_1_*C*H_2_N), 127.2 (C_4_), 127.8 (C_3_), 128.3 (C_2_), 128.5 (d, ^1^*J*_CP_ = 99.4, C_1_′), 129.4 (d, ^3^*J*_CP_ = 11.9, C_3_′), 131.1 (d, ^2^*J*_CP_ = 9.4, C_2_′), 138.0 (C_1_), 142.4 (d, *J*_CP_ = 2.8, C_4_); ^1^H NMR (CDCl_3_) δ: 2.38 (s, 6H, C_4_CH_3_), 2.87 (t, *J*_HH_ = 4.8, 2H, CH_2_C*H*_2_N), 3.46 (d, *J*_HP_ = 4.5, 2H, CH_2_P), 3.62 (t, *J*_HH_ = 5.0, 2H, OCH_2_), 3.67 (s, 2H, C_1_CH_2_N), 7.00–7.11 (m, 2H, C_2_H), 7.13–7.36 (m, 7H, C_3_H, C_4_H, C_2_′H), 7.47–7.66 (m, 4H, C_3_′H); [M + H]^+^_found_ = 394.1926, C_24_H_29_NO_2_P requires 394.1936.

*[(N-Benzyl)(N-2-hydroxyethyl)aminomethyl]bis(3,5-dimethylphenyl)phosphine oxide* (**11c**): Yield: 92% (0.39 g), pale yellow viscous oil; ^31^P NMR (CDCl_3_) δ: 29.2; ^13^C NMR (CDCl_3_) δ: 21.3 (C_3_*C*H_3_), 53.9 (d, ^1^*J*_CP_ = 87.3, CH_2_P), 58.5 (d, ^3^*J*_CP_ = 5.0, CH_2_C*H*_2_N), 60.3 (OCH_2_), 61.7 (d, ^3^*J*_CP_ = 7.1, C_1_*C*H_2_N), 127.1 (C_4_), 128.2 (C_3_), 128.6 (d, ^2^*J*_CP_ = 9.0, C_2_′), 128.9 (C_2_), 131.6 (d, ^1^*J*_CP_ = 96.0, C_1_′), 133.7 (d, *J*_CP_ = 2.7, C_4_), 138.2 (C_1_), 138.4 (d, ^3^*J*_CP_ = 12.1, C_3_′); ^1^H NMR (CDCl_3_) δ: 2.32 (s, 12H, C_3_CH_3_), 2.87 (t, *J*_HH_ = 4.7, 2H, CH_2_C*H*_2_N), 3.48 (d, *J*_HP_ = 4.1, 2H, CH_2_P), 3.63 (t, *J*_HH_ = 5.0, 2H, OCH_2_), 3.67 (s, 2H, C_1_CH_2_N), 7.03–7.16 (m, 4H, C_2_H, C_4_′H), 7.17–7.24 (m, 3H, C_3_H, C_4_H), 7.31 (d, *J*_HH_ = 11.4, 4H, C_2_′H); [M + H]^+^_found_ = 422.2240, C_26_H_33_NO_2_P requires 422.2249.

### 3.4. General Procedure for the Synthesis of the N,N-Bis(diarylphosphinoylmethyl)ethanolamines

The mixture of 1.0 mmol of ethanolamine (0.06 mL), 3.0 mmol of paraformaldehyde (0.09 g), and 3.0 mmol of secondary phosphine oxide [diphenylphosphine oxide (0.61 g), bis(*p*-tolyl)phosphine oxide (0.69 g), or bis(3,5-dimethylphenyl)phosphine oxide (0.77 g)] in 2 mL of acetonitrile was irradiated in a sealed tube at 120 °C for 1 h in a CEM Discover Microwave reactor equipped with a pressure controller. The crude product was purified by flash column chromatography using silica gel and dichloromethane–methanol 9:1 as the eluent. Thus, the following products were prepared:

*N,N-Bis(diphenylphosphinoylmethyl)ethanolamine* (**12a**): Yield: 95% (0.46 g), white crystal; Mp: 146–147 °C; ^31^P NMR (CDCl_3_) δ: 28.9; ^13^C NMR (CDCl_3_) δ: 55.5 (dd, ^1^J_CP_ = 81.2, ^3^*J*_CP_ = 5.7, CH_2_P), 59.9 (HOCH_2_), 60.3 (t, ^3^*J*_CP_ = 5.3, CH_2_N), 128.6 (m, C_3_), 131.1 (m, C_2_), 131.8 (d, ^1^*J*_CP_ = 96.3, C_1_), 132.0 (brs, C_4_); ^1^H NMR (CDCl_3_) δ: 3.05 (t, *J*_HH_ = 4.9, 2H, CH_2_N), 3.56 (t, *J*_HH_ = 4.9, 2H, HOC*H*_2_), 3.82 (d, *J*_HP_ = 3.9, 4H, CH_2_P), 7.33–7.56 (m, 12H, C_2_H, C_4_H) 7.64–7.82 (m, 8H, C_3_H); [M + H]^+^_found_ = 490.1685, C_28_H_30_NO_3_P_2_ requires 490.1701.

*N,N-Bis(di-p-tolyl)phosphinoylmethyl)ethanolamine* (**12b**): Yield: 93% (0.51 g), white crystal^*^; ^31^P NMR (CDCl_3_) δ: 29.4; ^13^C NMR (CDCl_3_) δ: 21.6 (C_4_CH_3_), 55.5 (dd, ^1^*J*_CP_ = 81.2, ^3^*J*_CP_ = 5.5, CH_2_P), 59.9 (HOCH_2_), 60.3 (t, ^3^*J*_CP_ = 4.9, CH_2_N), 128.8 (d, ^1^*J*_CP_ = 98.5, C_1_), 129.3 (m, C_3_), 131.1 (m, C_2_), 142.3 (brs, C_4_); ^1^H NMR (CDCl_3_) δ: 2.36 (s, 12H, C_4_CH_3_), 3.02 (t, *J*_HH_ = 5.0, 2H, CH_2_N), 3.54 (t, *J*_HH_ = 4.7, 2H, HOC*H*_2_), 3.73 (d, *J*_HP_ = 3.7, 4H, CH_2_P), 7.07–7.32 (m, 8H, C_2_H) 7.46–7.70 (m, 8H, C_3_H); [M + H]^+^_found_ = 546.2308, C_32_H_38_NO_3_P_2_ requires 546.2326. *No sharp melting point was observed.

*N,N-Bis(3,5-dimethylphenylphosphinoylmethyl)ethanolamine* (**12c**): Yield: 91% (0.55 g), pale yellow crystal; Mp: 128–129 °C; ^31^P NMR (CDCl_3_) δ: 28.6; ^13^C NMR (CDCl_3_) δ: 21.3 (C_3_*C*H_3_), 55.0 (dd, ^1^*J*_CP_ = 78.8, ^3^*J*_CP_ = 3.9, CH_2_P), 59.9 (HOCH_2_), 60.4 (t, ^3^*J*_CP_ = 4.3, CH_2_N), 128.5 (m, C_2_), 132.2 (d, ^1^*J*_CP_ = 98.1, C_1_), 133.6 (brs, C_4_), 138.3 (m, C_3_); ^1^H NMR (CDCl_3_) δ: 2.29 (s, 24H, C_3_CH_3_), 2.99 (t, *J*_HH_ = 4.8, 2H, CH_2_N), 3.57 (t, *J*_HH_ = 4.9, 2H, HOC*H*_2_), 3.75 (s, 4H, CH_2_P), 7.09 (s, 4H, C_4_H) 7.32 (d, *J*_HH_ = 11.3, 8H, C_2_H); [M + H]^+^_found_ = 602.2926, C_36_H_46_NO_3_P_2_ requires 602.2952.

### 3.5. Crystal Structure Determination

Single-crystal X-ray diffraction data of **11a** and **12a**∙H_2_O were collected on an Agilent Technologies SuperNova Dual diffractometer using Mo-Kα radiation (λ = 0.71073 Å) at room temperature. The data were processed using CrysAlis Pro [33]. The structures were solved by ShelXT [34] using intrinsic phasing and refined by a full-matrix least-squares procedure based on *F*^2^ with ShelXL [35] using Olex2 program suite [36]. All the non-hydrogen atoms were refined anisotropically. Hydrogen atoms were readily located in difference Fourier maps, and were subsequently treated as riding atoms in geometrically idealized positions with C–H = 0.93 Å (aromatic) or 0.97 Å (methylene), O–H = 0.82 Å, and with *U*_iso_(H) = *kU*_eq_(C,O), where *k* = 1.5 for the hydroxyl group and 1.2 for all the H atoms bonded to C atoms, unless otherwise noted. In **12a,** H_2_O H atoms bonded to water solvate molecule O4 were refined restraining the bonding distances with *U*_iso_(H) = 1.5 *U*_eq_(O). Crystal structure data are deposited with the Cambridge Crystallographic Data Centre under CCDC 1,906,625 (**11a**) and 1,906,626 (**12a**∙H_2_O), and can be obtained free of charge via http://www.ccdc.cam.ac.uk/conts/retrieving.html (or from the CCDC, 12 Union Road, Cambridge CB2 1EZ, UK; Fax: +44 1223 336033; E-mail: deposit@ccdc.cam.ac.uk).

## 4. Conclusions

In summary, we have developed a facile, catalyst-free and mostly solvent-free MW-assisted method for the synthesis of *N*-2-hydroxyethyl-α-aminophosphonates and *N*-2-hydroxyethyl-α-aminophosphine oxides, as well as *N,N*-bis(diarylphosphinoylmethyl)ethanolamines by the three-component reaction of amino alcohols, paraformaldehyde, and dialkyl phosphites or diarylphosphine oxides. This method is a novel approach for the preparation of *N*-2-hydroxyethyl-α-aminophosphine oxides and *N,N*-bis(diarylphosphinoylmethyl)ethanolamines. Altogether, 21 derivatives were synthesized and fully characterized, and all of them are new compounds. The crystal structure of [(*N*-benzyl)(*N*-2-hydroxyethyl)aminomethyl]diphenylphosphine oxide (**11a**) and *N*,*N*-bis(diphenylphosphinoylmethyl)ethanolamine (**12a**) was studied by single-crystal XRD analysis.

## Figures and Tables

**Table 1:**
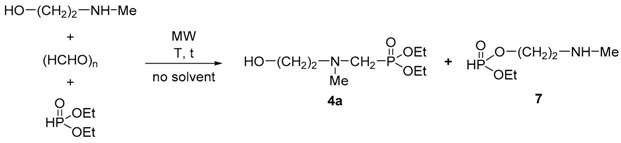
Optimization of the condensation of *N*-methylethanolamine, paraformaldehyde, and diethyl phosphite.

Entry	Mode of Heating	T [°C]	t [min]	Conversion [%] ^a^	Product Composition [%] ^a^	
					4a	7
1	MW	60	20	85	96	4
2	MW	80	20	100	95	5
3	Δ	80	20	96	81	19
4	MW	100	10	100	90	10

^a^ Based on relative ^31^P NMR intensities.

**Table 2:**
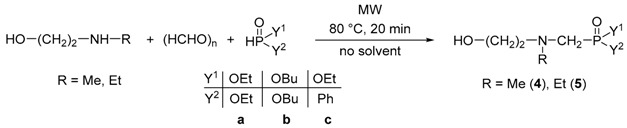
Condensation of *N*-alkylethanolamines, paraformaldehyde, and dialkyl phosphites or ethyl phenyl-*H*-phosphinate.

Entry	R	Y^1^	Y^2^	Yield [%] ^a^
1	Me	OEt	OEt	78 (**4a**)
2	Me	OBu	OBu	84 (**4b**)
3	Me	OEt	Ph	67 (**4c**)
4	Et	OEt	OEt	72 (**5a**)
5	Et	OBu	OBu	79 (**5b**)
6	Et	OEt	Ph	64 (**5c**)

^a^ After column chromatography.

**Table 3:**
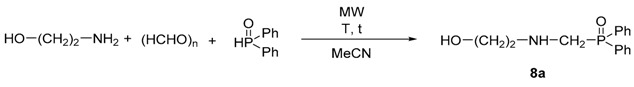
Optimization of the condensation of ethanolamine, paraformaldehyde, and diphenylphosphine oxide.

Entry	T [°C]	t [min]	Conversion [%] ^a^
1	80	20	58
2	80	30	86
3	80	40	89
4	100	20	92
5	100	30	100

^a^ Based on HPLC (254 nm).

**Table 4:**
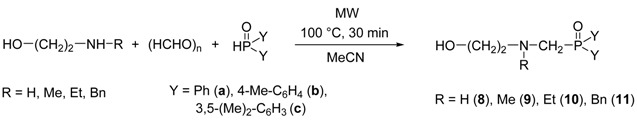
Condensation of amino alcohols, paraformaldehyde, and secondary phosphine oxides.

Entry	R	Y	Yield [%] ^a^
1	H	Ph	96 (**8a**)
2	H	4-Me-C_6_H_4_	89 (**8b**)
3	H	3,5-(Me)_2_-C_6_H_3_	95 (**8c**)
4	Me	Ph	95 (**9a**)
5	Me	4-Me-C_6_H_4_	96 (**9b**)
6	Me	3,5-(Me)_2_-C_6_H_3_	93 (**9c**)
7	Et	Ph	95 (**10a**)
8	Et	4-Me-C_6_H_4_	93 (**10b**)
9	Et	3,5-(Me)_2_-C_6_H_3_	88 (**10c**)
10	Bn	Ph	90 (**11a**)
11	Bn	4-Me-C_6_H_4_	89 (**11b**)
12	Bn	3,5-(Me)_2_-C_6_H_3_	92 (**11c**)

^a^ After column chromatography.

**Table 5:**
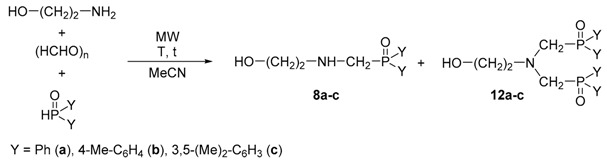
Double Kabachnik–Fields reaction of ethanolamine using excess of the paraformaldehyde and the secondary phosphine oxides.

Entry	Y	(HCHO)_n_ [equiv]	Y_2_P(O)H [equiv]	T [°C]	t [h]	Product Composition [%] ^a^		Yield ^b^ [%]
						8a–c	12a–c	
1	Ph	2	2	100	1	75	25	-
2	Ph	2	2	120	1	66	34	-
3	Ph	2	2	120	1.5	64	36	-
4	Ph	2	2.5	120	1	62	38	-
5	Ph	2.5	2.5	120	1	25	75	-
6	Ph	3	3	120	1	0	100	95 (**12a**)
7	4-Me-C_6_H_4_	3	3	120	1	0	100	93 (**12b**)
8	3,5-(Me)_2_-C_6_H_3_	3	3	120	1	0	100	91 (**12c**)

^a^ Based on HPLC (254 nm). ^b^ After column chromatography.

## Supplementary Materials

Supplementary data associated with this article are available online. X-ray crystallographic data and copies of ^31^P, ^1^H, and ^13^C NMR spectra for all compounds synthesized are presented. Figure S1: (**a**) Chain formation via C–H∙∙∙O hydrogen bonding in **11a**. (**b**) Layer formation via C–H∙∙∙π interactions. Figure S2: (**a**) Layer formation via O–H∙∙∙O hydrogen bonding in **12a**∙H_2_O along *ab*-plane. (**b**) Packing of layers along c-axis. Table S1: Hydrogen bond geometry for **11a** and **12a**∙H_2_O. Table S2: Essential crystallographic data of the **11a** and **12a****∙**H_2_O single-crystal diffraction experiments and model refinements.
