# Supplementary material for: Microwave-Assisted Kabachnik–Fields Reaction with Amino Alcohols as the Amine Component
Source: Molecules. 2019 Apr 25;24(8):1640. doi: 10.3390/molecules24081640 (PMC6514811; doi:10.3390/molecules24081640)
Supplement: Supplementary file 1 [file molecules-24-01640-s001.pdf]

Supplementary Material

for

Microwave-Assisted Kabachnik–Fields Reaction with Amino Alcohols as the Amine Component

Ádám Tajti [1], Enikő Szatmári [1], Franc Perdih [2], György Keglevich [1] and Erika Bálint [1]*

Address:

[1]Department of Organic Chemistry and Technology, Budapest University of Technology and Economics, 1521 Budapest, Hungary

[2]Faculty of Chemistry and Chemical Technology, University of Ljubljana, SI-1000 Ljubljana, Slovenia

E-mail:

Erika Bálint* - ebalint@mail.bme.hu

* Corresponding author

Table of contents

Single-crystal X-ray diffraction measurements S2–S3

^{31}P NMR, ^{1}H NMR and ^{13}C NMR spectra S4–S35

Single crystal X-ray diffraction measurements

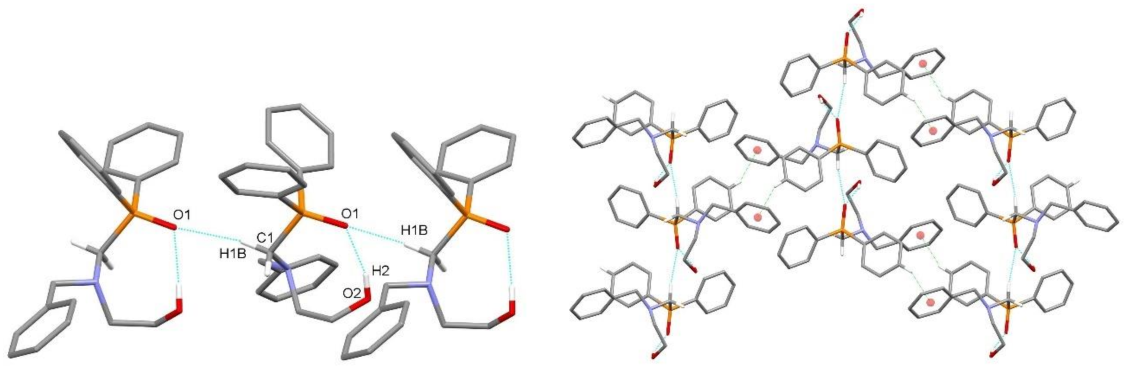

(**a**) (**b**)

Figure S1. (**a**) Chain formation *via* C–H···O hydrogen bonding in **11a** (blue dashed lines). (**b**) Layer formation *via* C–H···π interactions. Hydrogen atoms not involved in the motif shown have been omitted for clarity.

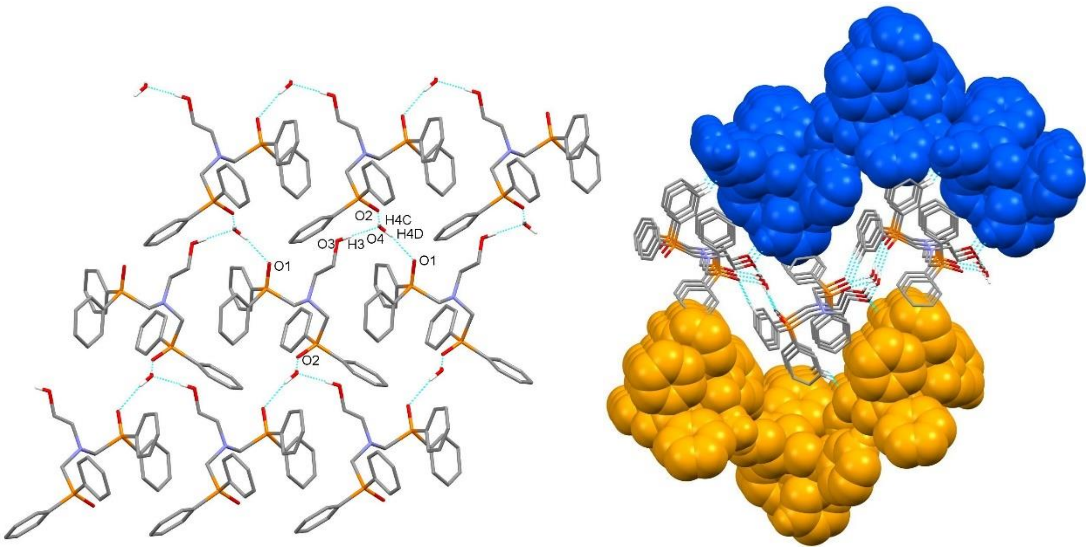

(**a**) (**b**)

Figure S2. (**a**) Layer formation *via* O–H···O hydrogen bonding in **12a**·H_2O along *ab*-plane (blue dashed lines). (**b**) Packing of layers along c-axis. Hydrogen atoms not involved in the motif shown have been omitted for clarity.

Table S1. Hydrogen bond geometry for **11a** and **12a**·H_2O.

D–H···A	D–H (Å)	H···A (Å)	D···A (Å)	D–H···A (°)	Symmetry code
**11a**					
O2–H2···O1	0.82	2.04	2.824(2)	159	*x*, *y*, *z*
C1–H1B···O1	0.97	2.33	3.280(2)	168	–½ + *x*, 1½ – *y*, –½ + *z*
C20–H20···*Cg*1	0.93	2.74	3.603(2)	155	1 – *x*, 1 – *y*, –*z*
**12a**·H_2O					
O3–H3···O4	0.82	1.90	2.718(3)	179	*x*, *y*, *z*
O4–H4C···O2	0.83(2)	1.92(2)	2.737(3)	169(3)	1 – *x*, ½ + *y*, ½ – *z*
O4–H4D···O1	0.80(3)	1.98(3)	2.775(3)	171(3)	–1 + *x*, *y*, *z*
C19–H19···O1	0.93	2.46	3.371(4)	167	1 – *x*, –½ + *y*, ½ – *z*
C25–H25···O3	0.93	2.55	3.398(4)	152	1– *x*, 1 – *y*, 1 – *z*

*Cg*1 is a C5–C10 ring centroid.

Table S2. Essential crystallographic data of the **11a** and **12a·**H_2O single-crystal diffraction experiments and model refinements

	**11a**	**12a·**H_2O
**Formula**	$C_{22}H_{24}NO_2P$	$C_{28}H_{31}NO_4P_2$
**F. W.**	365.39	507.48
**Space grp.**	$P2_1/n$ (no. 14)	$P2_1/c$ (no. 14)
a, b, c **[Å]**	9.9927(7), 20.5357(10), 10.4303(7)	8.1148(4), 16.1422(10), 20.2089(9)
β **[º]**	111.642(8)	91.644(4)
V **[Å^3]**	1989.5(2)	2646.1(2)
**Z**	4	4
D_{calc} **[g/cm^3]**	1.220	1.274
μ **[(MoKα)/mm]**	0.153	0.198
**Cryst. Size [mm]**	0.30 x 0.20 x 0.10	0.25 x 0.15 x 0.15
**Temp. (K)**	293(2)	293(2)
**Radiation λ [Å]**	MoKa 0.71073	MoKa 0.71073
θ **Min-Max [º]**	5.218, 54.954	4.758, 54.966
**Tot., Uniq. Data,** R_{int}**,** R_{sigma}	10276, 4550, 0.0243, 0.0333	12825, 6081, 0.0275, 0.0391
**Obsd data [*I* > 2.0 *σ*(*I*)]**	3531	4408
**Nref, Npar**	4550, 236	6081, 323
R_1**,** wR^2	0.0419, 0.0974	0.0521, 0.1473
**Max./Av. Shift/Error**	0.00, 0.00	0.00, 0.00
**Min/Max Res.Den. [e/Å^3]**	-0.27, 0.27	-0.26, 0.45

^{31}P NMR, ^{13}C NMR and ^{1}H NMR spectra of compounds

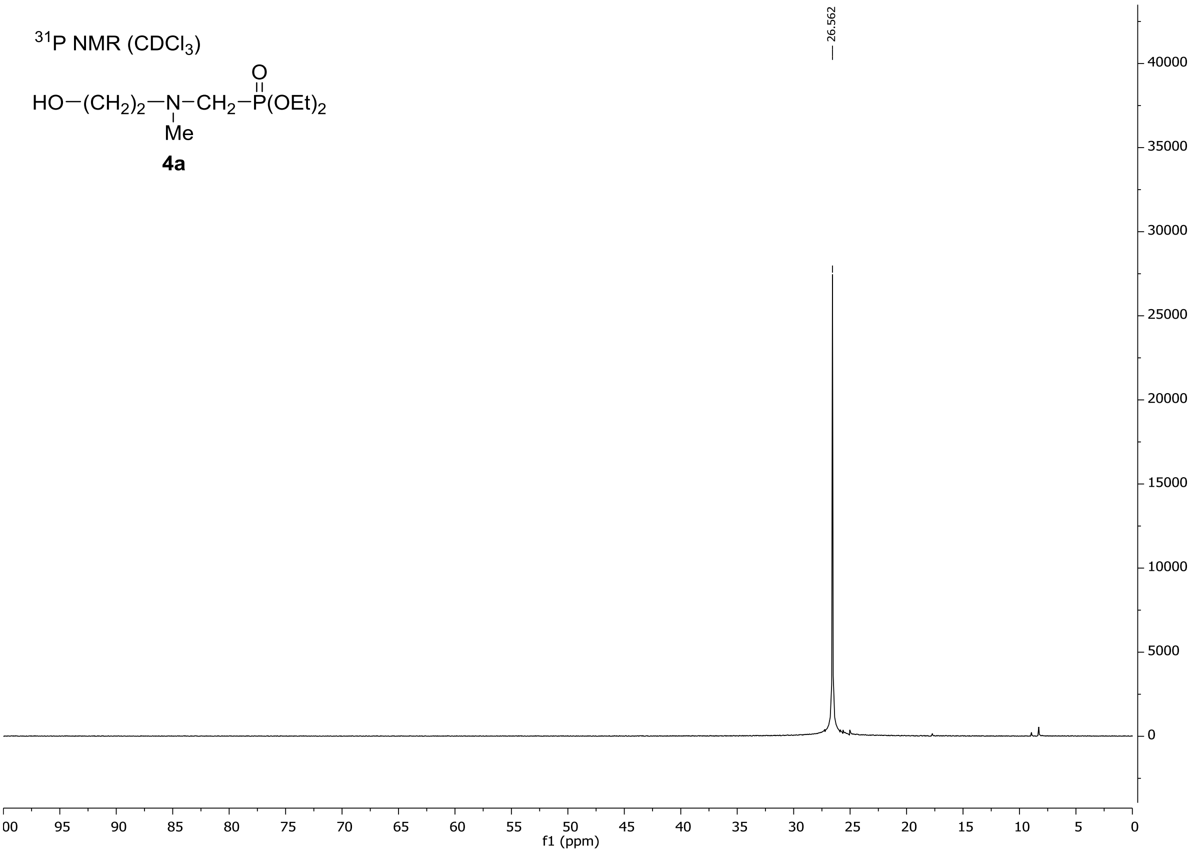

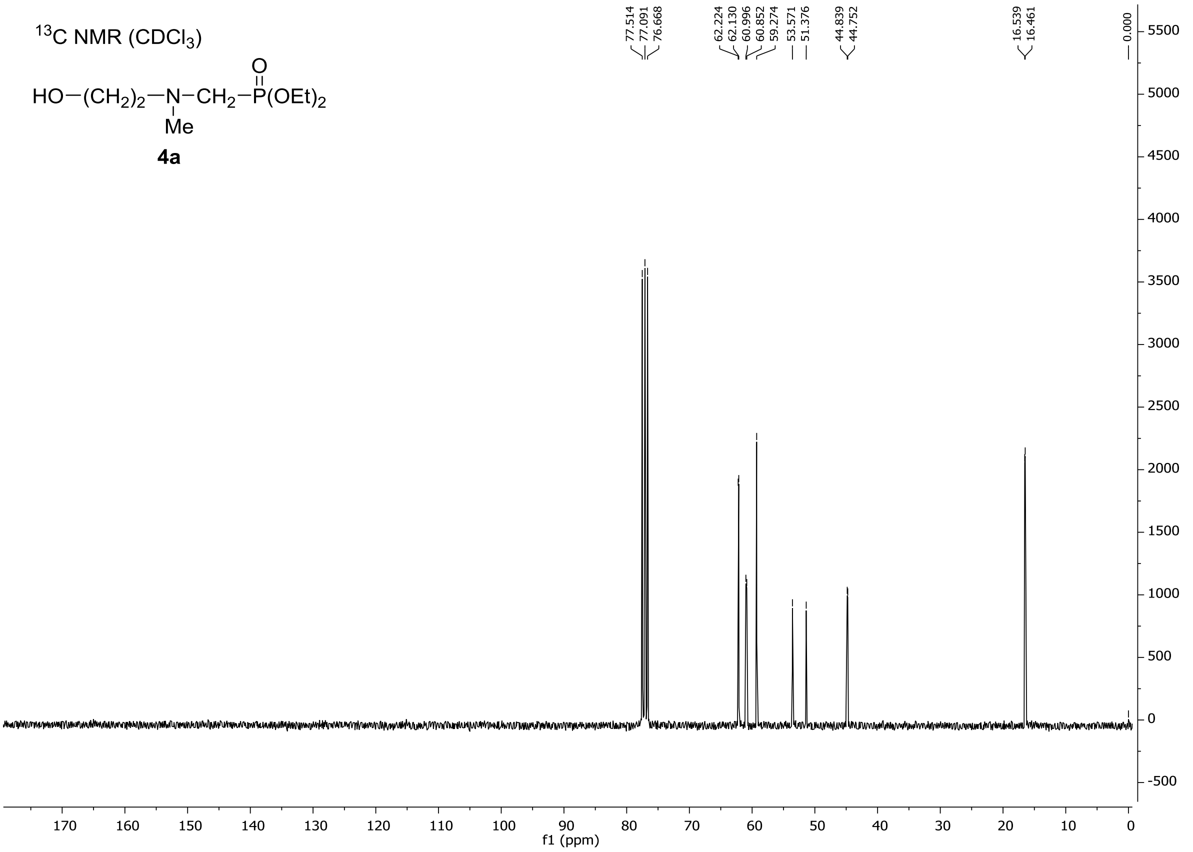

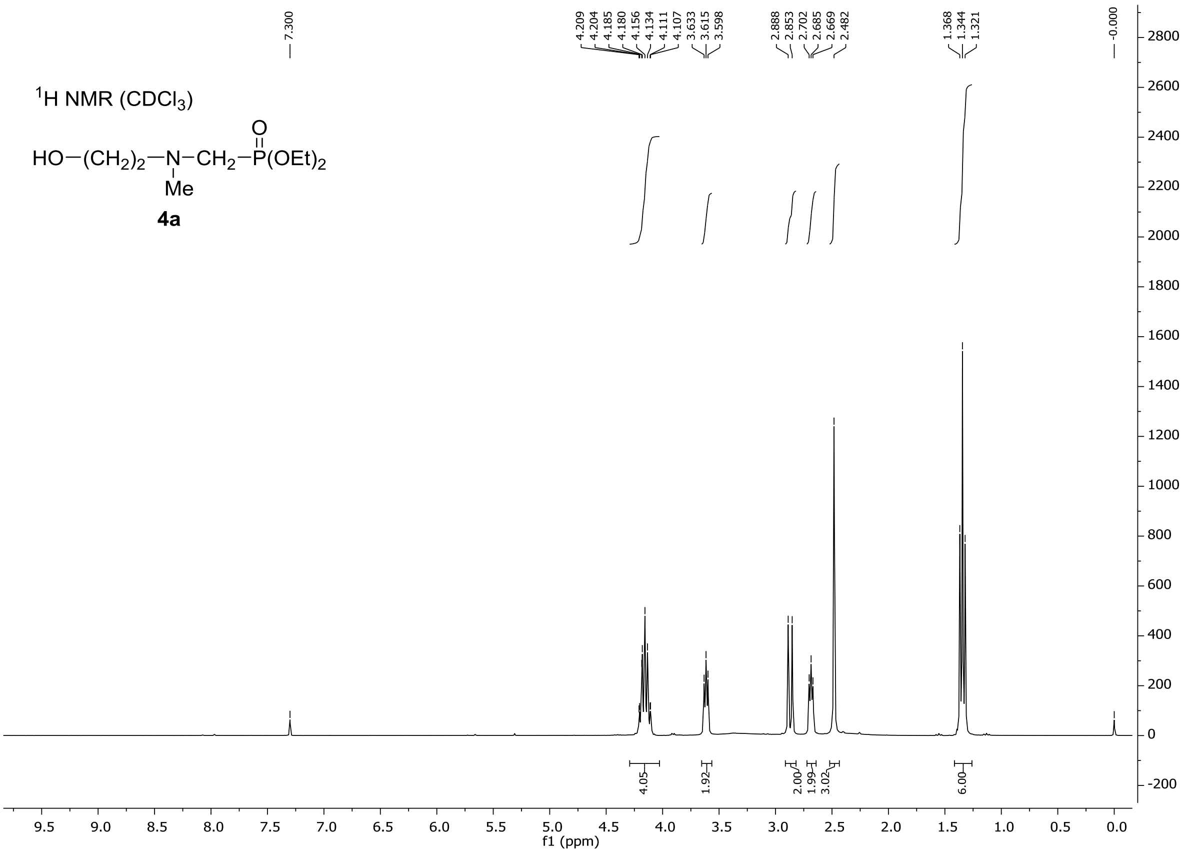

^{1}H NMR (CDCl$_3$)
HO−(CH$_2$)$_2$−N(Me)−CH$_2$−P(O)(OEt)$_2$
4a
f1 (ppm)

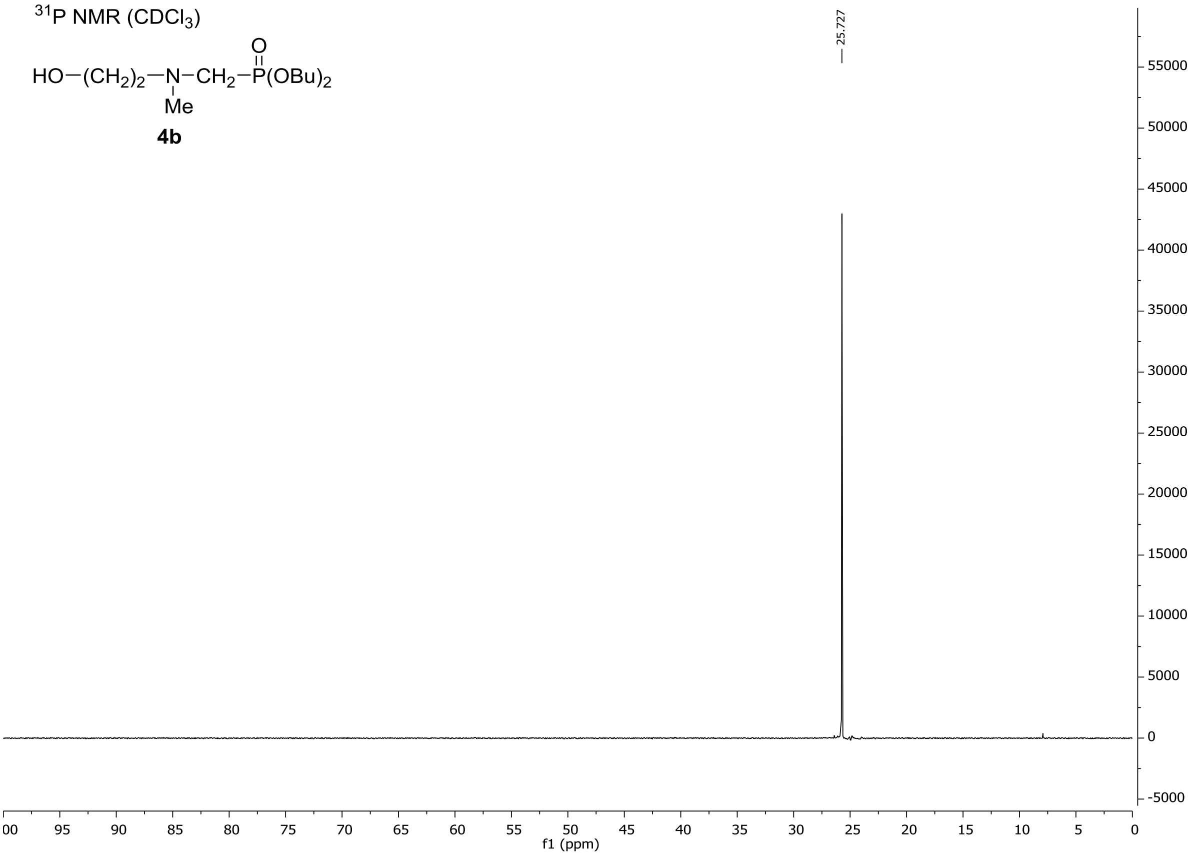

^{31}P NMR (CDCl$_3$)
HO−(CH$_2$)$_2$−N(Me)−CH$_2$−P(O)(OBu)$_2$
4b
25.727
f1 (ppm)

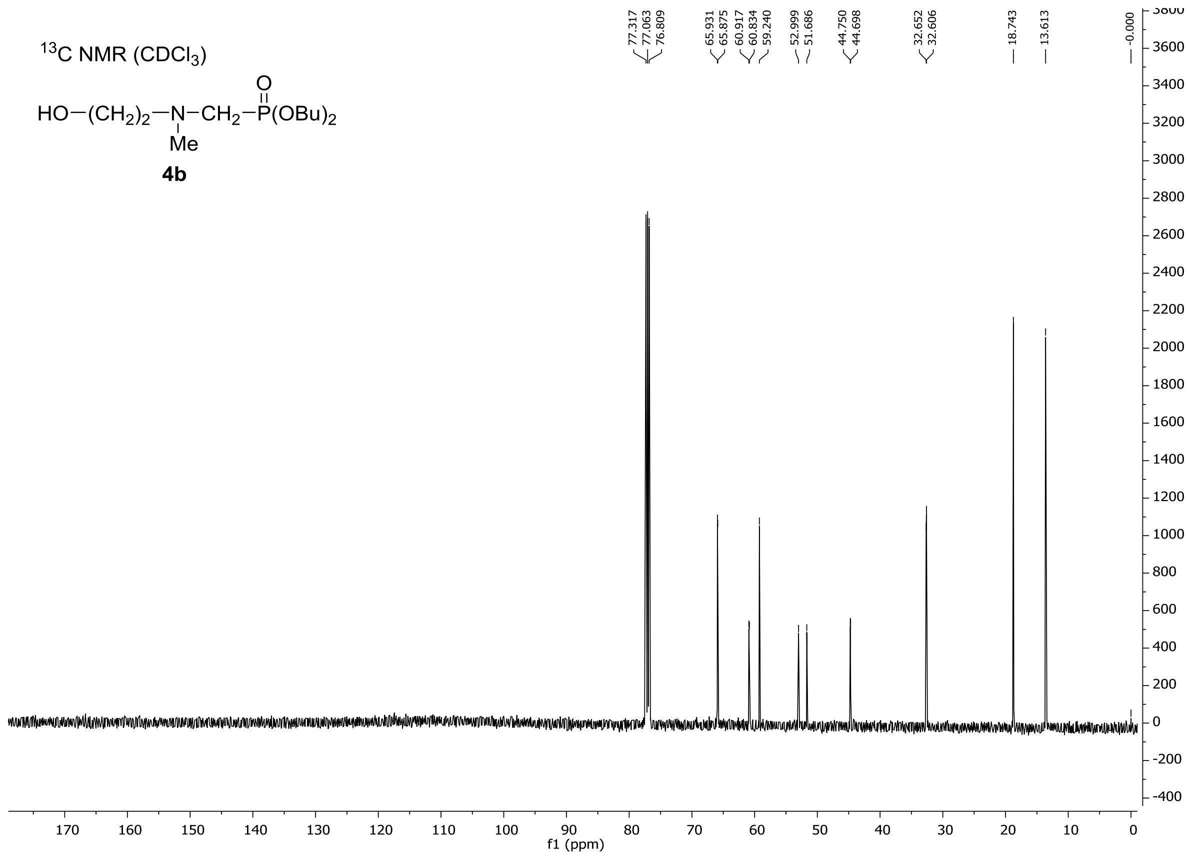

^{13}C NMR (CDCl$_3$)
HO−(CH$_2$)$_2$−N(Me)−CH$_2$−P(O)(OBu)$_2$
4b
77.317
77.063
76.809
65.931
65.875
60.917
60.834
59.240
52.999
51.686
44.750
44.698
32.652
32.606
18.743
13.613
-0.000
f1 (ppm)

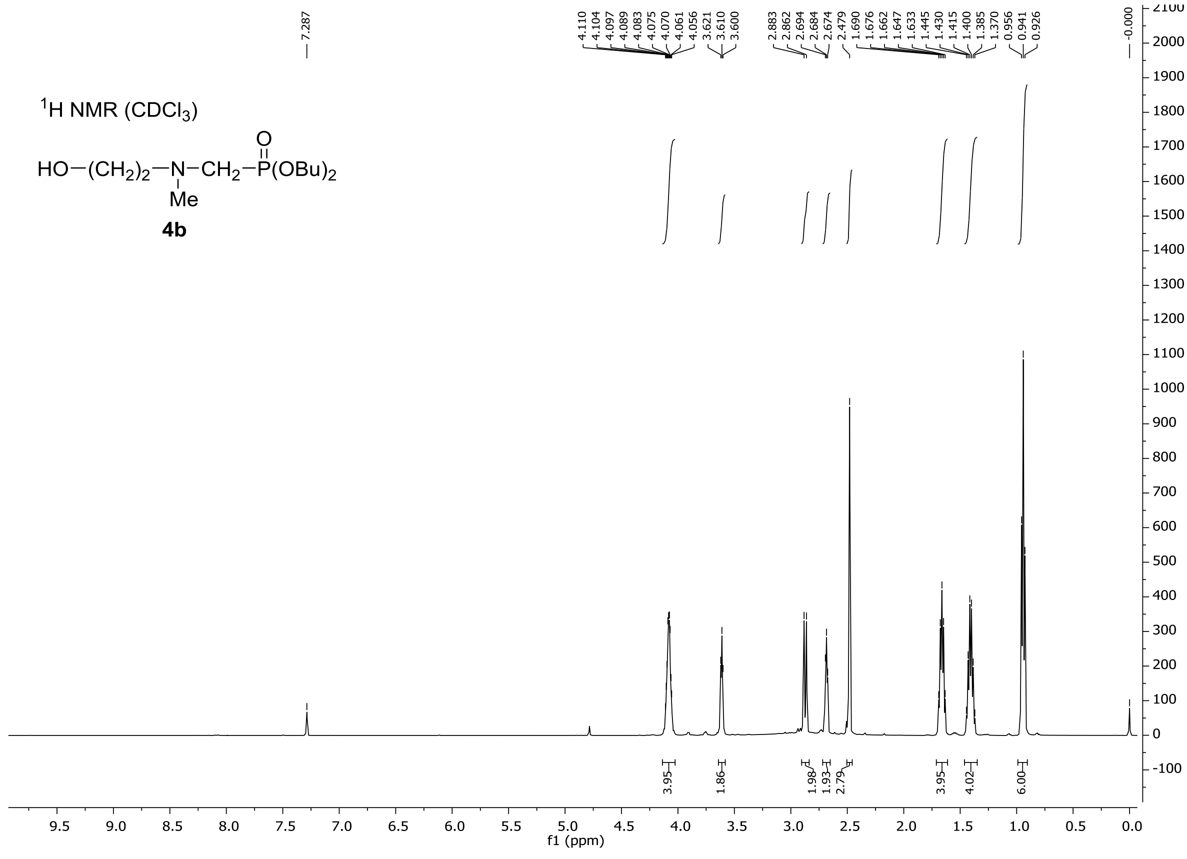

^{1}H NMR (CDCl$_3$)
HO−(CH$_2$)$_2$−N(Me)−CH$_2$−P(O)(OBu)$_2$
4b
7.287
4.110
4.104
4.097
4.089
4.083
4.075
4.070
4.061
4.056
3.621
3.610
3.600
2.883
2.862
2.694
2.684
2.674
2.479
1.690
1.676
1.662
1.647
1.633
1.445
1.430
1.415
1.400
1.385
1.370
0.956
0.941
0.926
-0.000
3.95
1.86
1.98
1.93
2.79
3.95
4.02
6.00
f1 (ppm)

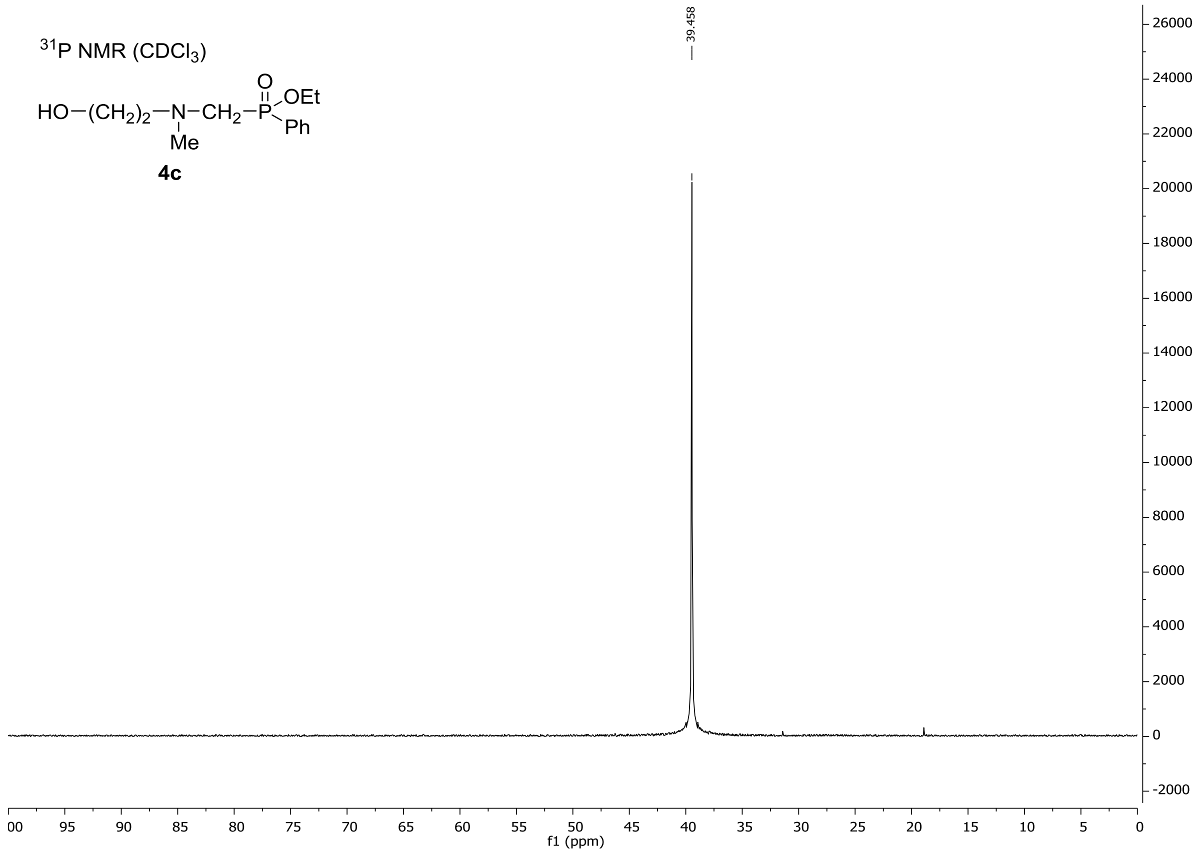
^{31}P NMR ($CDCl_3$)
HO−(CH_2)$_2$−N(Me)−CH_2−P(O)(OEt)Ph
4c
39.458
f1 (ppm)

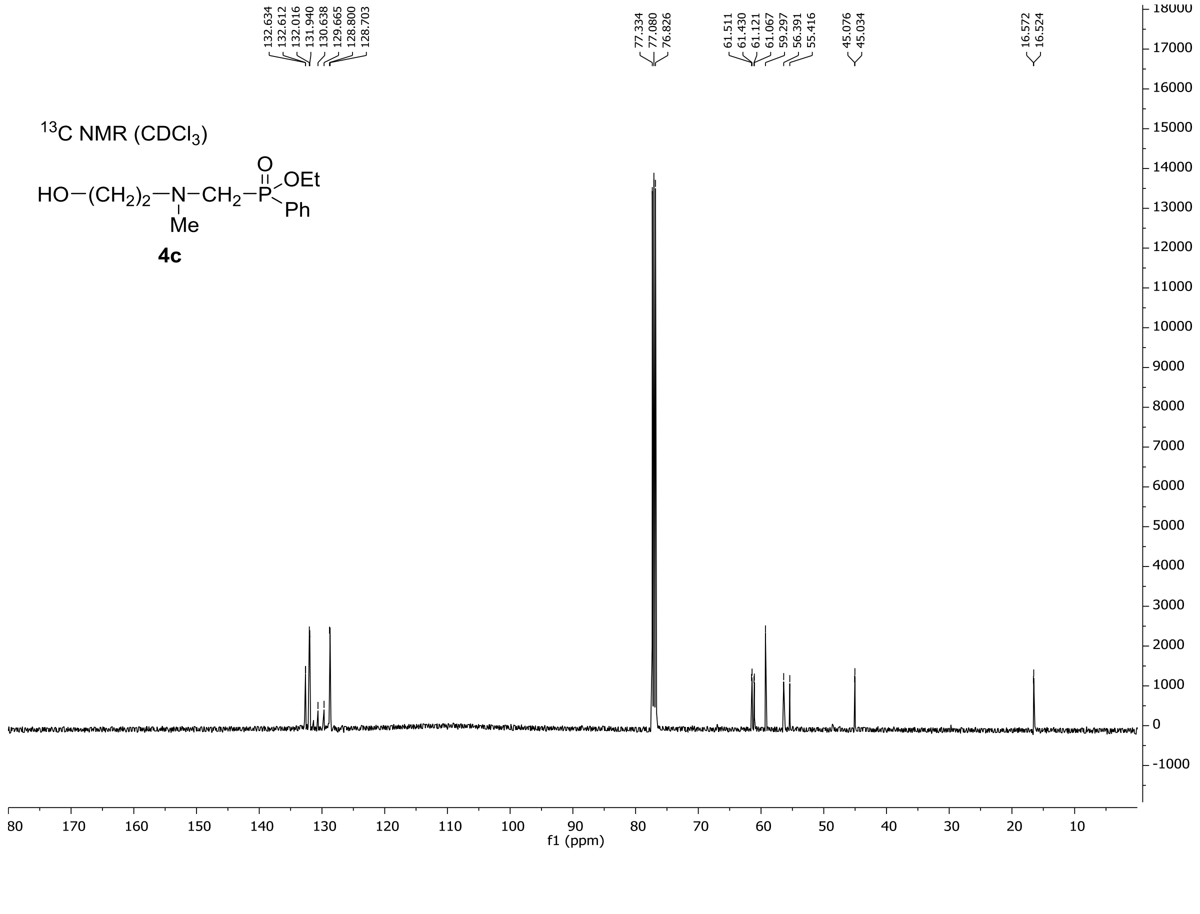
^{13}C NMR ($CDCl_3$)
HO−(CH_2)$_2$−N(Me)−CH_2−P(O)(OEt)Ph
4c
132.634
132.612
132.016
131.940
130.638
129.665
128.800
128.703
77.334
77.080
76.826
61.511
61.430
61.121
61.067
59.297
56.391
55.416
45.076
45.034
16.572
16.524
f1 (ppm)

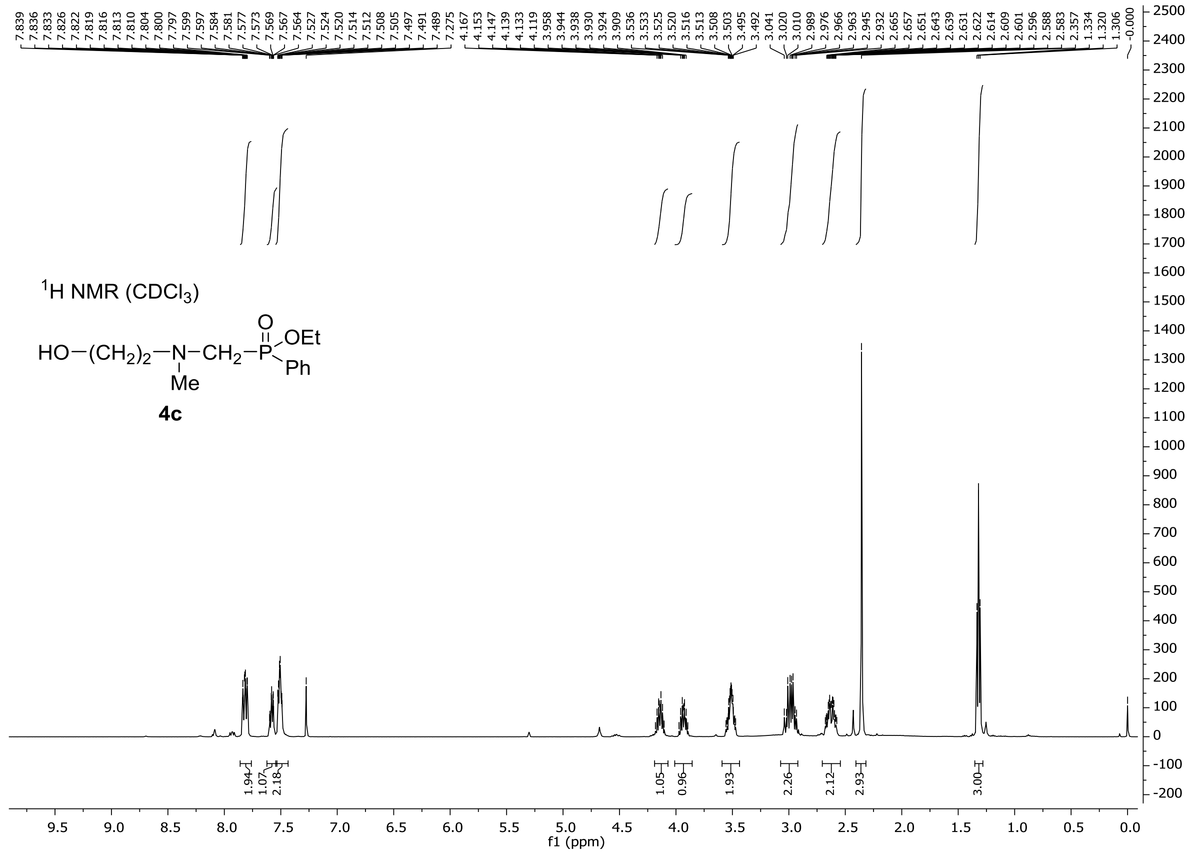
1H NMR (CDCl3)
4c
f1 (ppm)

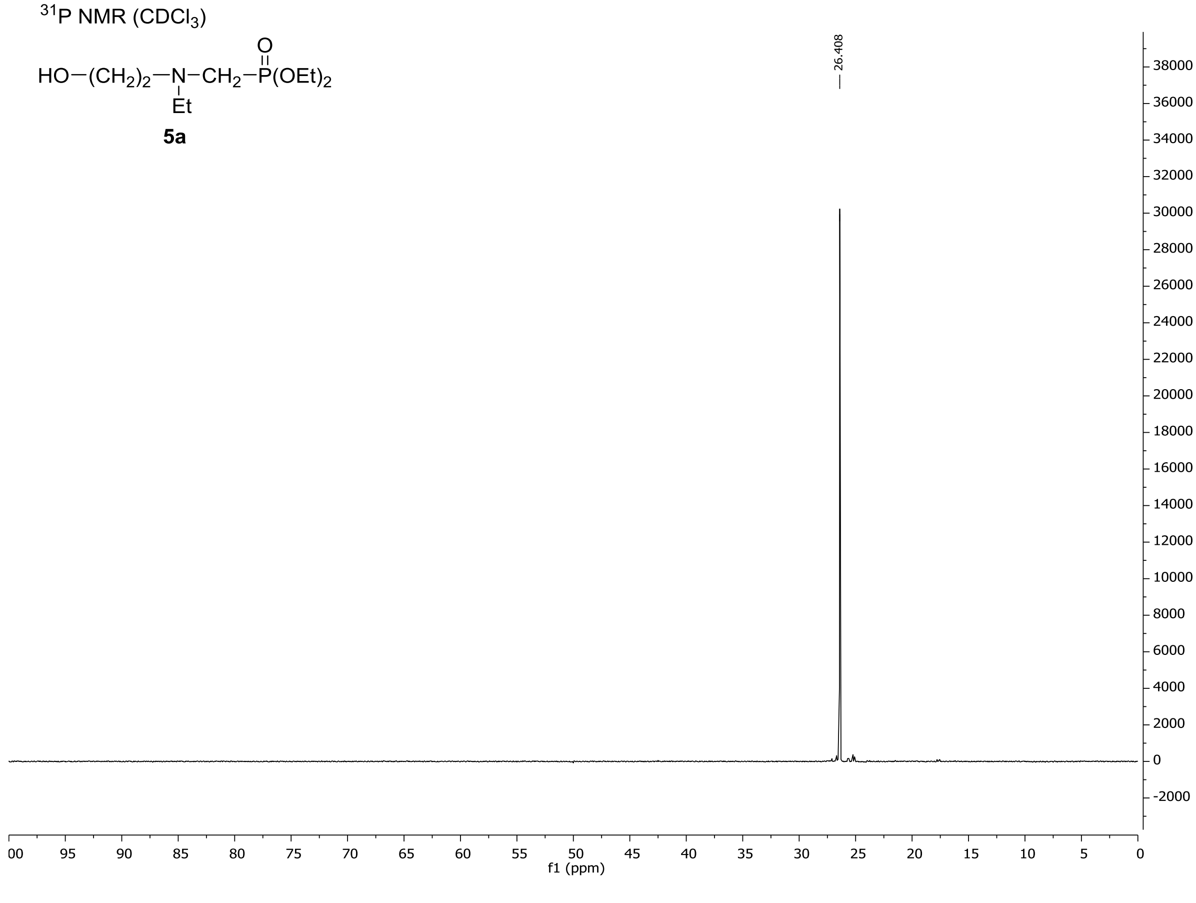
31P NMR (CDCl3)
5a
26.408
f1 (ppm)

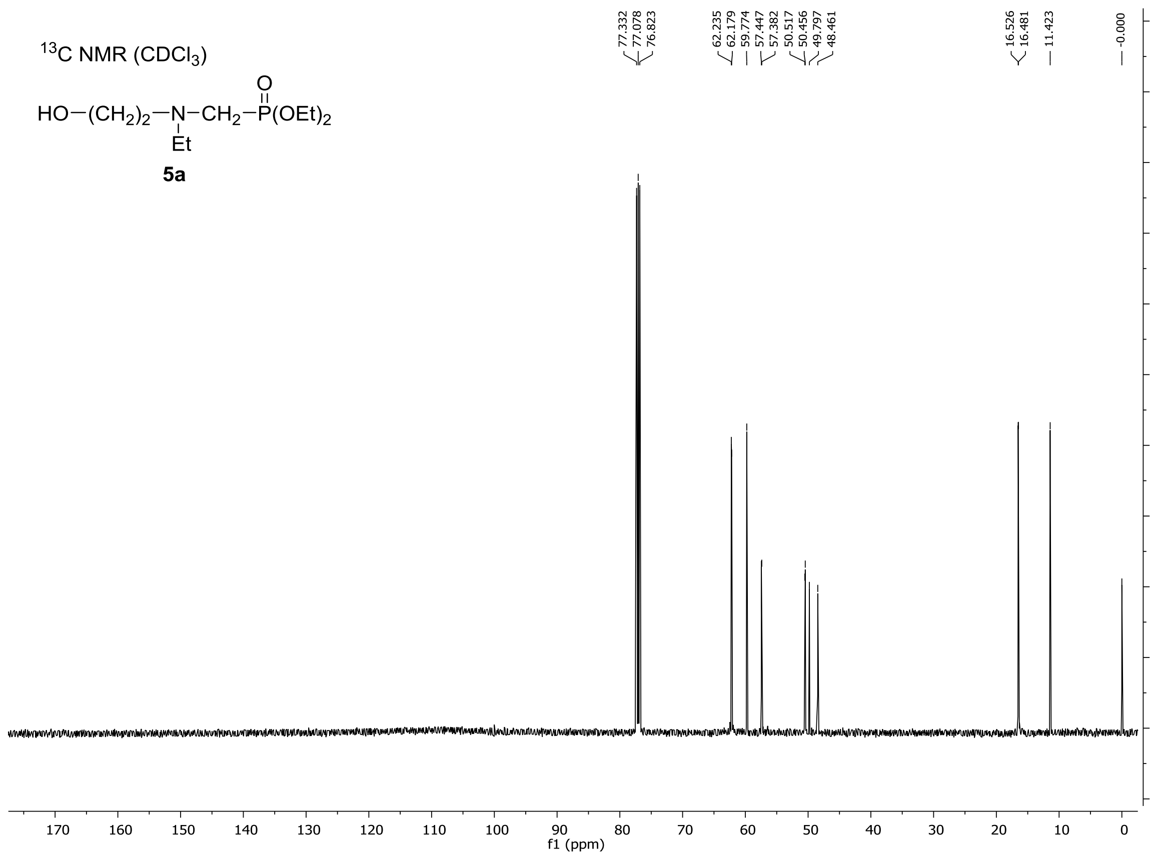
13C NMR (CDCl3)
HO−(CH2)2−N−CH2−P(O)(OEt)2
Et
5a
77.332
77.078
76.823
62.235
62.179
59.774
57.447
57.382
50.517
50.456
49.797
48.461
16.526
16.481
11.423
-0.000
f1 (ppm)

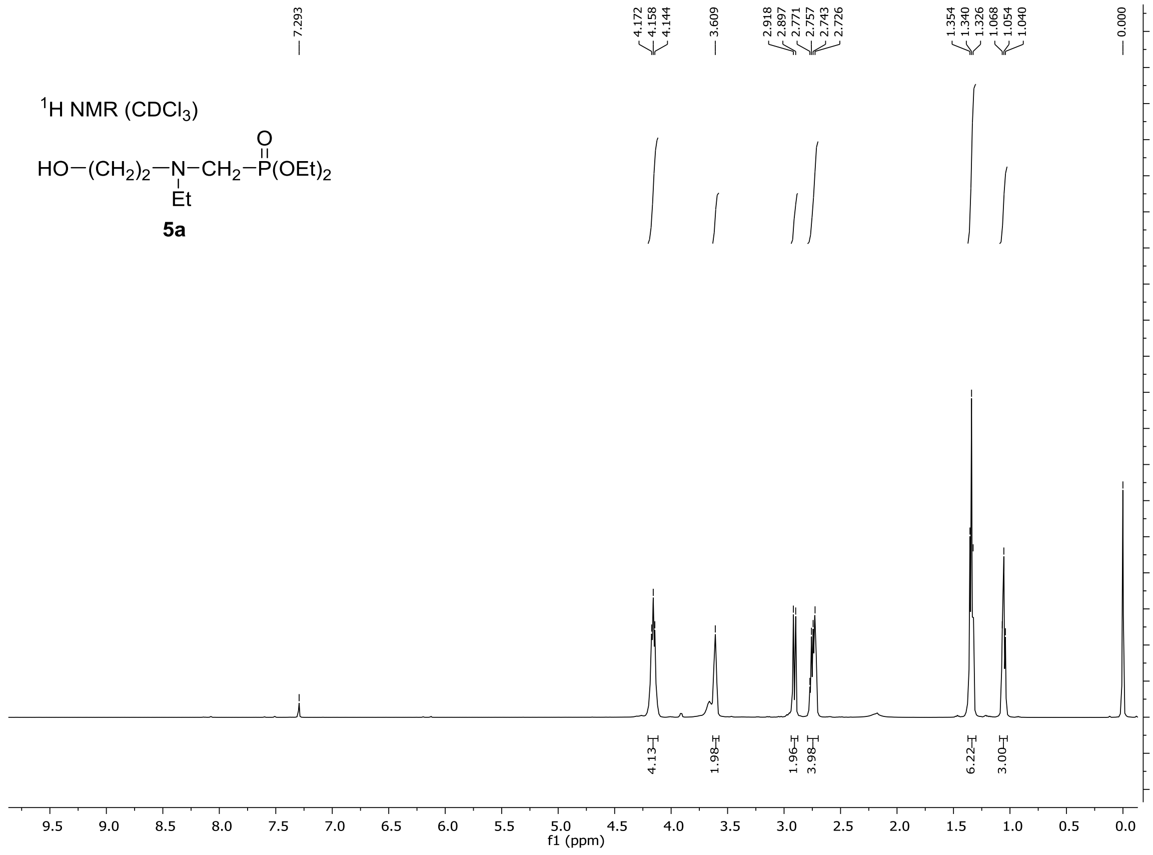
1H NMR (CDCl3)
HO−(CH2)2−N−CH2−P(O)(OEt)2
Et
5a
7.293
4.172
4.158
4.144
3.609
2.918
2.897
2.771
2.757
2.743
2.726
1.354
1.340
1.326
1.068
1.054
1.040
0.000
4.13
1.98
1.96
3.98
6.22
3.00
f1 (ppm)

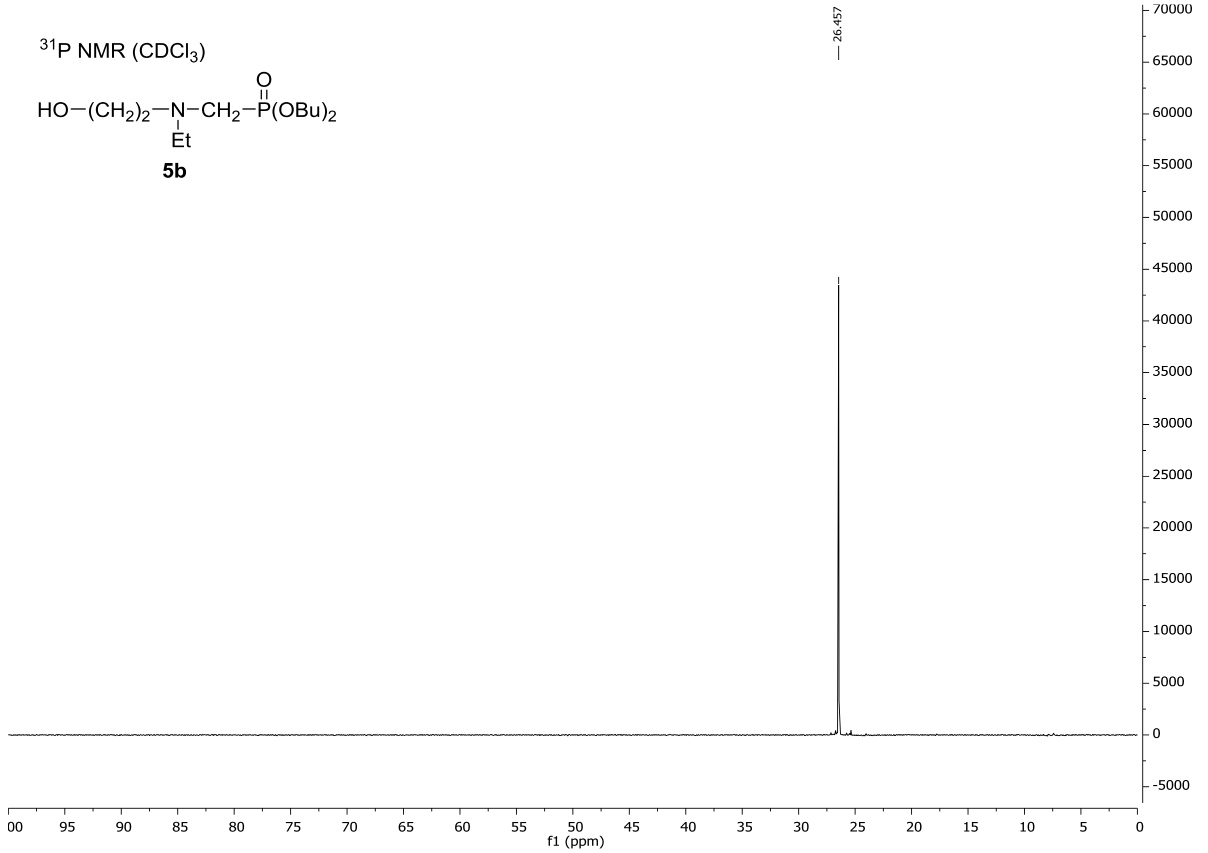

^{31}P NMR (CDCl$_3$)
HO−(CH$_2$)$_2$−N(Et)−CH$_2$−P(O)(OBu)$_2$
5b
26.457
f1 (ppm)

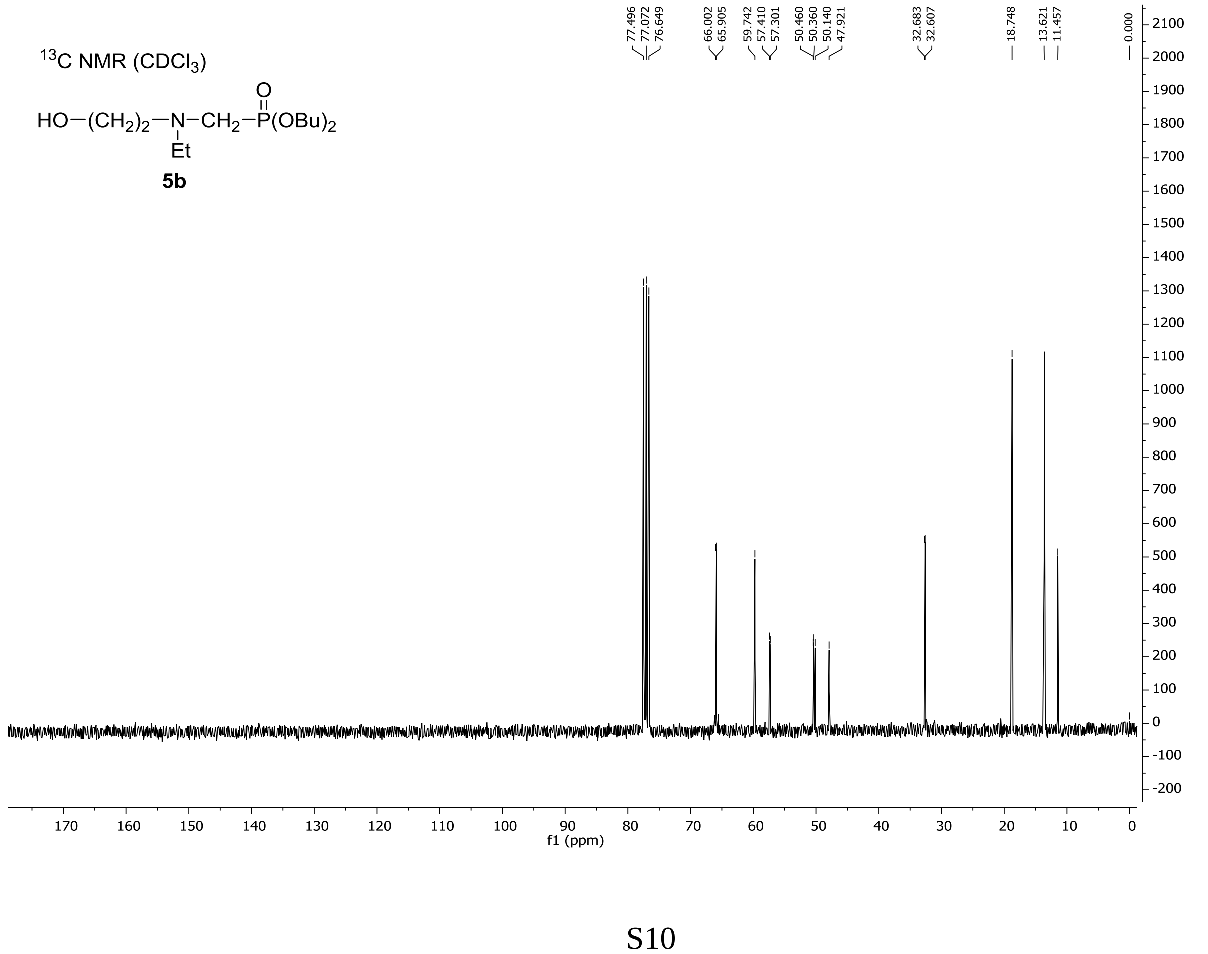

^{13}C NMR (CDCl$_3$)
HO−(CH$_2$)$_2$−N(Et)−CH$_2$−P(O)(OBu)$_2$
5b
77.496
77.072
76.649
66.002
65.905
59.742
57.410
57.301
50.460
50.360
50.140
47.921
32.683
32.607
18.748
13.621
11.457
0.000
f1 (ppm)

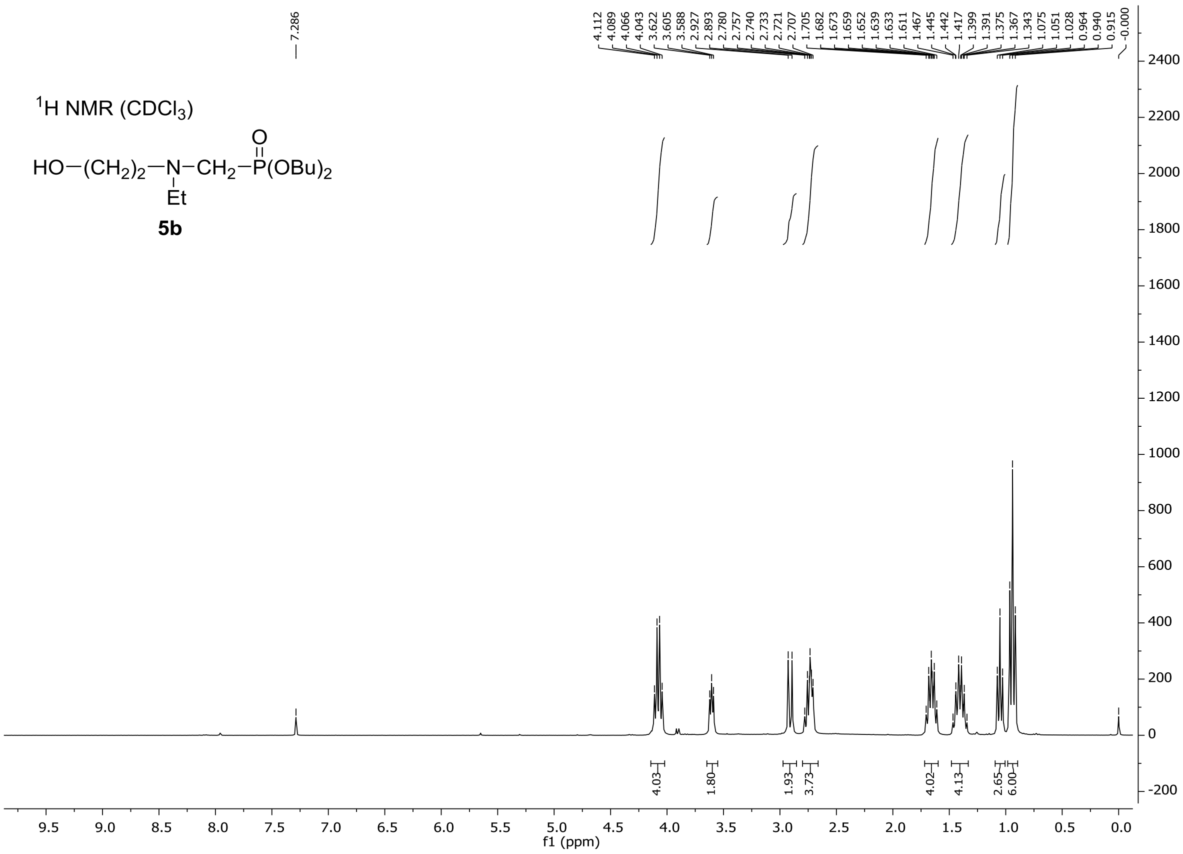
^{1}H NMR ($CDCl_3$)
HO−(CH2)2−N−CH2−P(O)(OBu)2
Et
5b
7.286
4.112 4.089 4.066 4.043 3.622 3.605 3.588 2.927 2.893 2.780 2.757 2.740 2.733 2.721 2.707 1.705 1.682 1.673 1.659 1.652 1.639 1.633 1.611 1.467 1.445 1.442 1.417 1.399 1.391 1.375 1.367 1.343 1.075 1.051 1.028 0.964 0.940 0.915 -0.000
4.03 1.80 1.93 3.73 4.02 4.13 2.65 6.00
f1 (ppm)

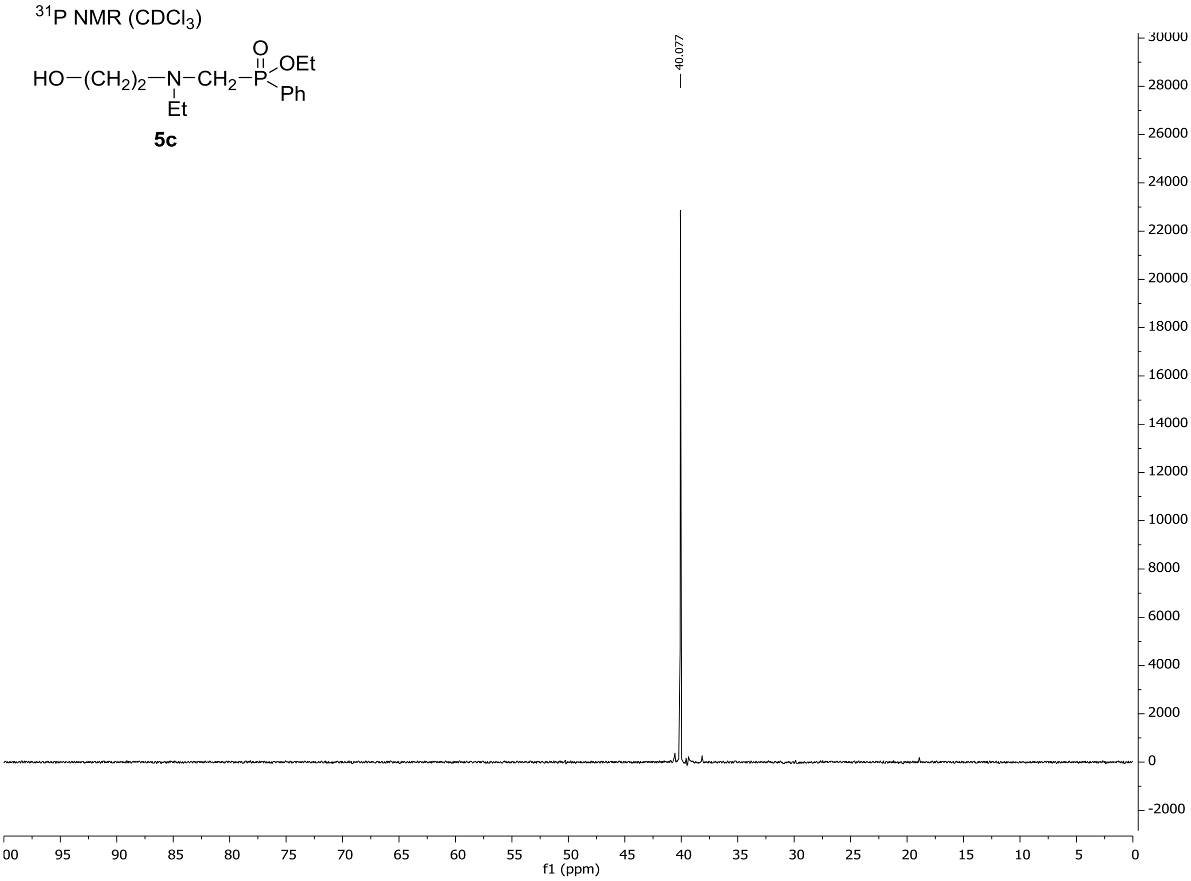
^{31}P NMR ($CDCl_3$)
HO−(CH2)2−N−CH2−P(O)(OEt)Ph
Et
5c
40.077
f1 (ppm)

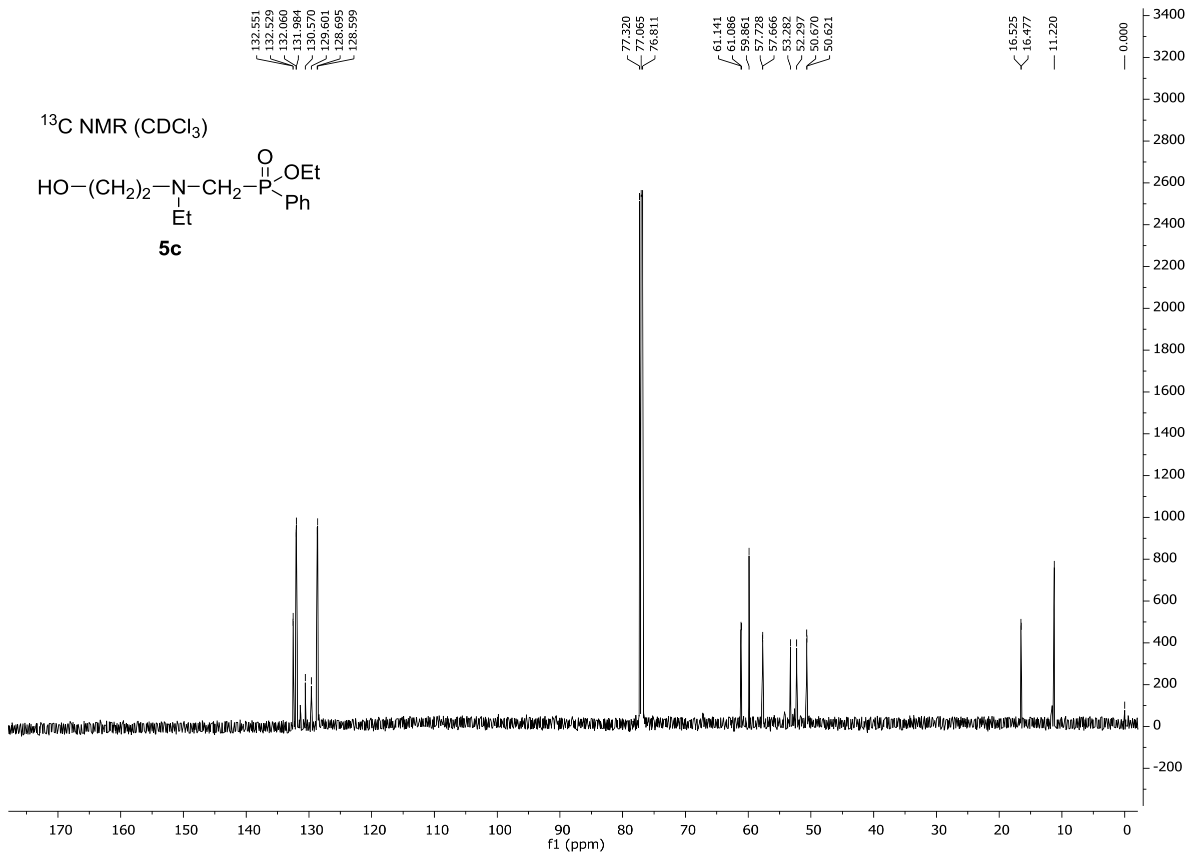
132.551
132.529
132.060
131.984
130.570
129.601
128.695
128.599
77.320
77.065
76.811
61.141
61.086
59.861
57.728
57.666
53.282
52.297
50.670
50.621
16.525
16.477
11.220
0.000
13C NMR (CDCl3)
HO−(CH2)2−N−CH2−P(=O)(OEt)Ph
Et
5c
f1 (ppm)

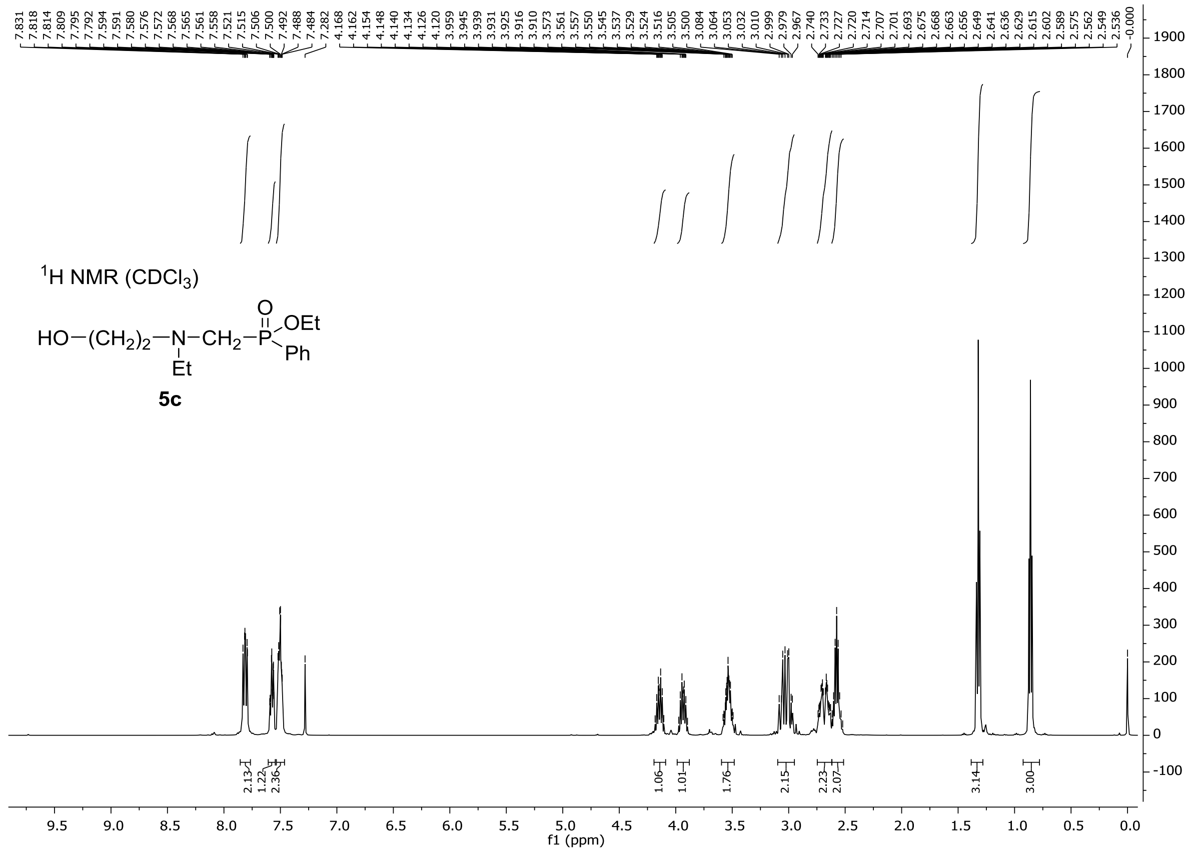
7.831
7.818
7.814
7.809
7.795
7.792
7.594
7.591
7.580
7.576
7.572
7.568
7.565
7.561
7.558
7.521
7.515
7.506
7.500
7.492
7.488
7.484
7.282
4.168
4.162
4.154
4.148
4.140
4.134
4.126
4.120
3.959
3.945
3.939
3.931
3.925
3.916
3.910
3.573
3.561
3.557
3.550
3.545
3.537
3.529
3.524
3.516
3.505
3.500
3.084
3.064
3.053
3.032
3.010
2.999
2.979
2.967
2.740
2.733
2.727
2.720
2.714
2.707
2.701
2.693
2.675
2.668
2.663
2.656
2.649
2.641
2.636
2.629
2.615
2.602
2.589
2.575
2.562
2.549
2.536
-0.000
1H NMR (CDCl3)
HO−(CH2)2−N−CH2−P(=O)(OEt)Ph
Et
5c
2.13
1.22
2.36
1.06
1.01
1.76
2.15
2.23
2.07
3.14
3.00
f1 (ppm)

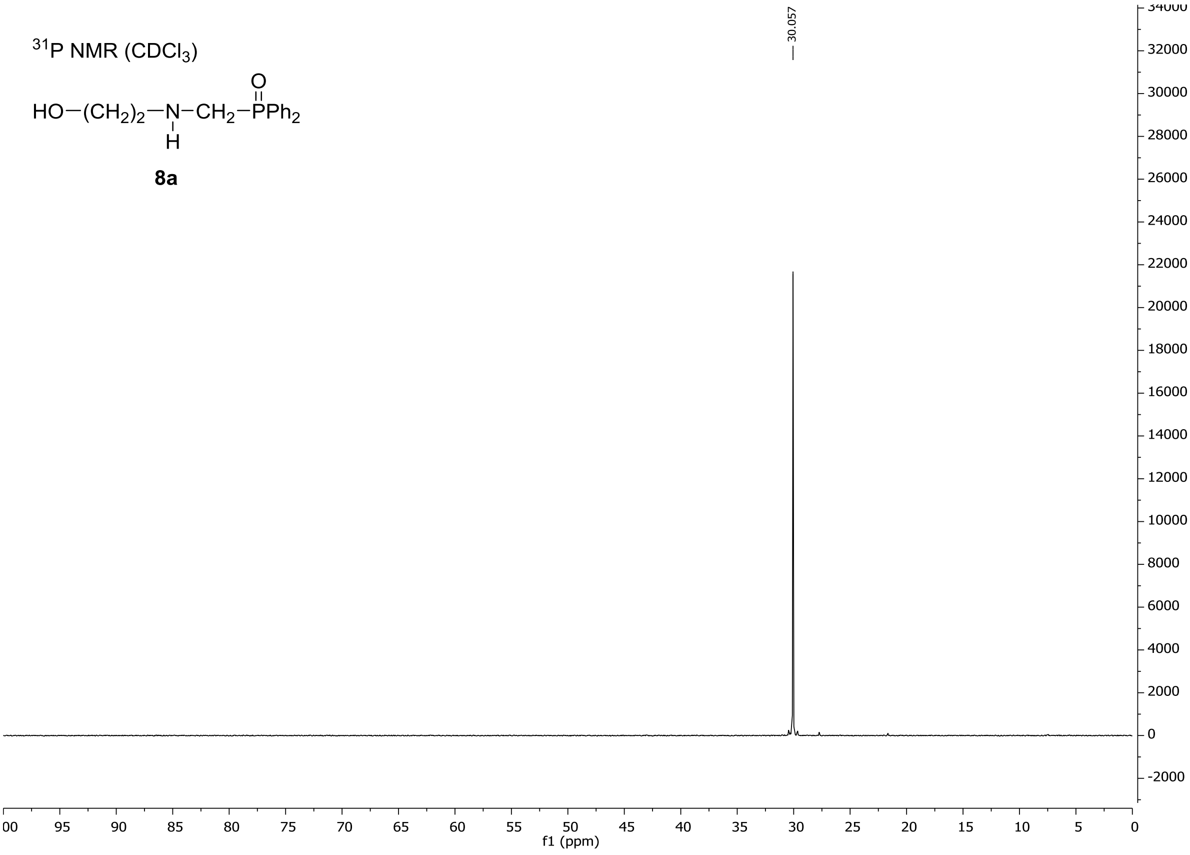
31P NMR (CDCl3)
HO−(CH2)2−N(H)−CH2−P(O)Ph2
8a
30.057
f1 (ppm)

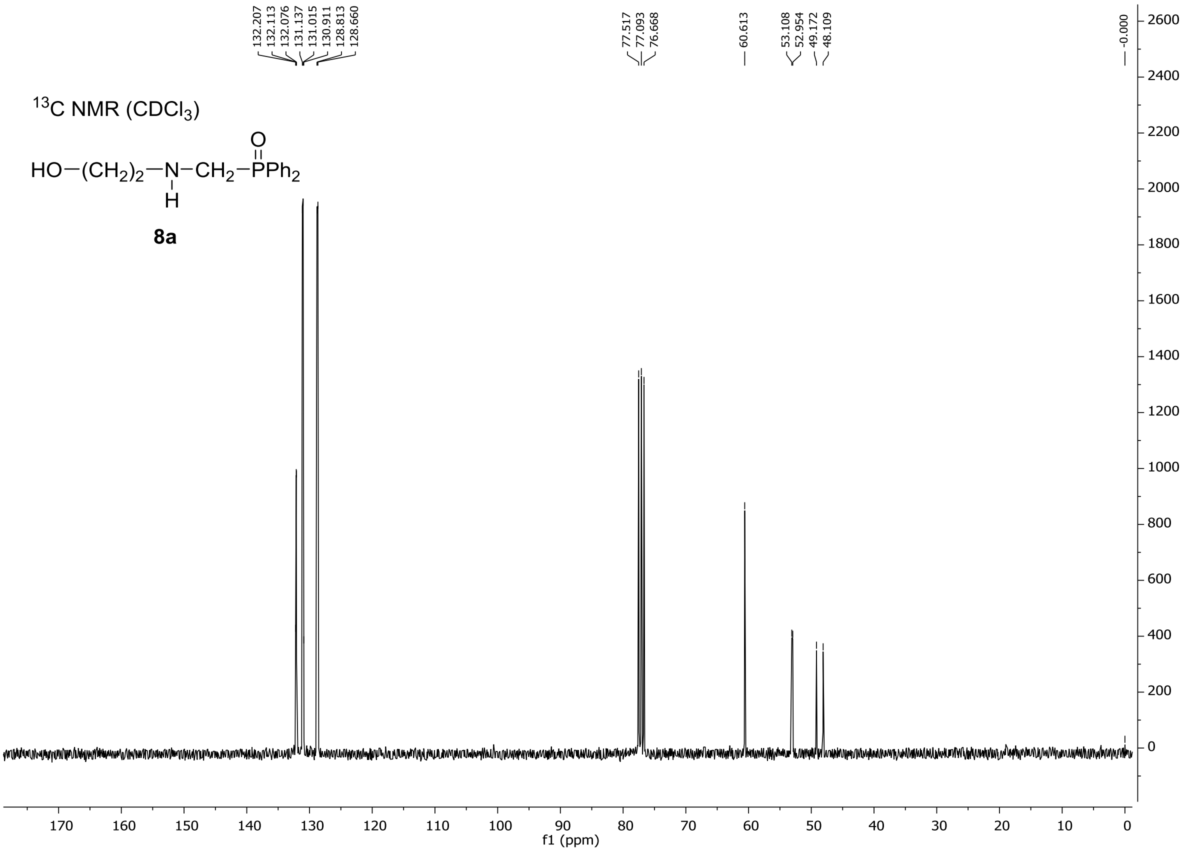
13C NMR (CDCl3)
HO−(CH2)2−N(H)−CH2−P(O)Ph2
8a
132.207
132.113
132.076
131.137
131.015
130.911
128.813
128.660
77.517
77.093
76.668
60.613
53.108
52.954
49.172
48.109
-0.000
f1 (ppm)

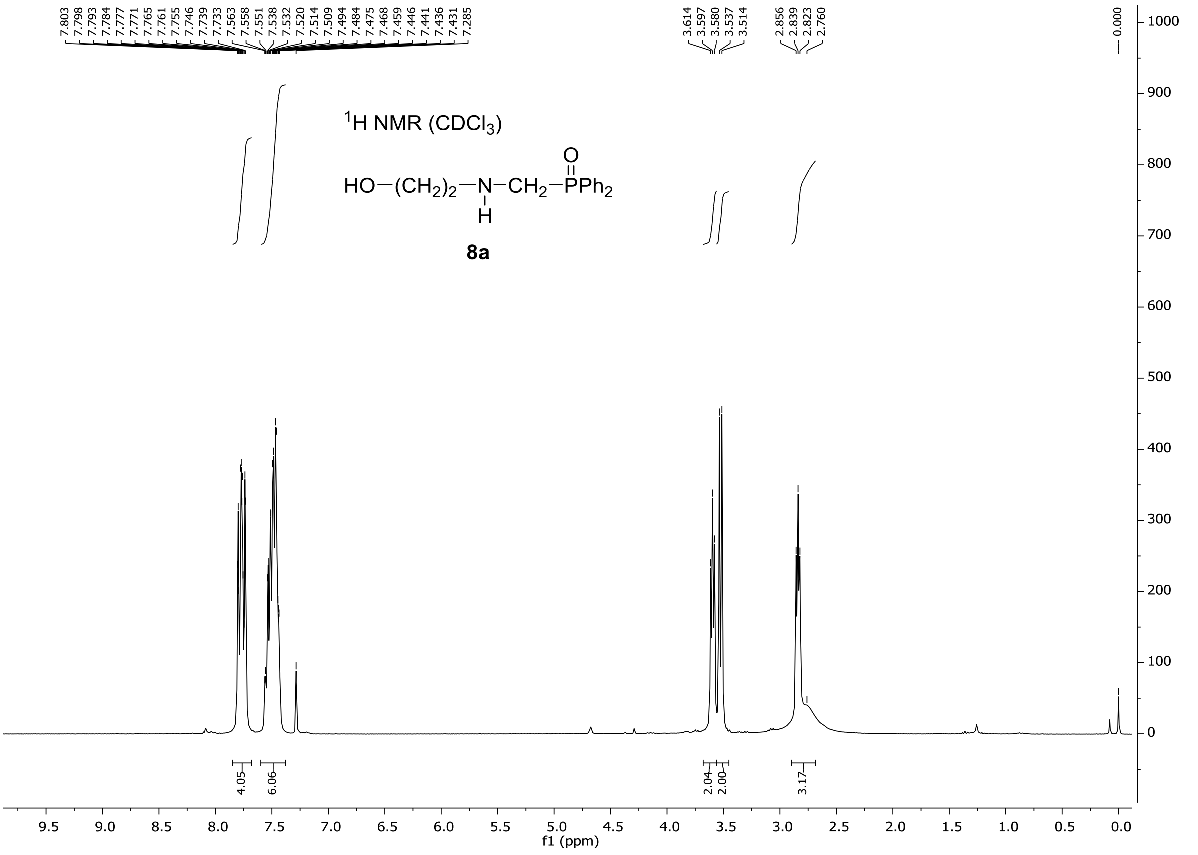
7.803 7.798 7.793 7.784 7.777 7.771 7.765 7.761 7.755 7.746 7.739 7.733 7.563 7.558 7.551 7.538 7.532 7.520 7.514 7.509 7.494 7.484 7.475 7.468 7.459 7.446 7.441 7.436 7.431 7.285
3.614 3.597 3.580 3.537 3.514
2.856 2.839 2.823 2.760
0.000
1H NMR (CDCl3)
HO−(CH2)2−N−CH2−PPh2
O
H
8a
4.05
6.06
2.04
2.00
3.17
f1 (ppm)

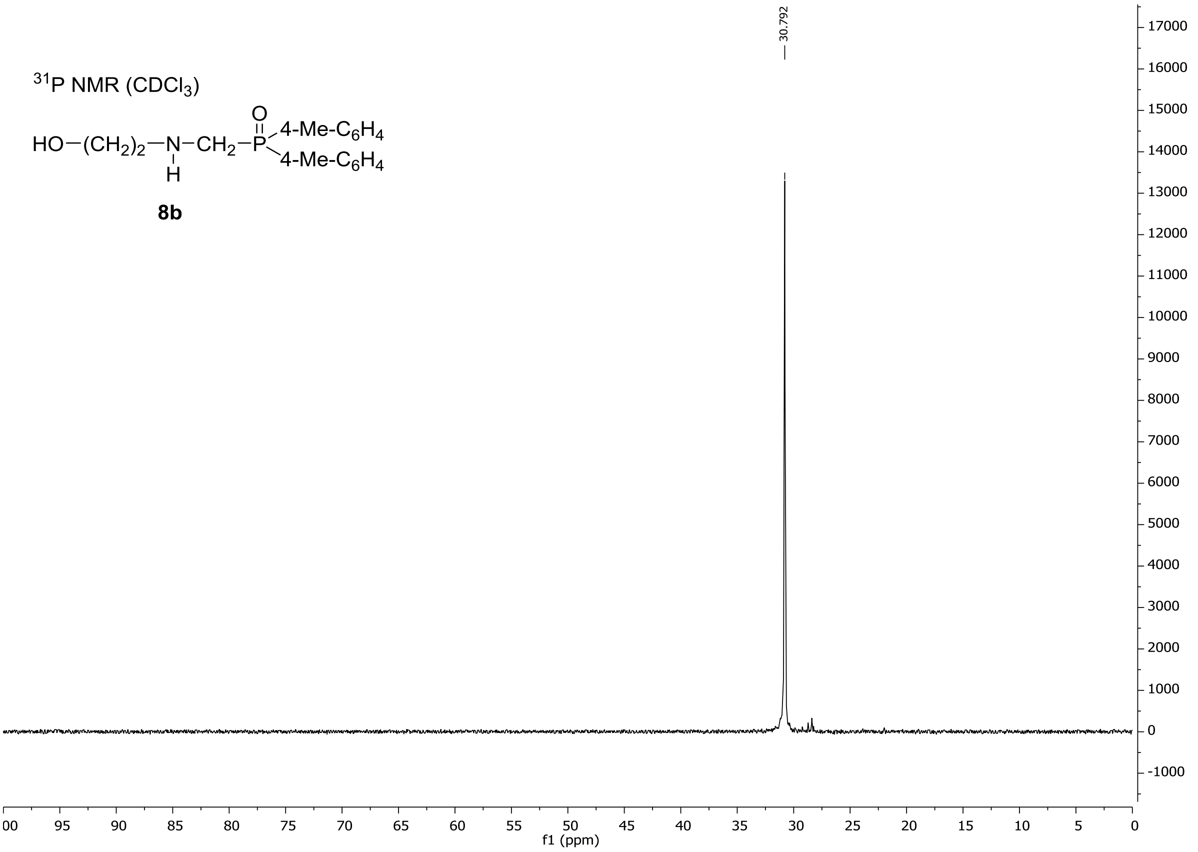
30.792
31P NMR (CDCl3)
HO−(CH2)2−N−CH2−P
O
H
4-Me-C6H4
4-Me-C6H4
8b
f1 (ppm)

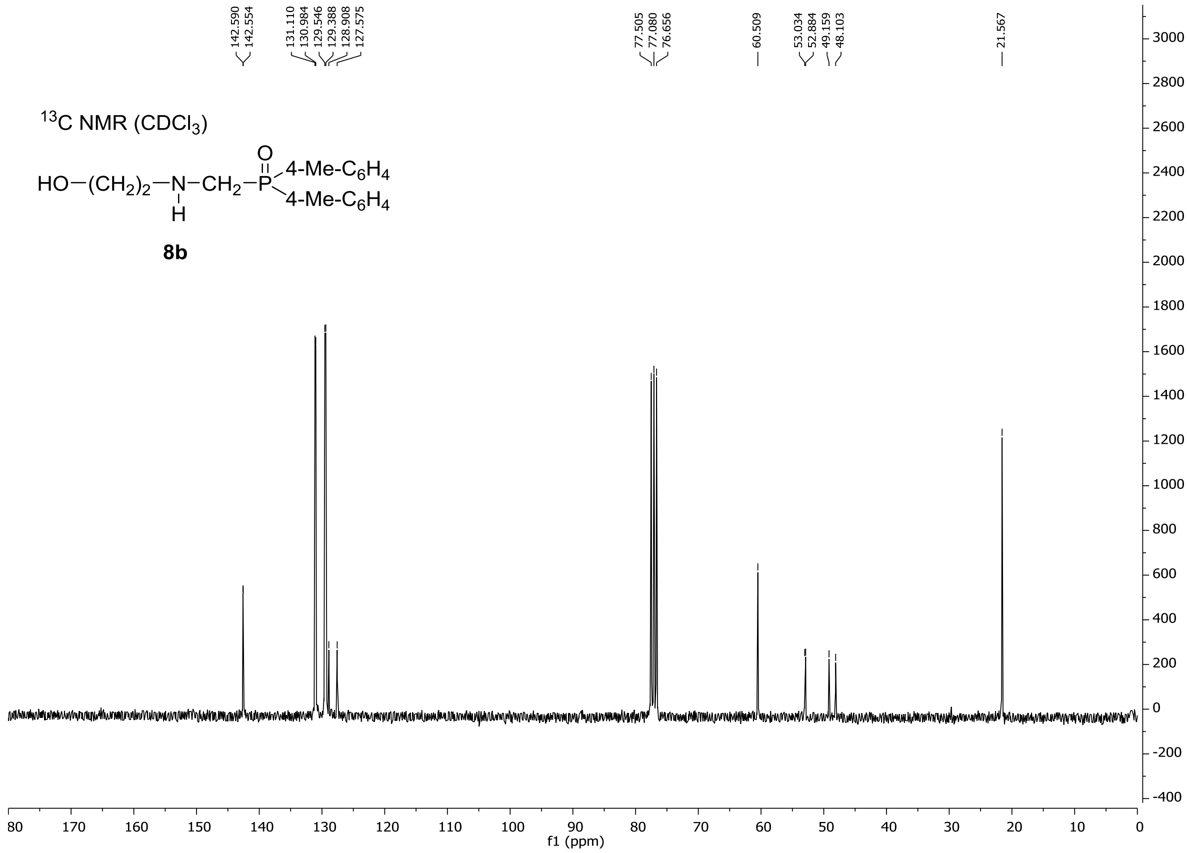

142.590
142.554
131.110
130.984
129.546
129.388
128.908
127.575
77.505
77.080
76.656
60.509
53.034
52.884
49.159
48.103
21.567
13C NMR (CDCl3)
HO−(CH2)2−N−CH2−P 4-Me-C6H4 4-Me-C6H4
O
H
8b
f1 (ppm)

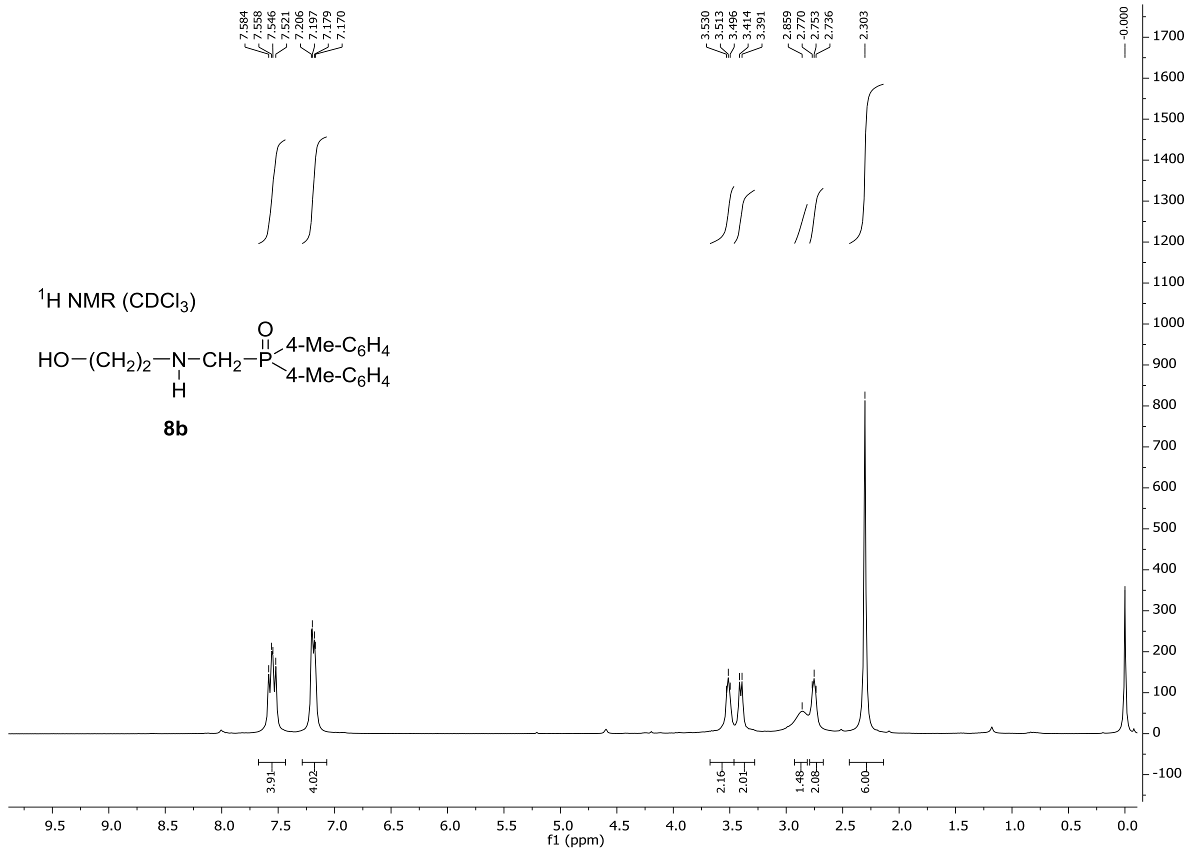

7.584
7.558
7.546
7.521
7.206
7.197
7.179
7.170
3.530
3.513
3.496
3.414
3.391
2.859
2.770
2.753
2.736
2.303
-0.000
1H NMR (CDCl3)
HO−(CH2)2−N−CH2−P 4-Me-C6H4 4-Me-C6H4
O
H
8b
3.91
4.02
2.16
2.01
1.48
2.08
6.00
f1 (ppm)

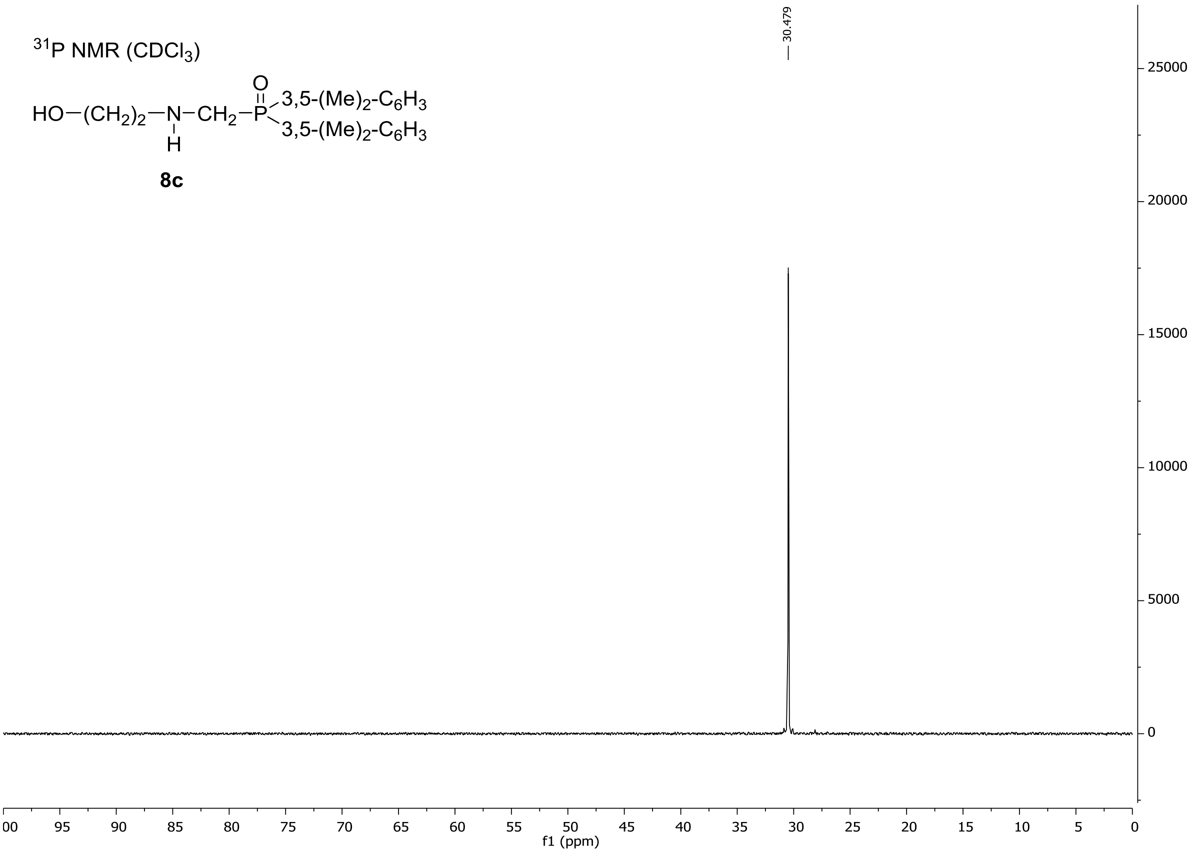
30.479
^{31}P NMR ($CDCl_3$)
HO−(CH_2)$_2$−N(H)−CH_2−P(=O)(3,5-(Me)$_2$-C_6H_3)$_2$
8c
f1 (ppm)

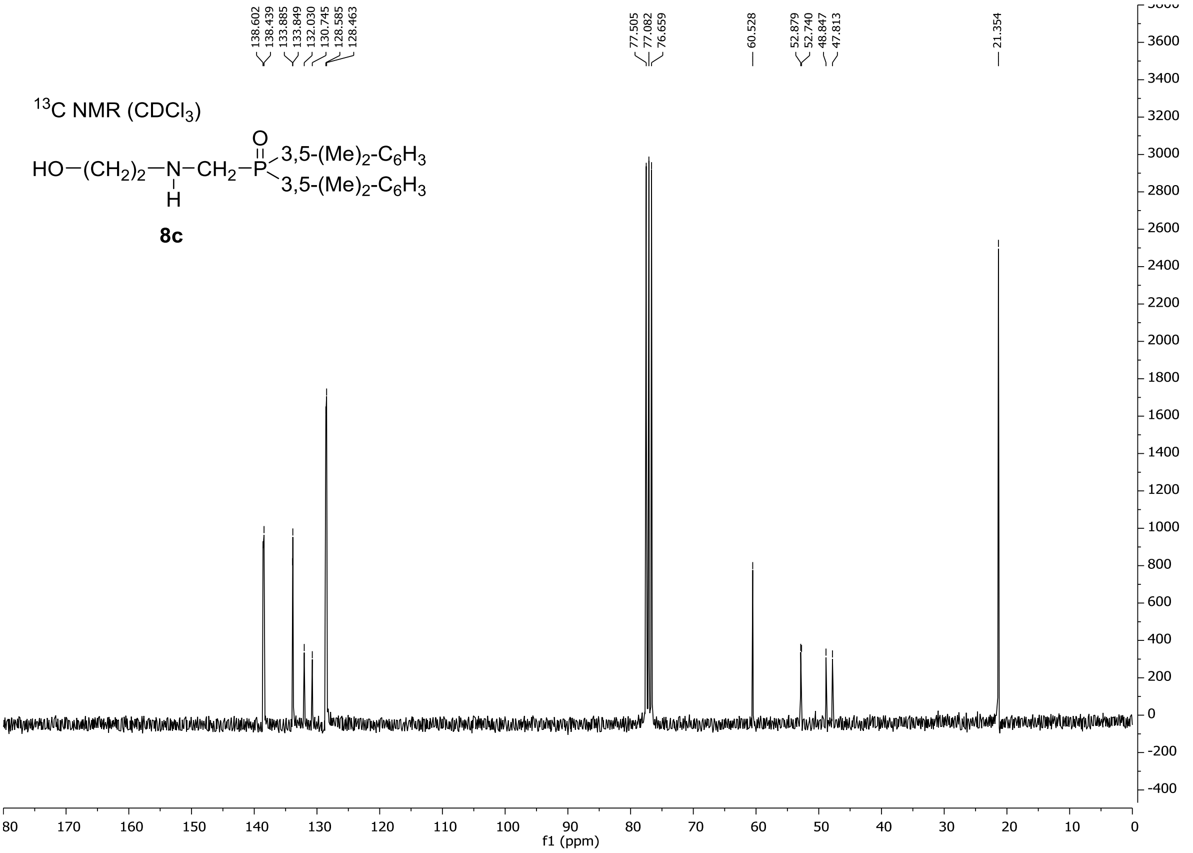
138.602
138.439
133.885
133.849
132.030
130.745
128.585
128.463
77.505
77.082
76.659
60.528
52.879
52.740
48.847
47.813
21.354
^{13}C NMR ($CDCl_3$)
HO−(CH_2)$_2$−N(H)−CH_2−P(=O)(3,5-(Me)$_2$-C_6H_3)$_2$
8c
f1 (ppm)

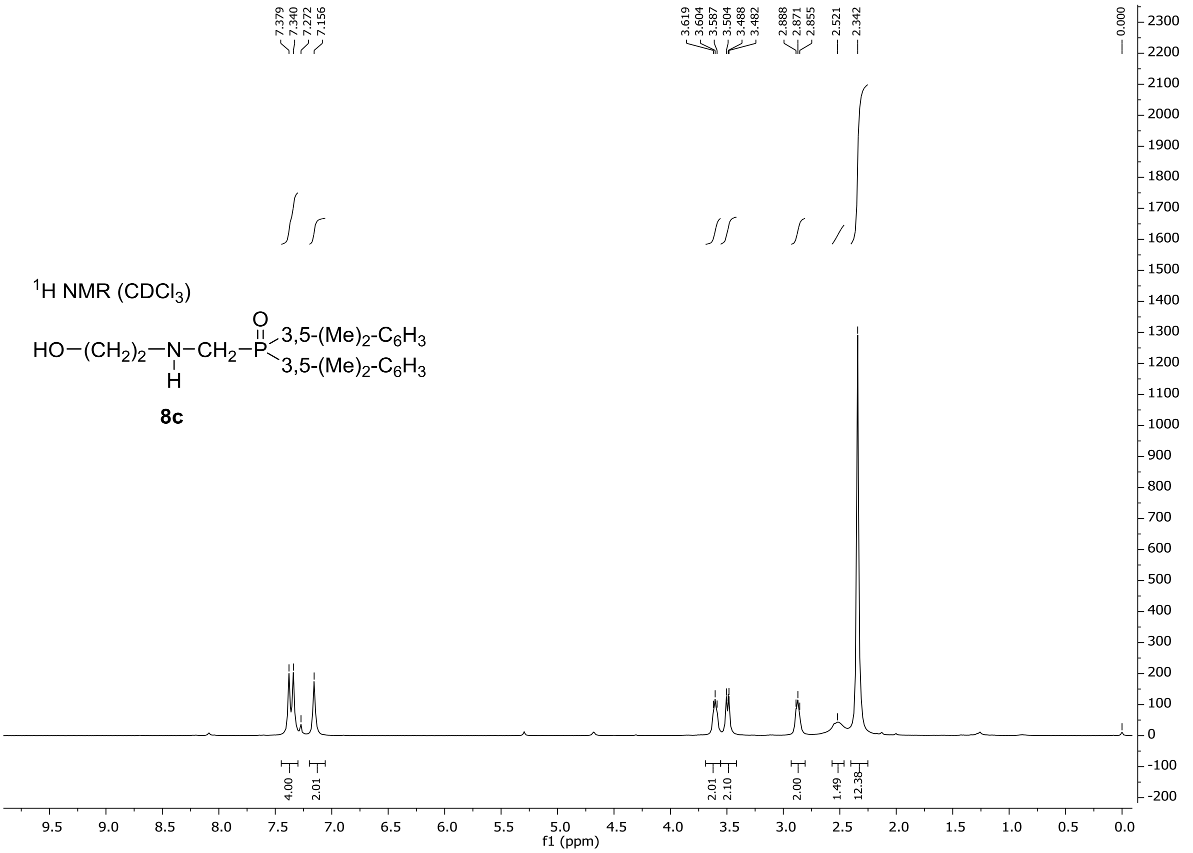
1H NMR (CDCl3)
HO−(CH2)2−N−CH2−P(O)(3,5-(Me)2-C6H3)2
H
8c
7.379
7.340
7.272
7.156
3.619
3.604
3.587
3.504
3.488
3.482
2.888
2.871
2.855
2.521
2.342
0.000
4.00
2.01
2.01
2.10
2.00
1.49
12.38
f1 (ppm)

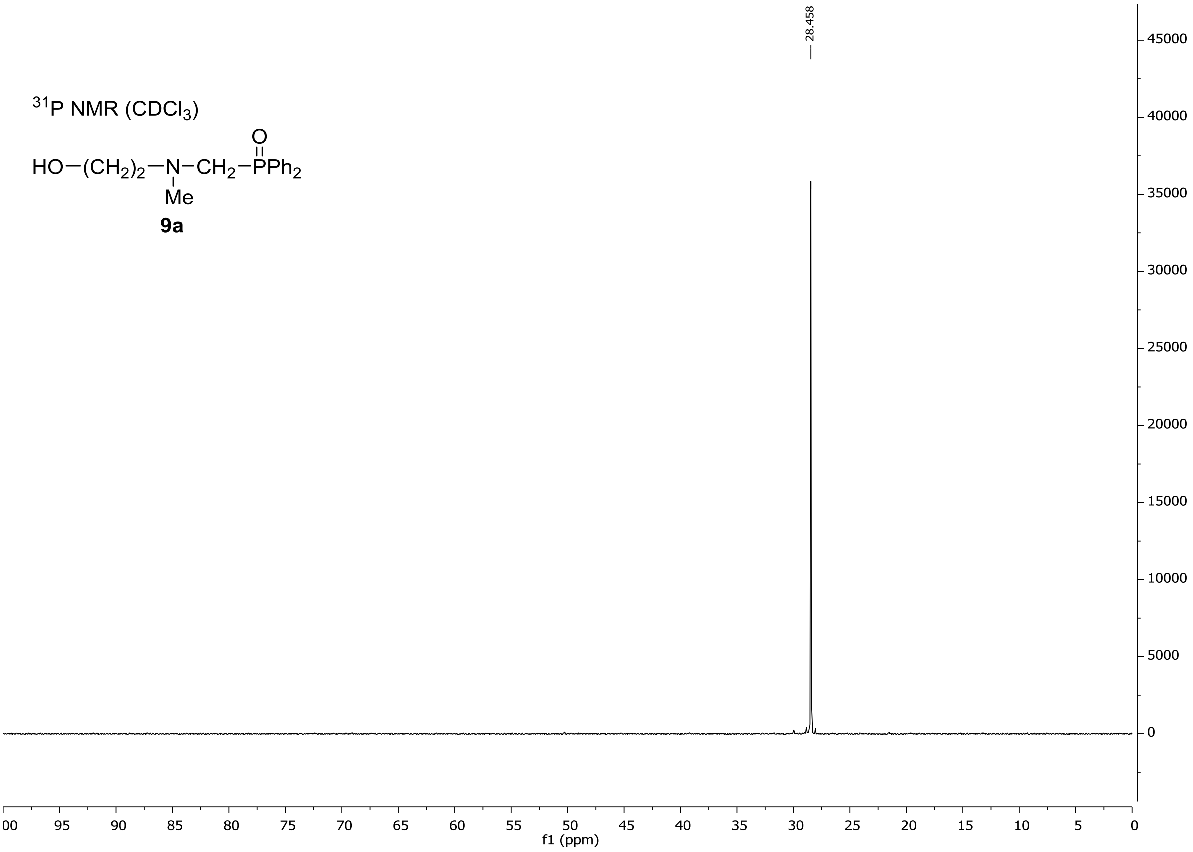
31P NMR (CDCl3)
HO−(CH2)2−N−CH2−P(O)Ph2
Me
9a
28.458
f1 (ppm)

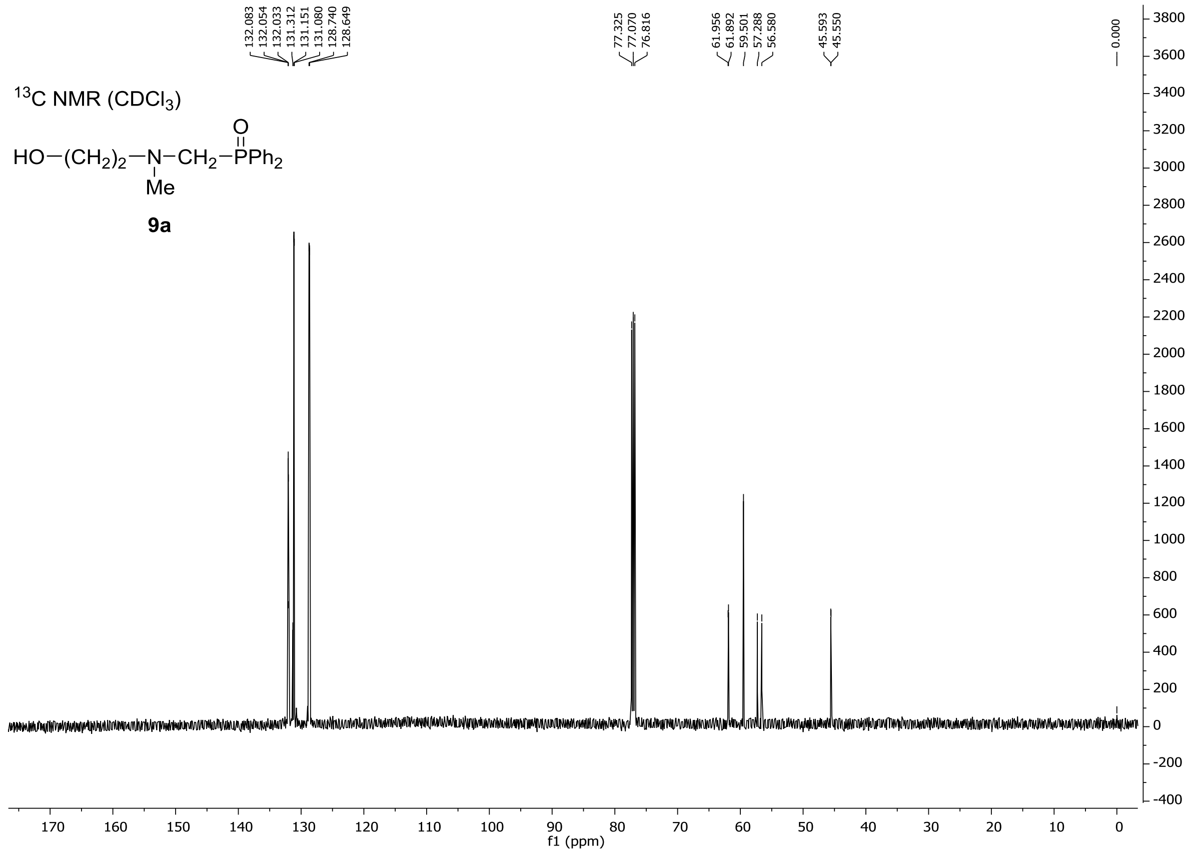
132.083
132.054
132.033
131.312
131.151
131.080
128.740
128.649
77.325
77.070
76.816
61.956
61.892
59.501
57.288
56.580
45.593
45.550
0.000
13C NMR (CDCl3)
HO−(CH2)2−N−CH2−P(O)Ph2
Me
9a
f1 (ppm)

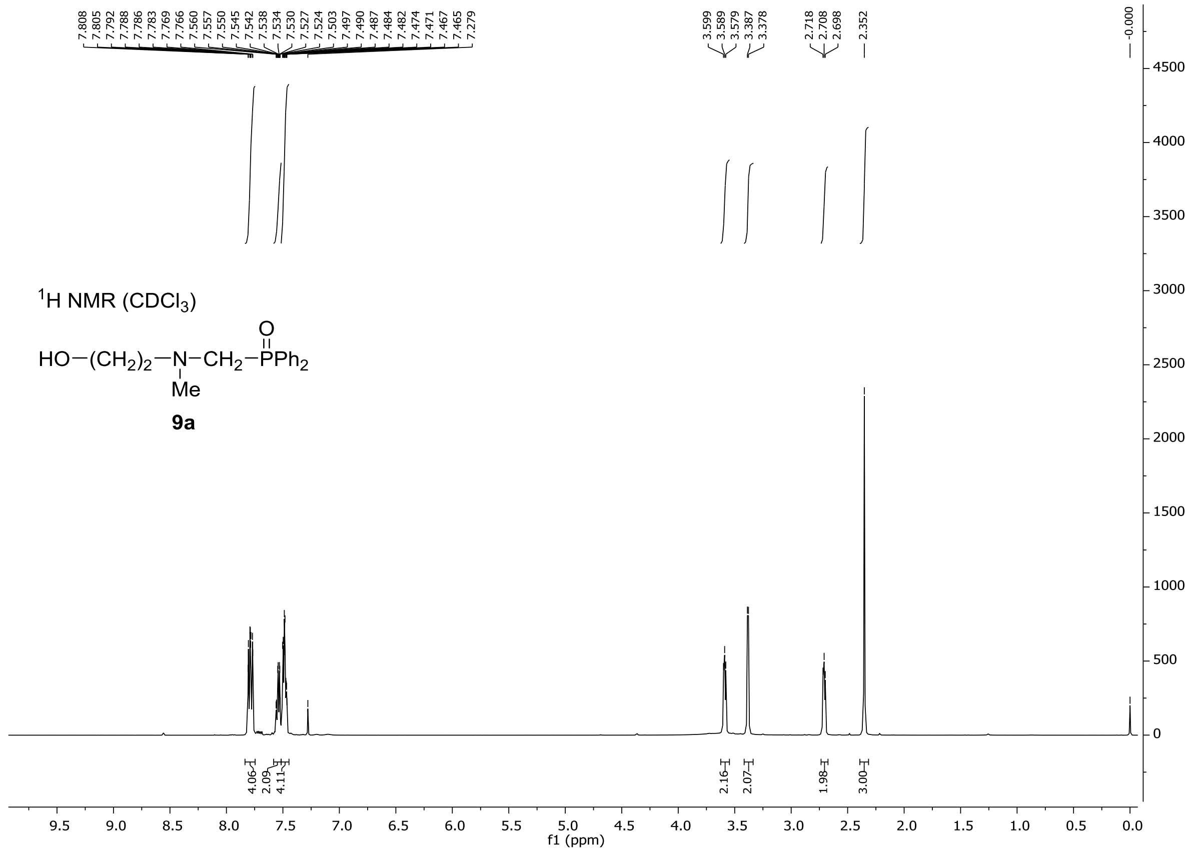
7.808
7.805
7.792
7.788
7.786
7.783
7.769
7.766
7.560
7.557
7.550
7.545
7.542
7.538
7.534
7.530
7.527
7.524
7.503
7.497
7.490
7.487
7.484
7.482
7.474
7.471
7.467
7.465
7.279
3.599
3.589
3.579
3.387
3.378
2.718
2.708
2.698
2.352
-0.000
1H NMR (CDCl3)
HO−(CH2)2−N−CH2−P(O)Ph2
Me
9a
4.06
2.09
4.11
2.16
2.07
1.98
3.00
f1 (ppm)

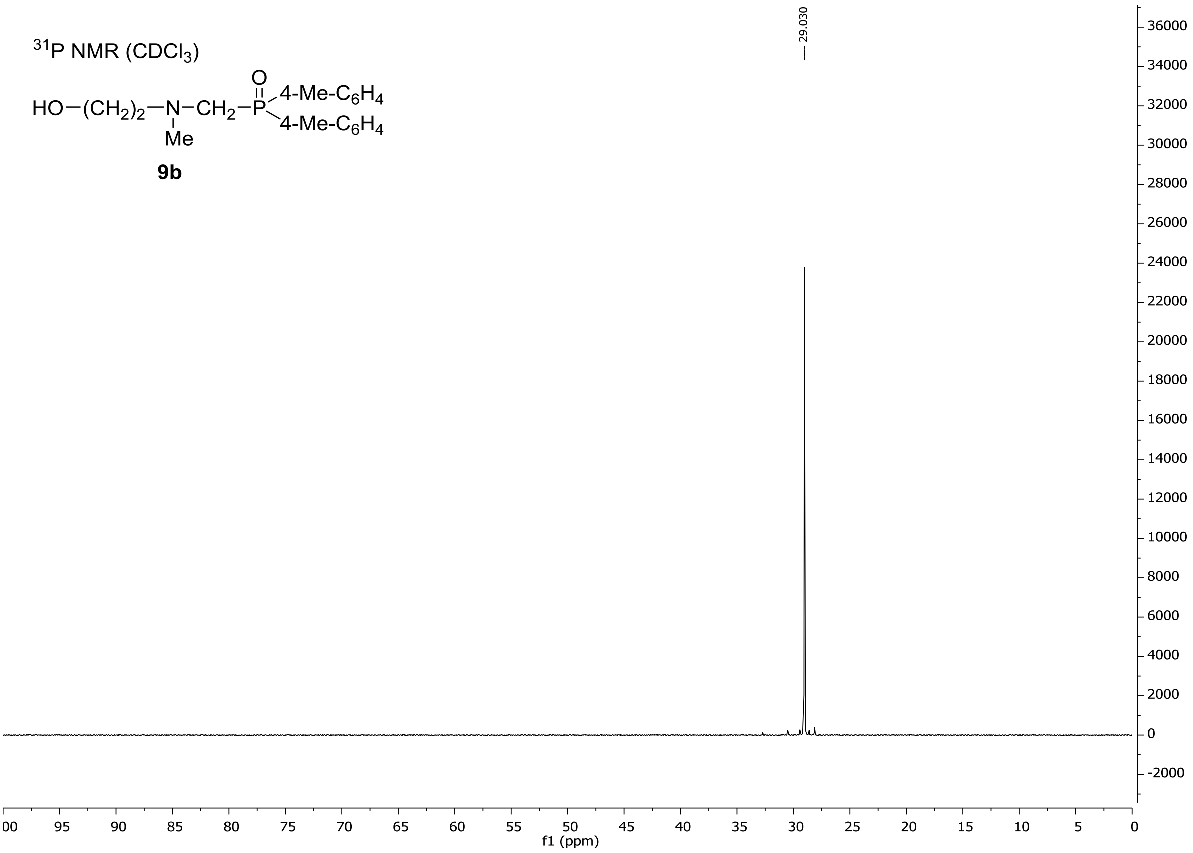

31P NMR (CDCl3)
HO−(CH2)2−N−CH2−P(=O)(4-Me-C6H4)(4-Me-C6H4)
Me
9b
29.030
f1 (ppm)

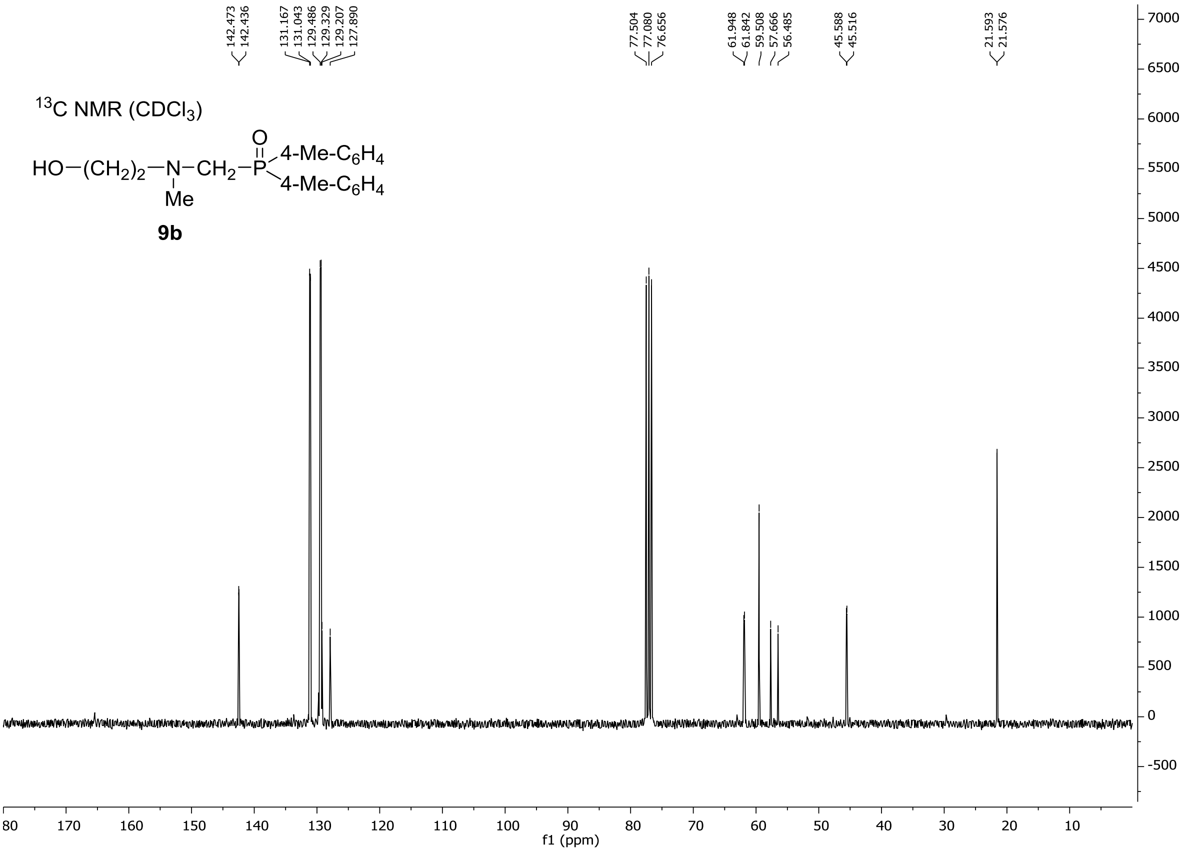

13C NMR (CDCl3)
HO−(CH2)2−N−CH2−P(=O)(4-Me-C6H4)(4-Me-C6H4)
Me
9b
142.473
142.436
131.167
131.043
129.486
129.329
129.207
127.890
77.504
77.080
76.656
61.948
61.842
59.508
57.666
56.485
45.588
45.516
21.593
21.576
f1 (ppm)

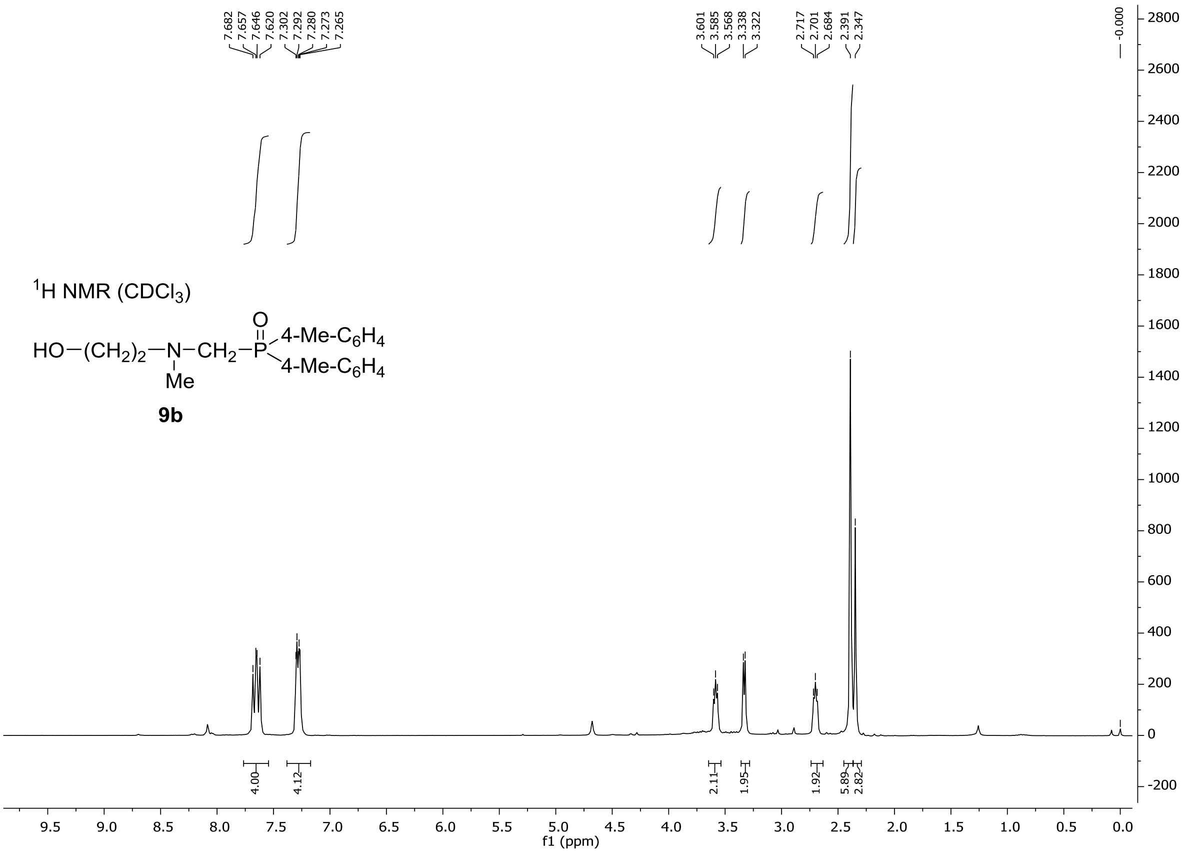
1H NMR (CDCl3)
HO−(CH2)2−N−CH2−P(=O)(4-Me-C6H4)2
Me
9b
7.682 7.657 7.646 7.620 7.302 7.292 7.280 7.273 7.265
3.601 3.585 3.568 3.338 3.322
2.717 2.701 2.684 2.391 2.347
-0.000
4.00 4.12 2.11 1.95 1.92 5.89 2.82
f1 (ppm)

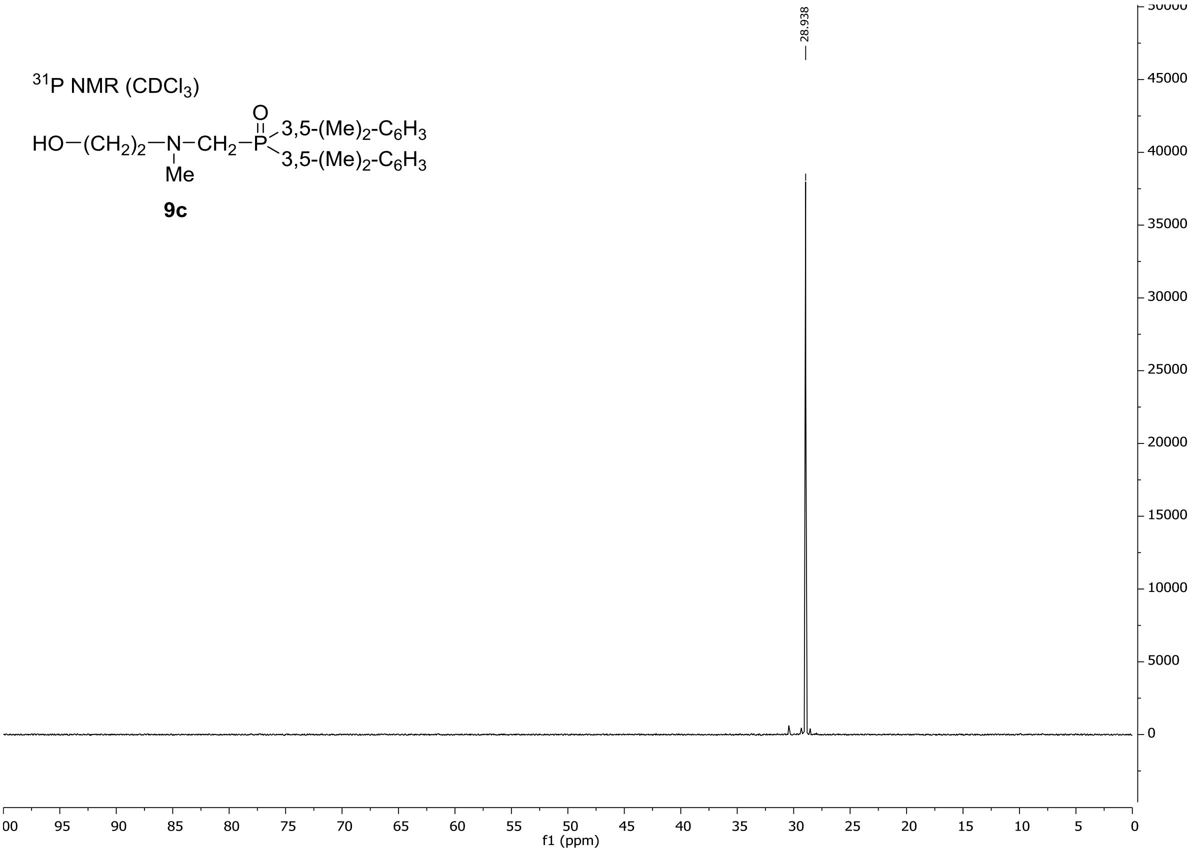
31P NMR (CDCl3)
HO−(CH2)2−N−CH2−P(=O)(3,5-(Me)2-C6H3)2
Me
9c
28.938
f1 (ppm)

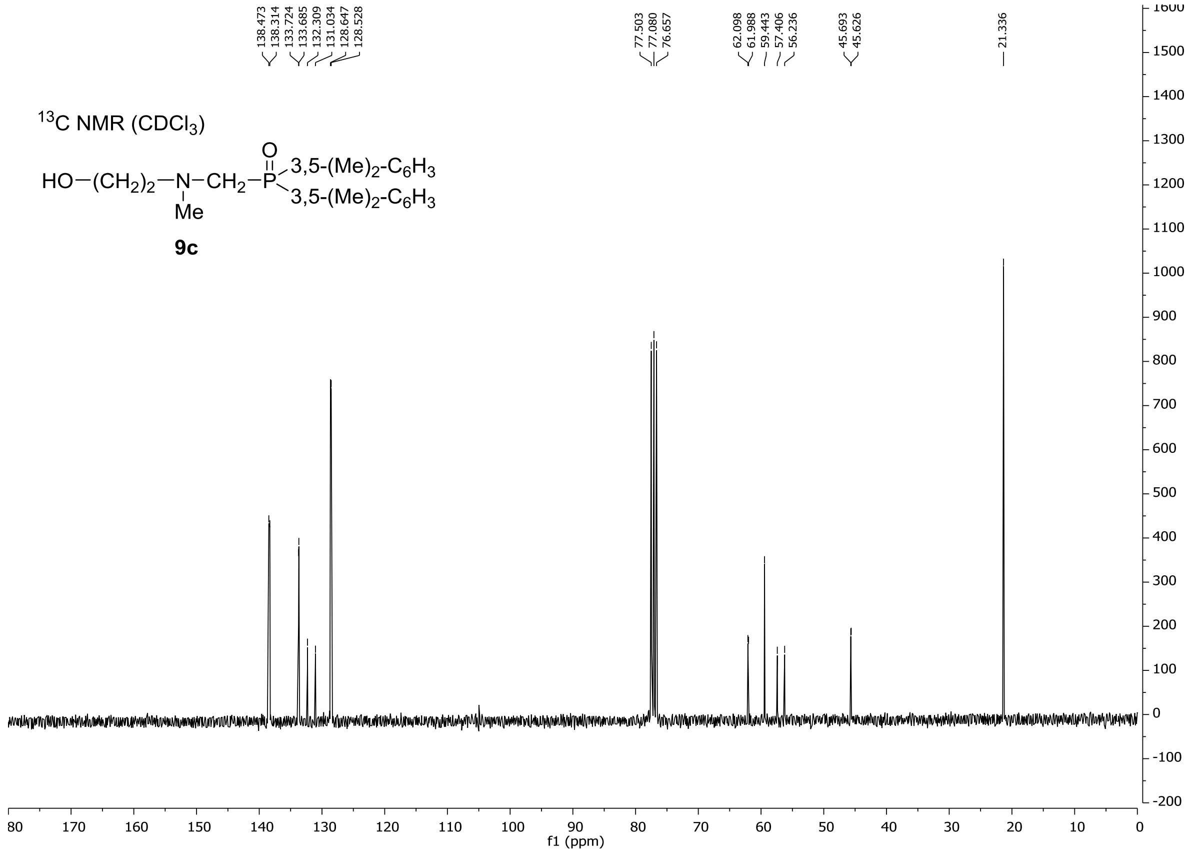
138.473
138.314
133.724
133.685
132.309
131.034
128.647
128.528
77.503
77.080
76.657
62.098
61.988
59.443
57.406
56.236
45.693
45.626
21.336
13C NMR (CDCl3)
HO−(CH2)2−N−CH2−P(O) 3,5-(Me)2-C6H3 3,5-(Me)2-C6H3
Me
9c
f1 (ppm)

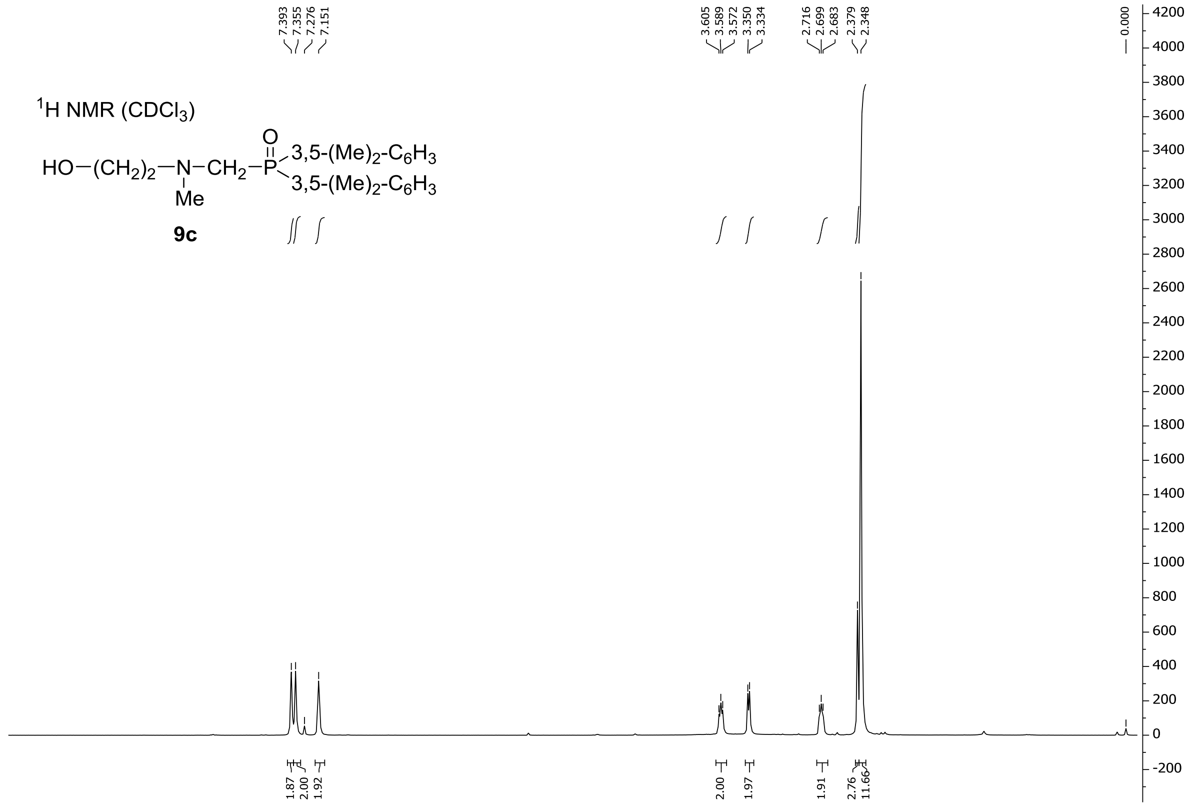
7.393
7.355
7.276
7.151
3.605
3.589
3.572
3.350
3.334
2.716
2.699
2.683
2.379
2.348
0.000
1H NMR (CDCl3)
HO−(CH2)2−N−CH2−P(O) 3,5-(Me)2-C6H3 3,5-(Me)2-C6H3
Me
9c
1.87
2.00
1.92
2.00
1.97
1.91
2.76
11.66
f1 (ppm)

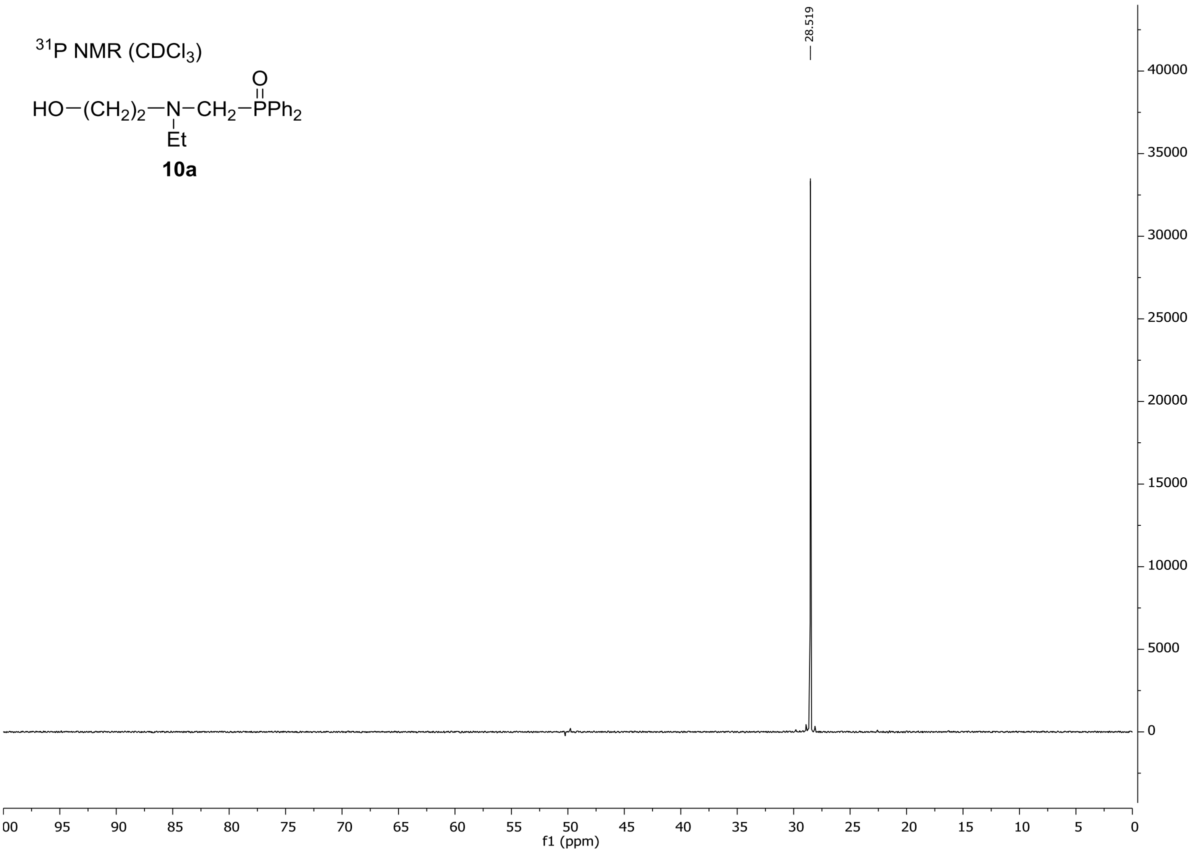

^{31}P NMR (CDCl$_3$)
HO−(CH$_2$)$_2$−N−CH$_2$−P(O)Ph$_2$
Et
10a
28.519
f1 (ppm)

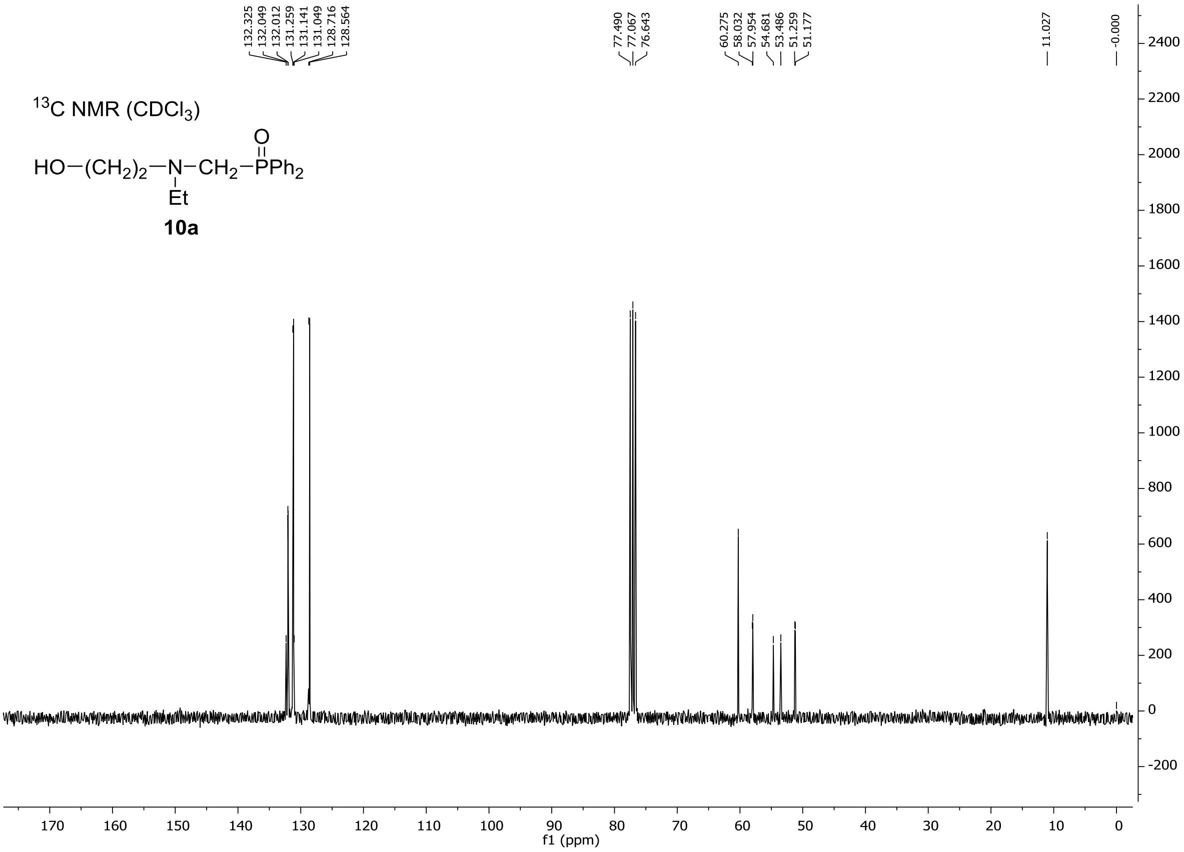

^{13}C NMR (CDCl$_3$)
HO−(CH$_2$)$_2$−N−CH$_2$−P(O)Ph$_2$
Et
10a
132.325
132.049
132.012
131.259
131.141
131.049
128.716
128.564
77.490
77.067
76.643
60.275
58.032
57.954
54.681
53.486
51.259
51.177
11.027
-0.000
f1 (ppm)

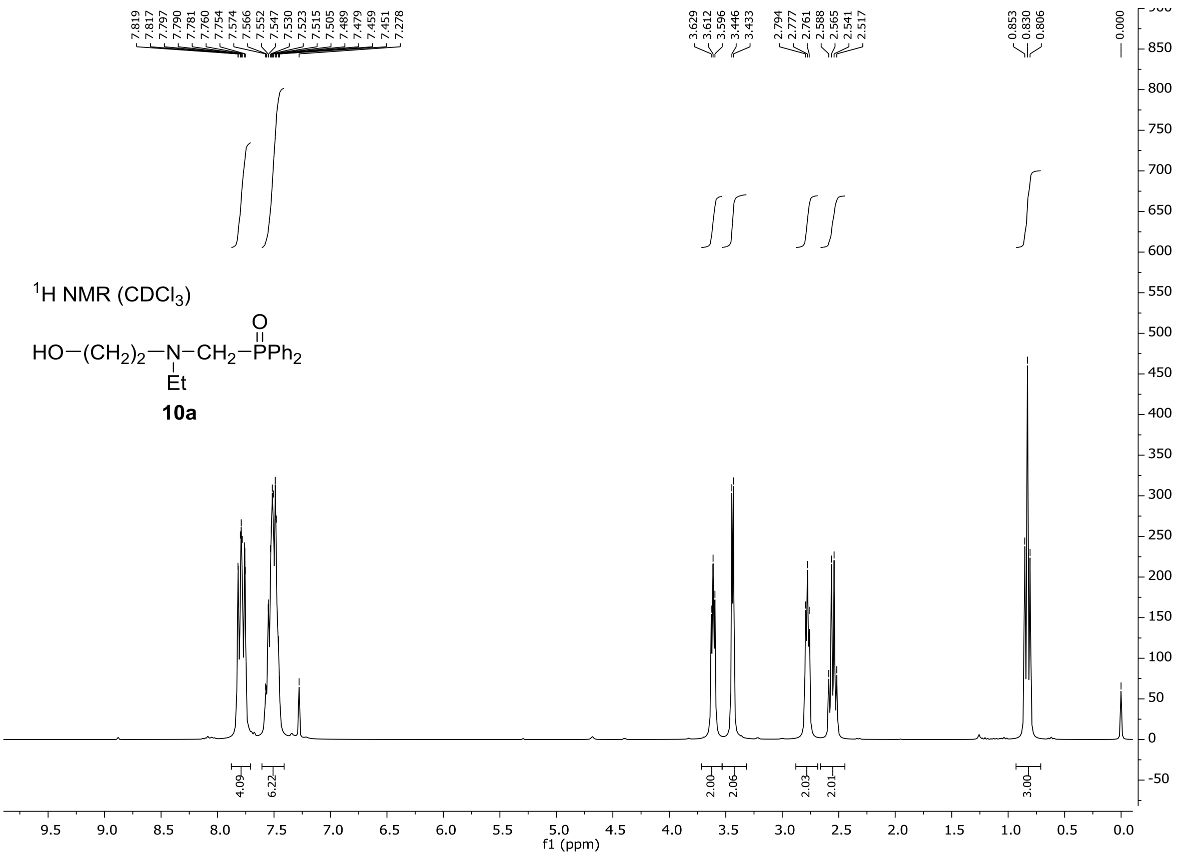

1H NMR (CDCl3)
HO−(CH2)2−N−CH2−P(O)Ph2
Et
10a
f1 (ppm)

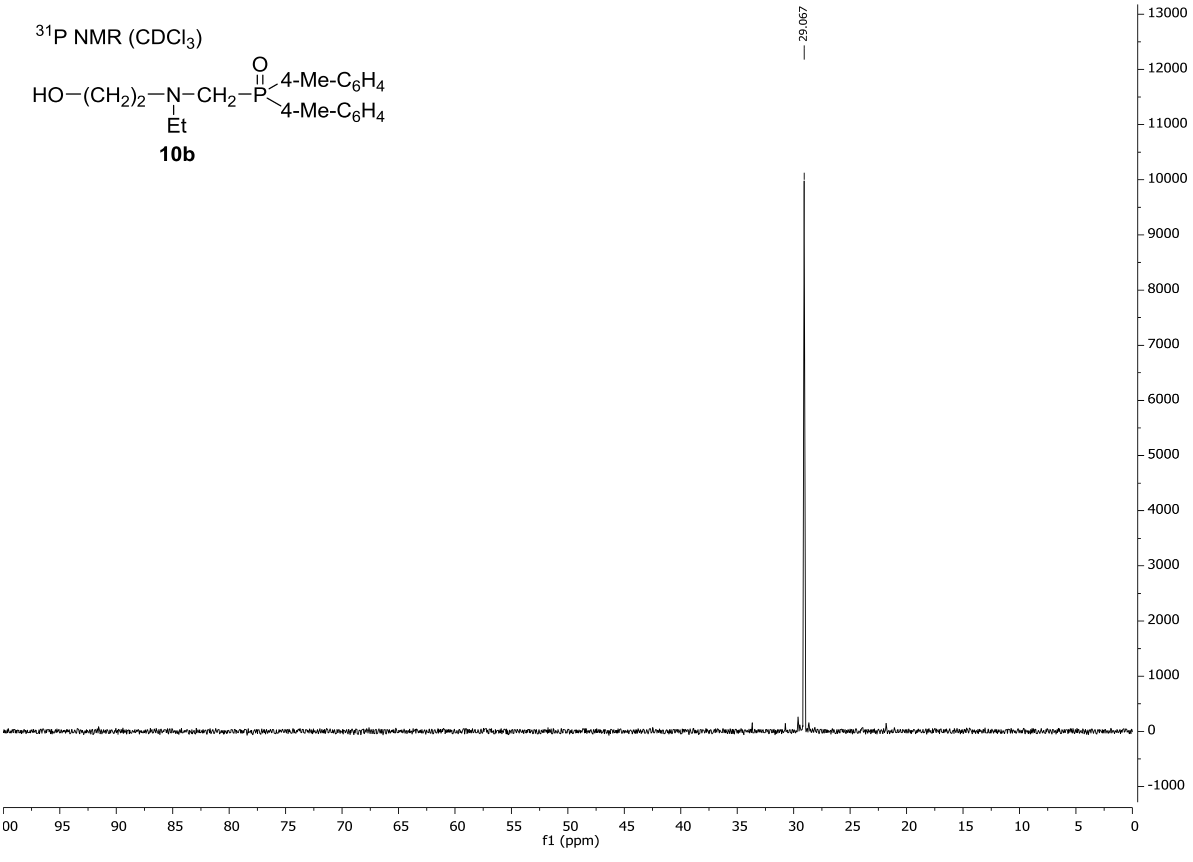

31P NMR (CDCl3)
HO−(CH2)2−N−CH2−P(O)(4-Me-C6H4)2
Et
10b
29.067
f1 (ppm)

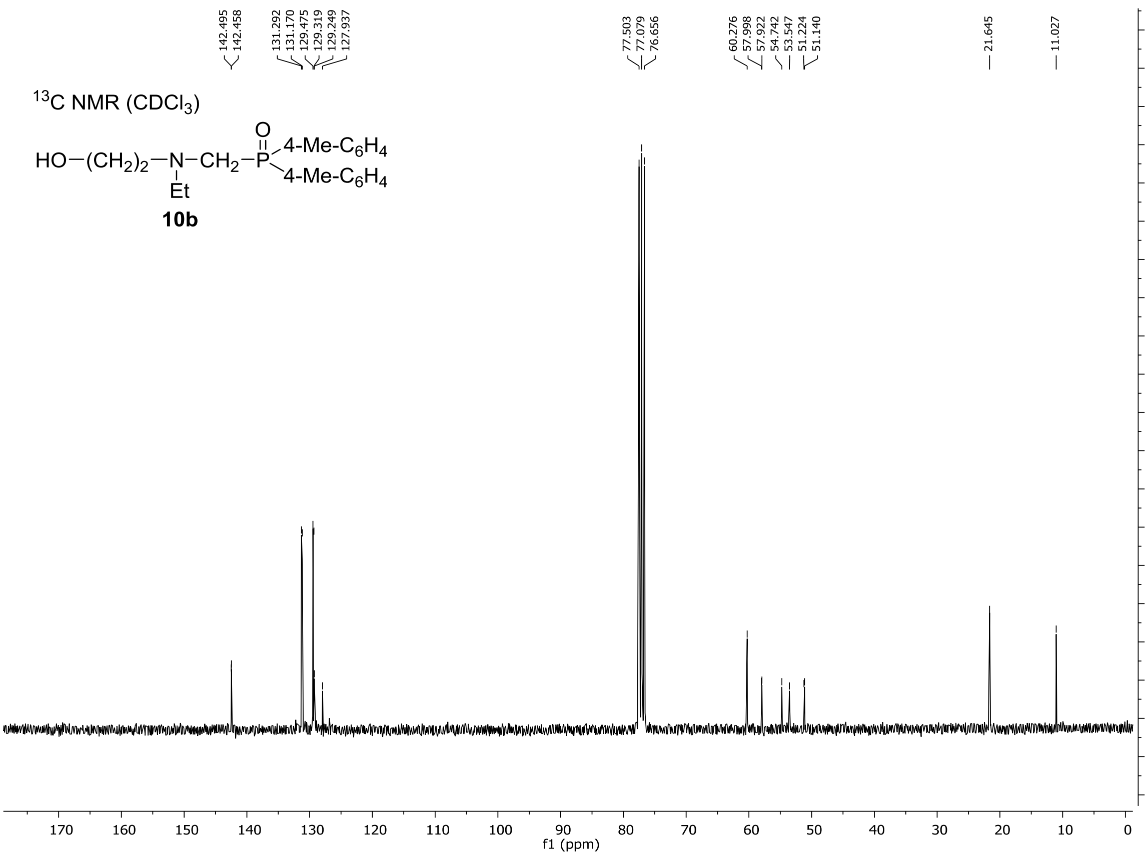

142.495
142.458
131.292
131.170
129.475
129.319
129.249
127.937
77.503
77.079
76.656
60.276
57.998
57.922
54.742
53.547
51.224
51.140
21.645
11.027
^{13}C NMR ($CDCl_3$)
HO−(CH_2)$_2$−N−CH_2−P(O)(4-Me-C_6H_4)$_2$
Et
10b
f1 (ppm)

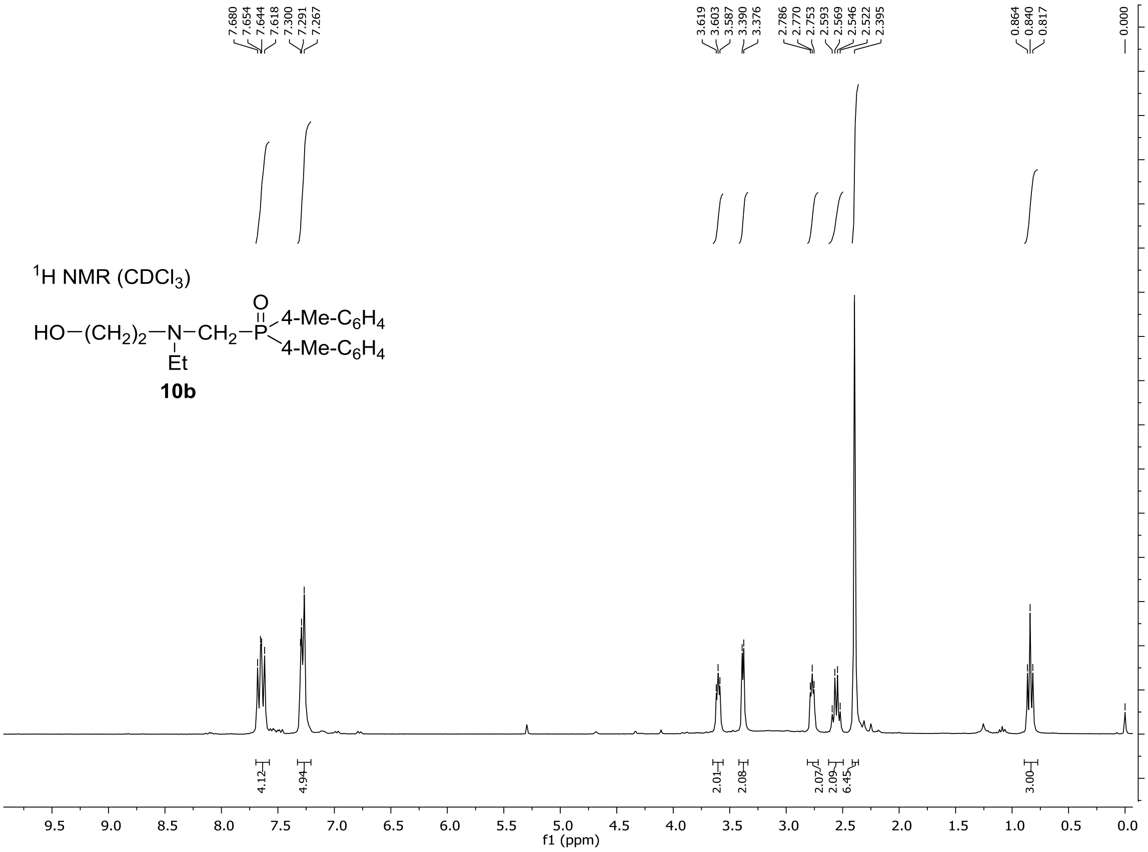

7.680
7.654
7.644
7.618
7.300
7.291
7.267
3.619
3.603
3.587
3.390
3.376
2.786
2.770
2.753
2.593
2.569
2.546
2.522
2.395
0.864
0.840
0.817
0.000
^{1}H NMR ($CDCl_3$)
HO−(CH_2)$_2$−N−CH_2−P(O)(4-Me-C_6H_4)$_2$
Et
10b
4.12
4.94
2.01
2.08
2.07
2.09
6.45
3.00
f1 (ppm)

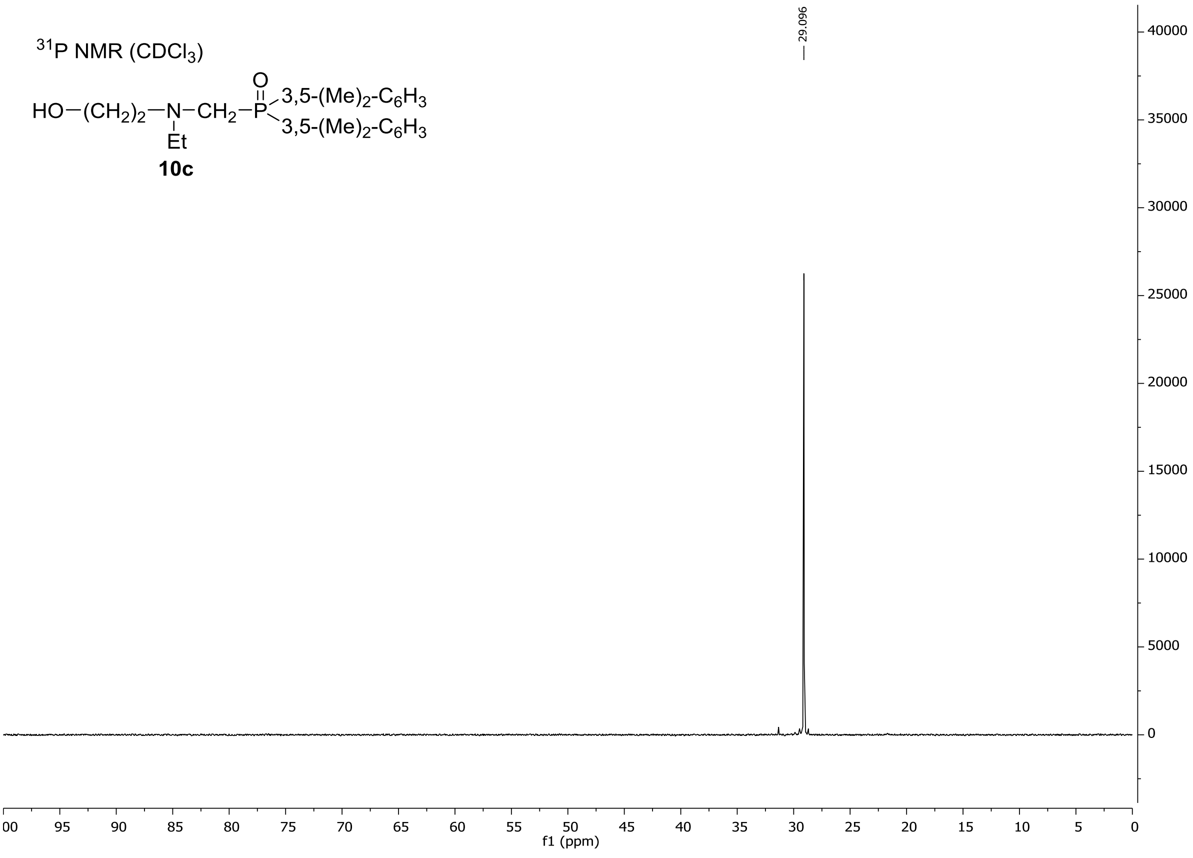

29.096
^{31}P NMR (CDCl$_3$)
HO−(CH$_2$)$_2$−N−CH$_2$−P(=O)(3,5-(Me)$_2$-C$_6$H$_3$)$_2$
Et
10c
f1 (ppm)

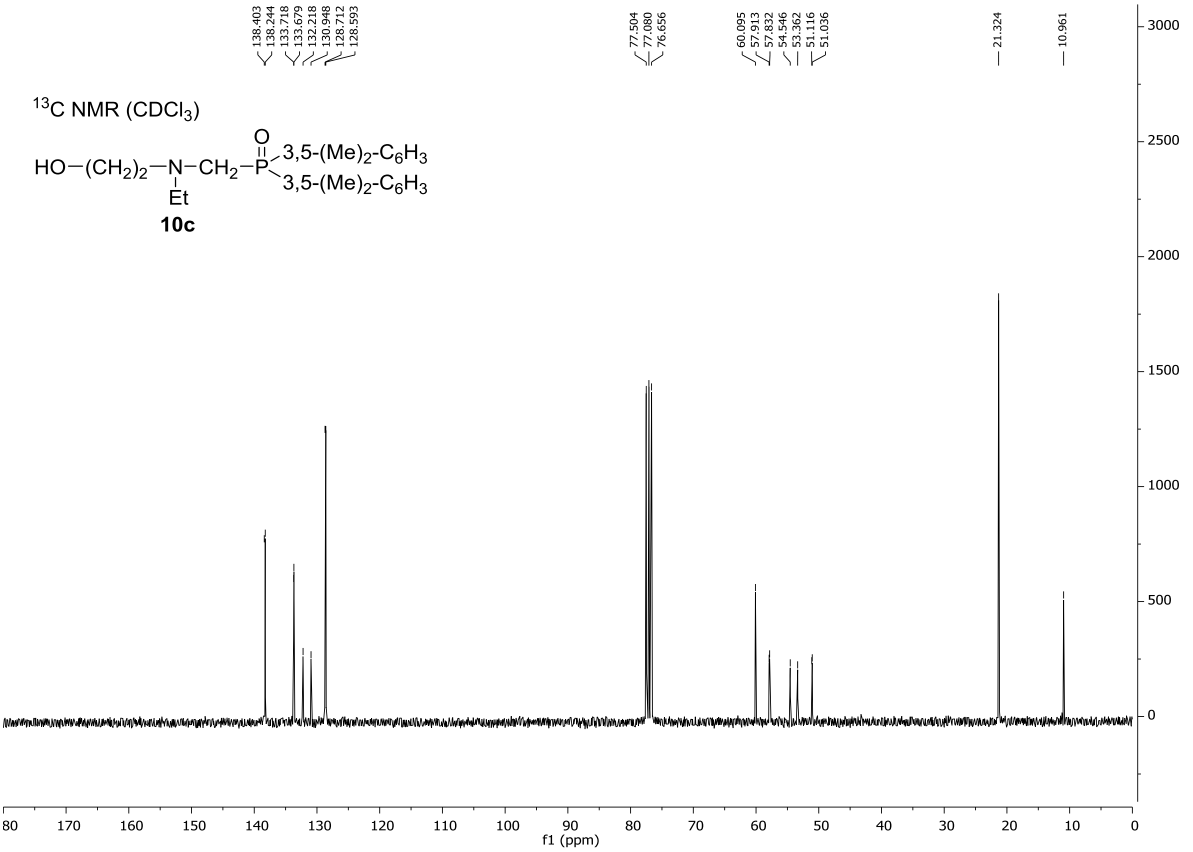

138.403 138.244 133.718 133.679 132.218 130.948 128.712 128.593
77.504 77.080 76.656
60.095 57.913 57.832 54.546 53.362 51.116 51.036
21.324
10.961
^{13}C NMR (CDCl$_3$)
HO−(CH$_2$)$_2$−N−CH$_2$−P(=O)(3,5-(Me)$_2$-C$_6$H$_3$)$_2$
Et
10c
f1 (ppm)

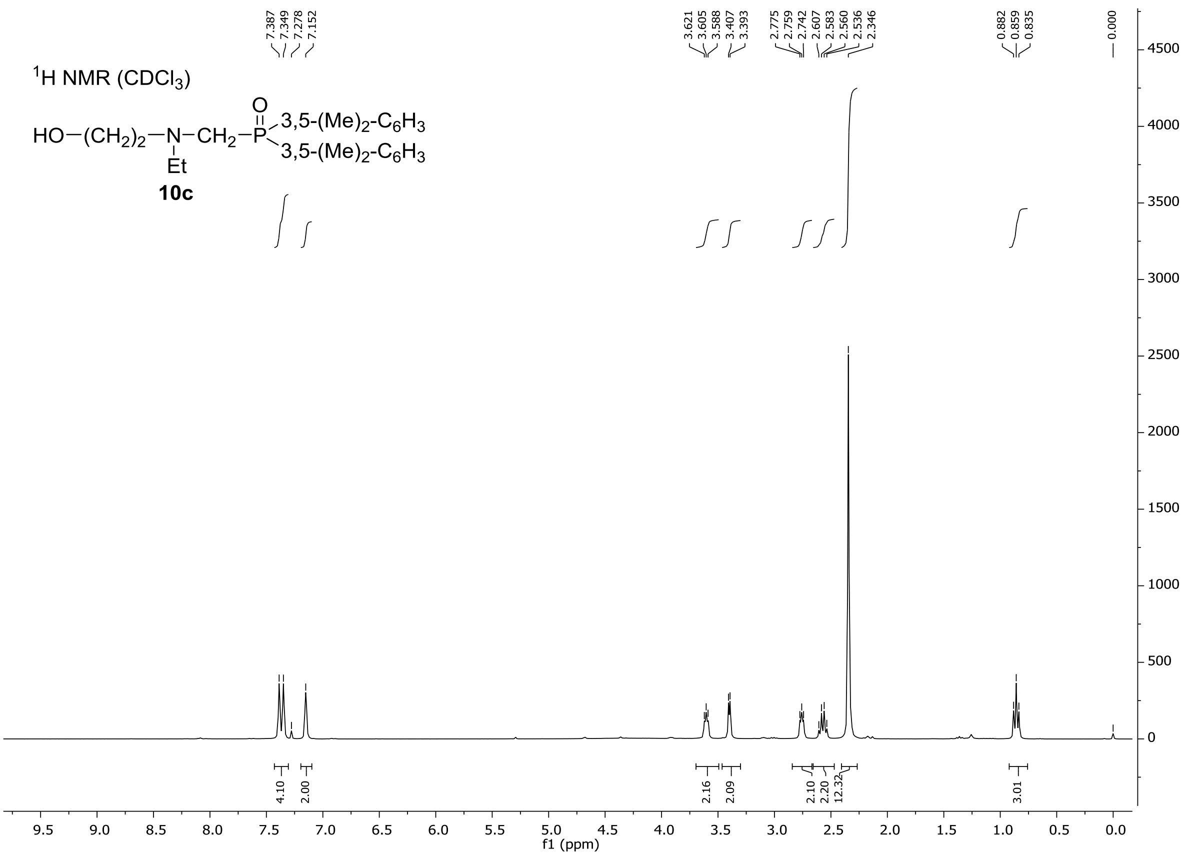
^{1}H NMR ($CDCl_3$)
HO−(CH2)2−N−CH2−P(O)(3,5-(Me)2-C6H3)2
Et
10c
7.387 7.349 7.278 7.152
3.621 3.605 3.588 3.407 3.393
2.775 2.759 2.742 2.607 2.583 2.560 2.536 2.346
0.882 0.859 0.835
0.000
4.10 2.00 2.16 2.09 2.10 2.20 12.32 3.01
f1 (ppm)

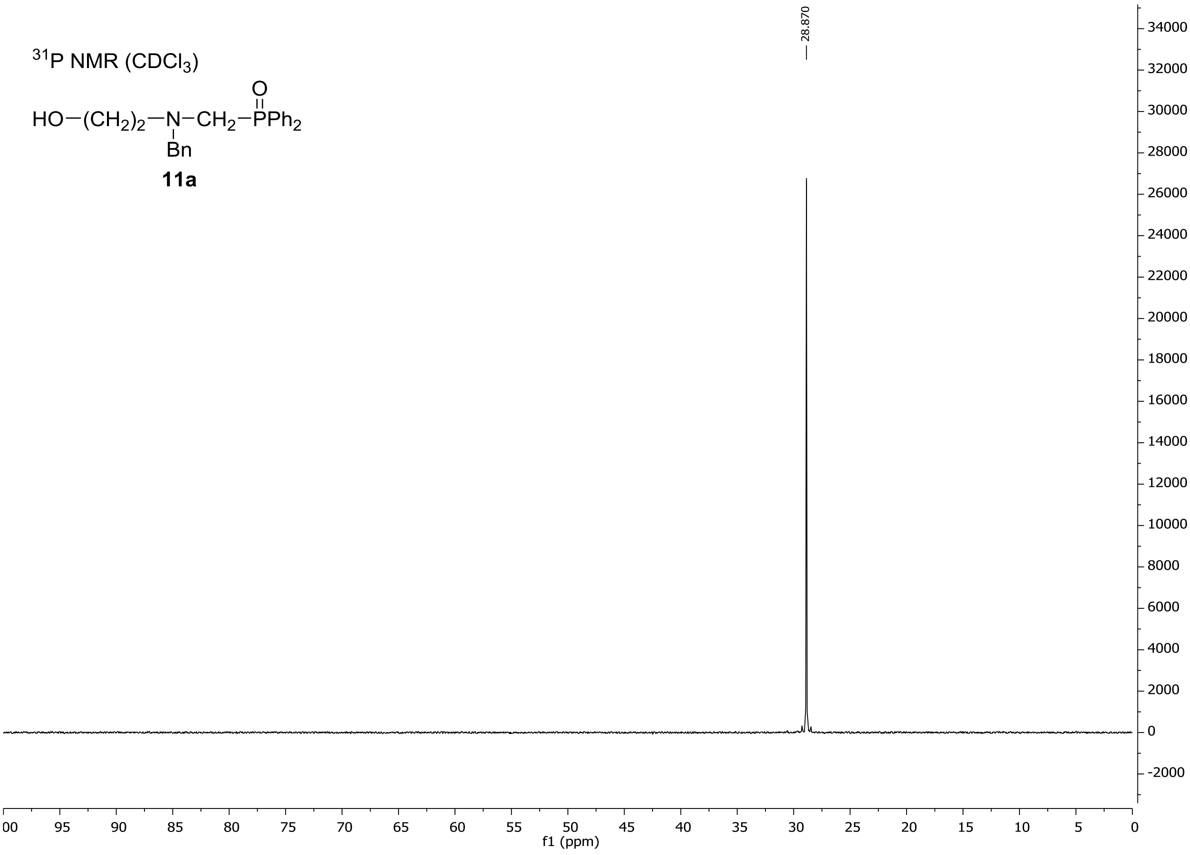
^{31}P NMR ($CDCl_3$)
HO−(CH2)2−N−CH2−P(O)Ph2
Bn
11a
28.870
f1 (ppm)

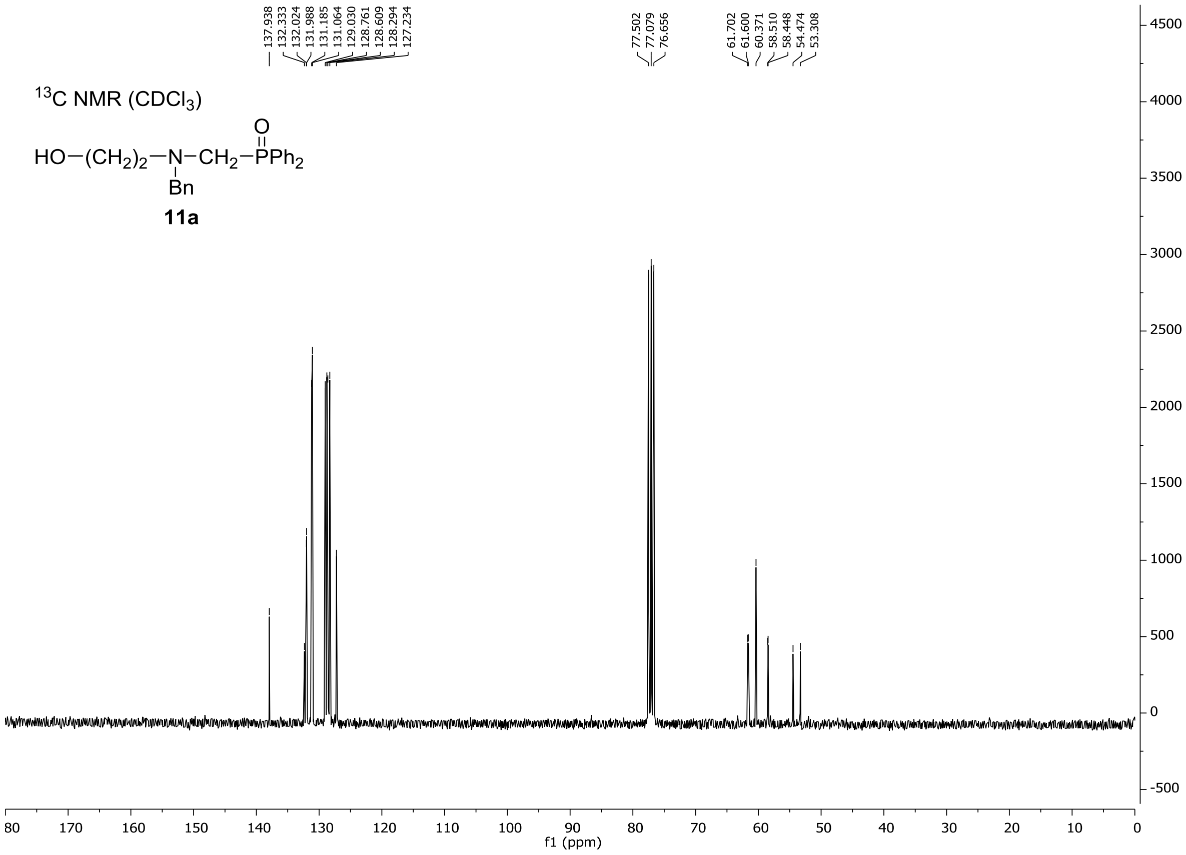
137.938
132.333
132.024
131.988
131.185
131.064
129.030
128.761
128.609
128.294
127.234
77.502
77.079
76.656
61.702
61.600
60.371
58.510
58.448
54.474
53.308
^{13}C NMR (CDCl$_3$)
HO−(CH$_2$)$_2$−N−CH$_2$−P(O)Ph$_2$
Bn
11a
f1 (ppm)

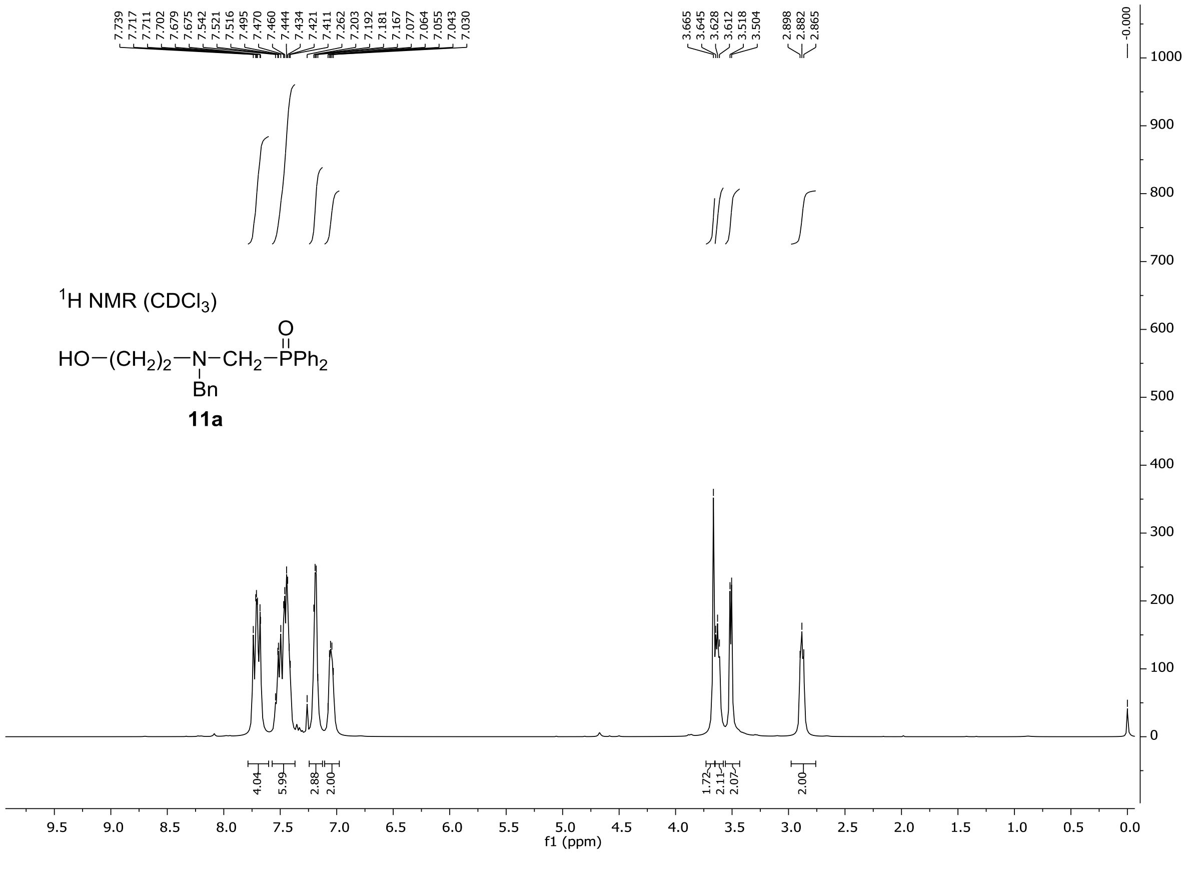
7.739
7.717
7.711
7.702
7.679
7.675
7.542
7.521
7.516
7.495
7.470
7.460
7.444
7.434
7.421
7.411
7.262
7.203
7.192
7.181
7.167
7.077
7.064
7.055
7.043
7.030
3.665
3.645
3.628
3.612
3.518
3.504
2.898
2.882
2.865
-0.000
^{1}H NMR (CDCl$_3$)
HO−(CH$_2$)$_2$−N−CH$_2$−P(O)Ph$_2$
Bn
11a
4.04
5.99
2.88
2.00
1.72
2.11
2.07
2.00
f1 (ppm)

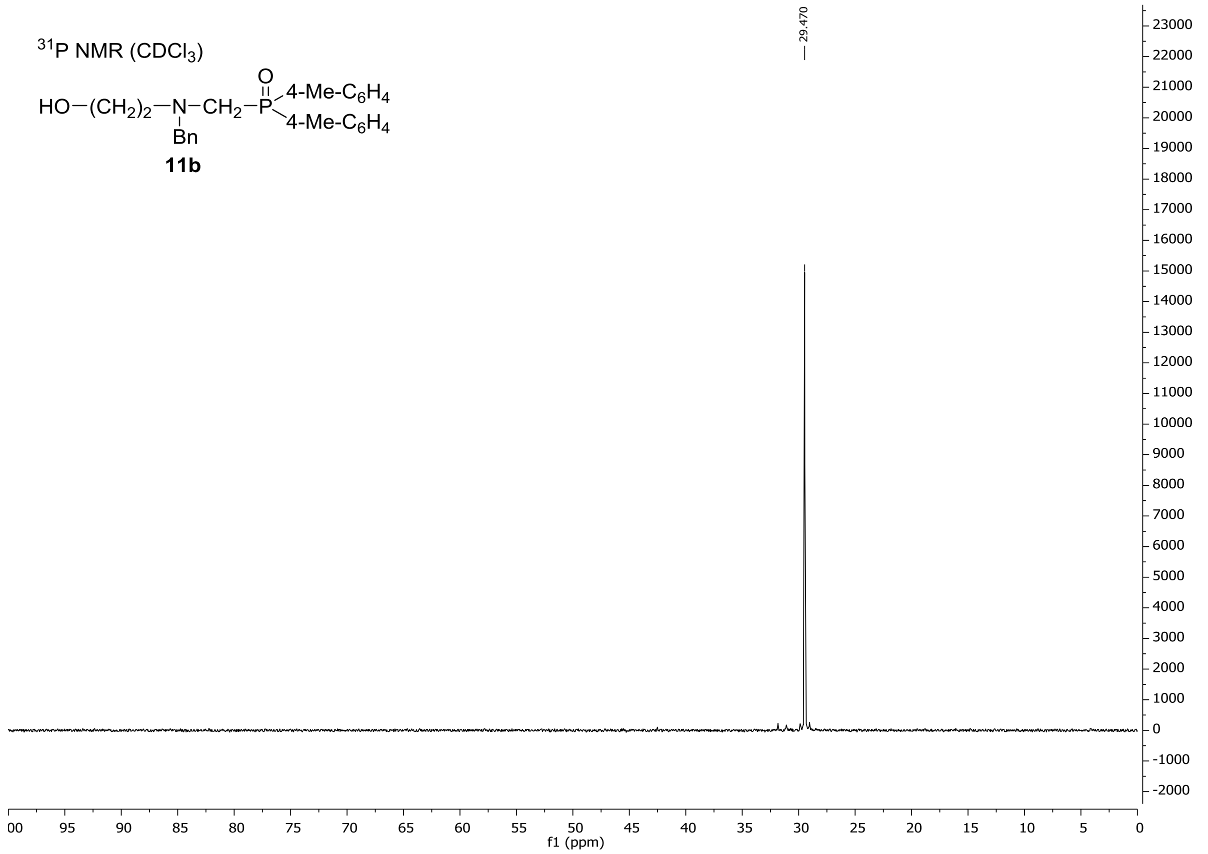
^{31}P NMR (CDCl$_3$)
11b
29.470
f1 (ppm)

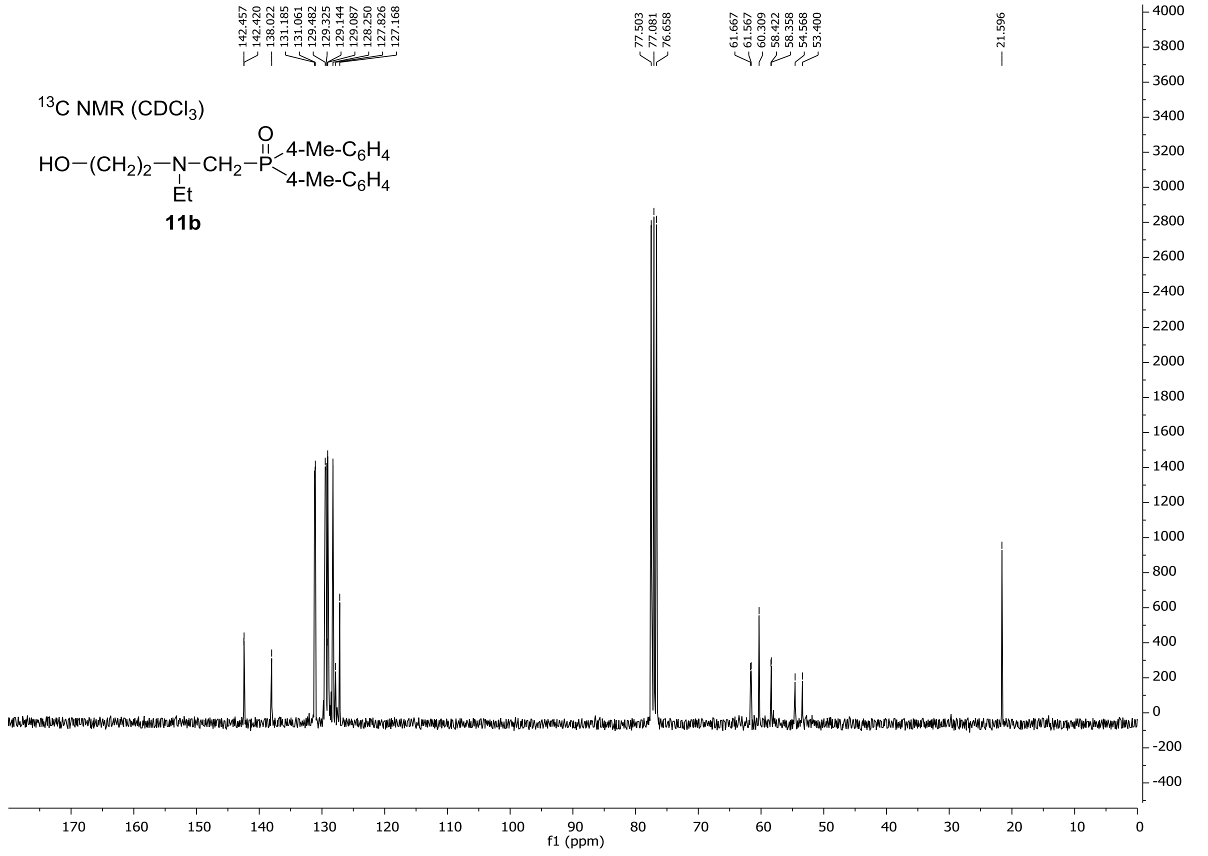
^{13}C NMR (CDCl$_3$)
11b
142.457
142.420
138.022
131.185
131.061
129.482
129.325
129.144
129.087
128.250
127.826
127.168
77.503
77.081
76.658
61.667
61.567
60.309
58.422
58.358
54.568
53.400
21.596
f1 (ppm)

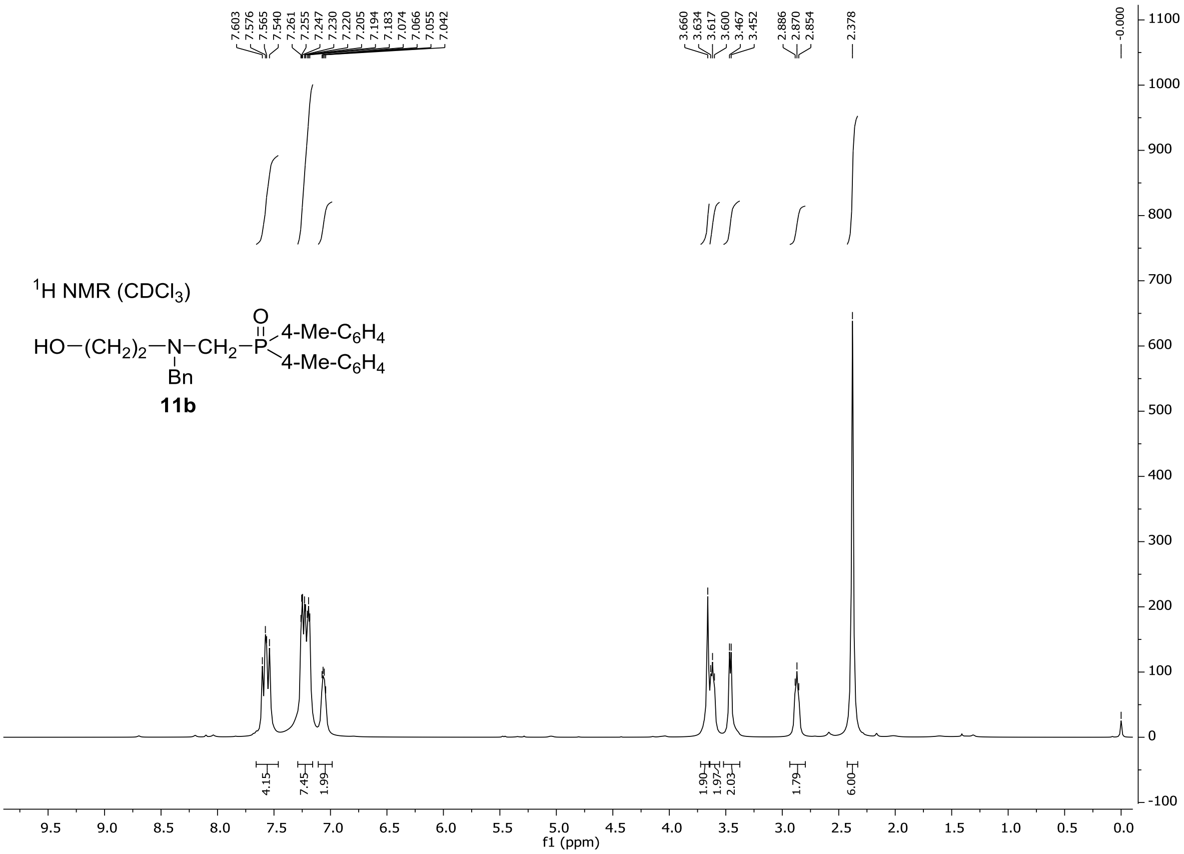

7.603
7.576
7.565
7.540
7.261
7.255
7.247
7.230
7.220
7.205
7.194
7.183
7.074
7.066
7.055
7.042
3.660
3.634
3.617
3.600
3.467
3.452
2.886
2.870
2.854
2.378
-0.000
^{1}H NMR (CDCl$_3$)
HO−(CH$_2$)$_2$−N−CH$_2$−P(O)(4-Me-C$_6$H$_4$)$_2$
Bn
11b
4.15
7.45
1.99
1.90
1.97
2.03
1.79
6.00
f1 (ppm)

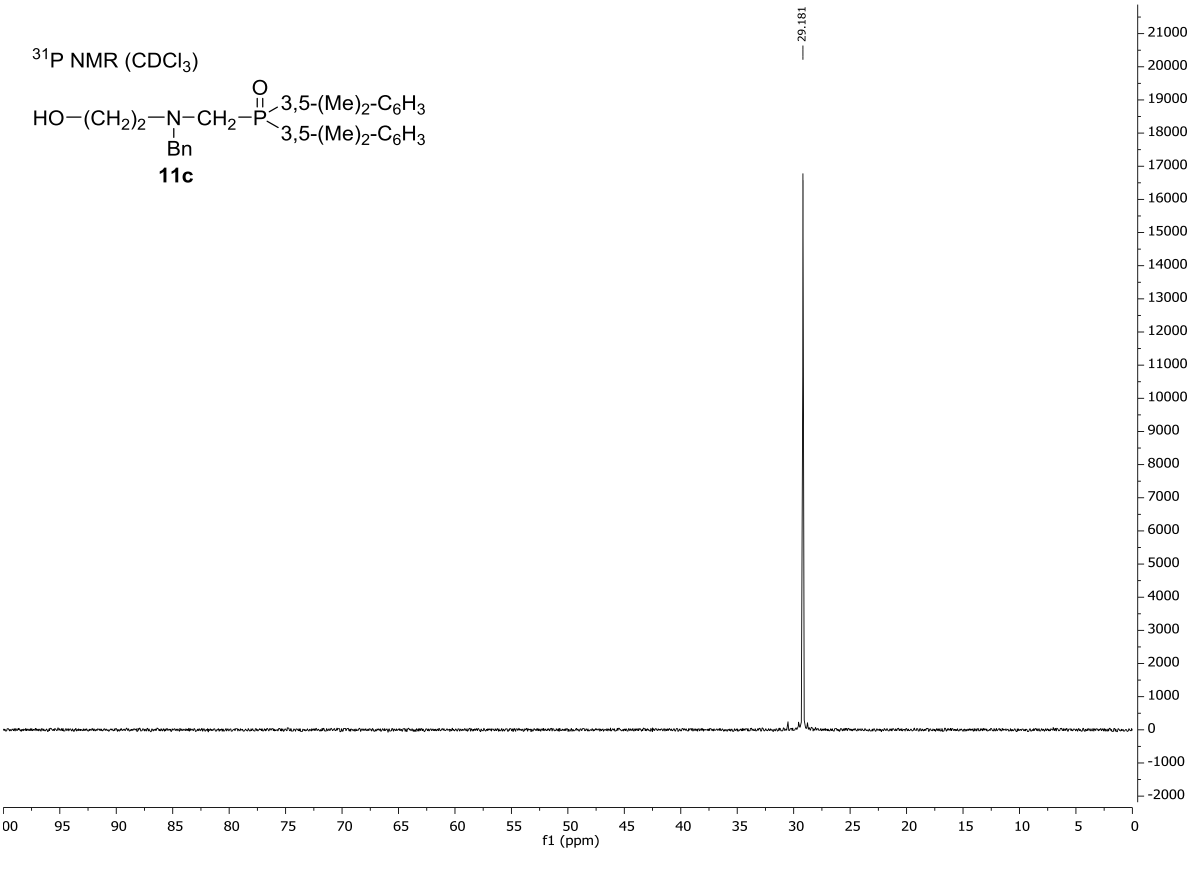

29.181
^{31}P NMR (CDCl$_3$)
HO−(CH$_2$)$_2$−N−CH$_2$−P(O)(3,5-(Me)$_2$-C$_6$H$_3$)$_2$
Bn
11c
f1 (ppm)

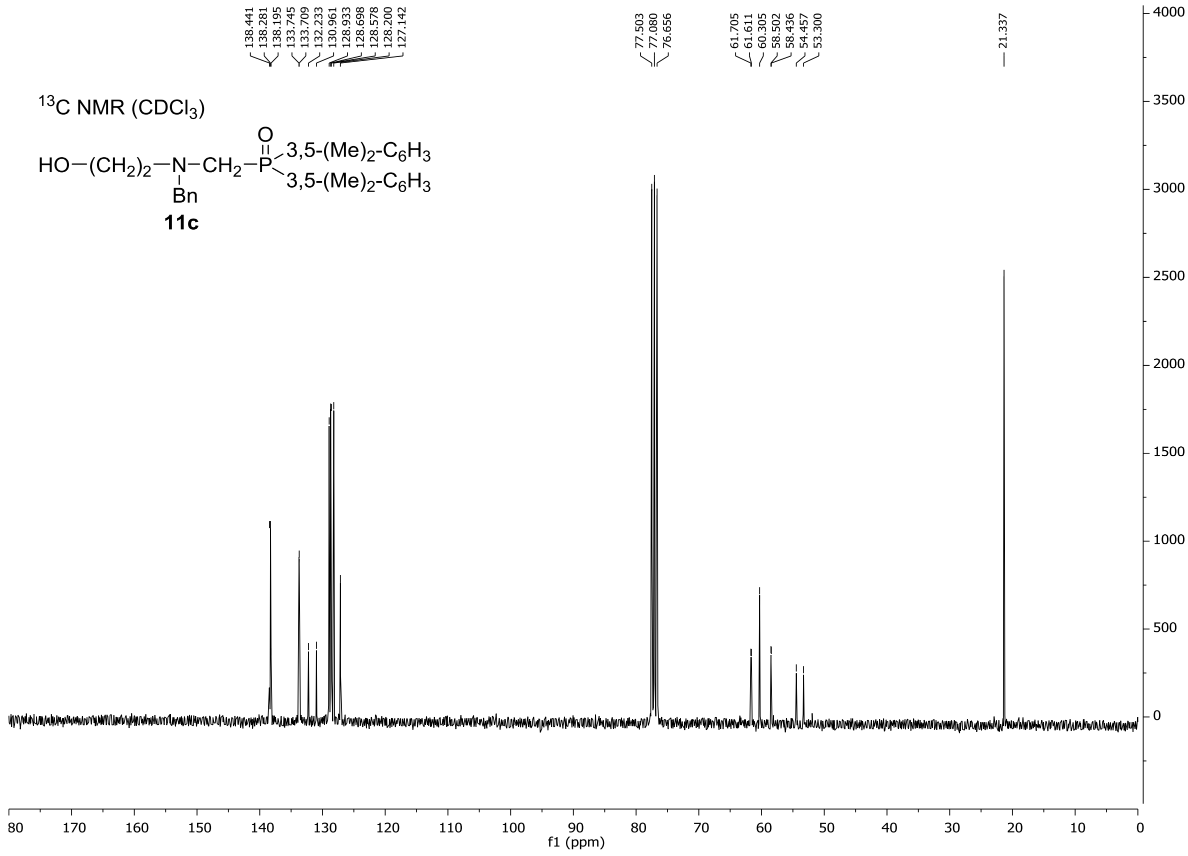
138.441
138.281
138.195
133.745
133.709
132.233
130.961
128.933
128.698
128.578
128.200
127.142
77.503
77.080
76.656
61.705
61.611
60.305
58.502
58.436
54.457
53.300
21.337
13C NMR (CDCl3)
HO−(CH2)2−N−CH2−P(O) 3,5-(Me)2-C6H3
3,5-(Me)2-C6H3
Bn
11c
f1 (ppm)

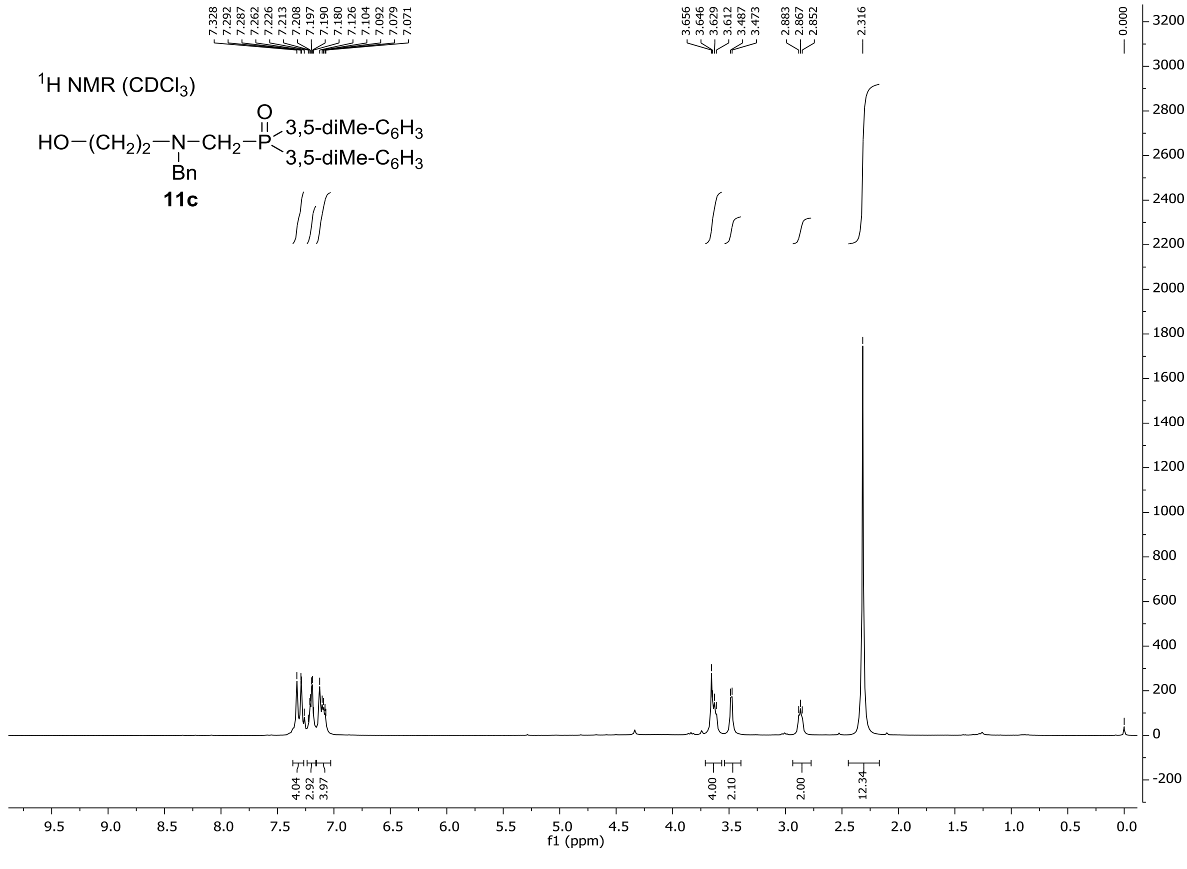
7.328
7.292
7.287
7.262
7.226
7.213
7.208
7.197
7.190
7.180
7.126
7.104
7.092
7.079
7.071
3.656
3.646
3.629
3.612
3.487
3.473
2.883
2.867
2.852
2.316
0.000
1H NMR (CDCl3)
HO−(CH2)2−N−CH2−P(O) 3,5-diMe-C6H3
3,5-diMe-C6H3
Bn
11c
4.04
2.92
3.97
4.00
2.10
2.00
12.34
f1 (ppm)

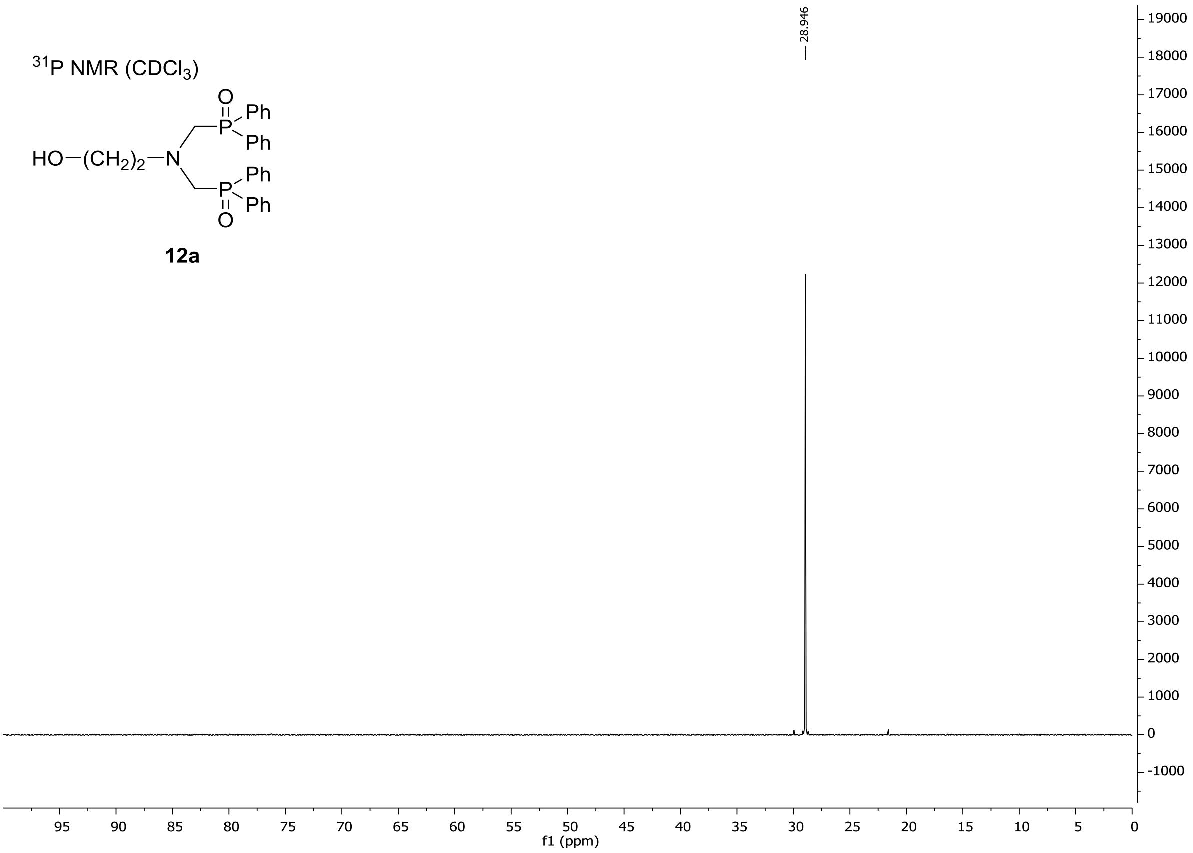

^{31}P NMR (CDCl$_3$)
12a
28.946
f1 (ppm)

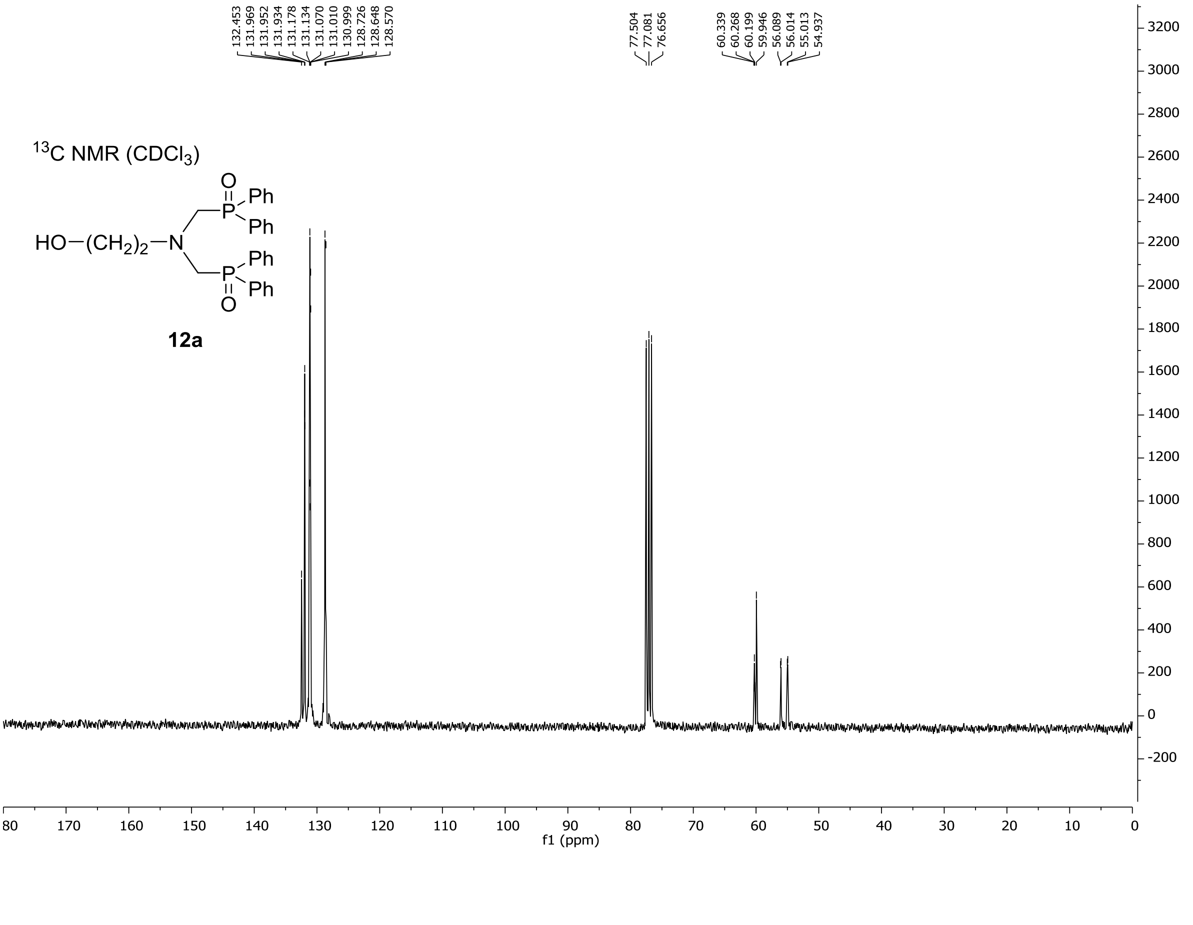

^{13}C NMR (CDCl$_3$)
12a
132.453
131.969
131.952
131.934
131.178
131.134
131.070
131.010
130.999
128.726
128.648
128.570
77.504
77.081
76.656
60.339
60.268
60.199
59.946
56.089
56.014
55.013
54.937
f1 (ppm)

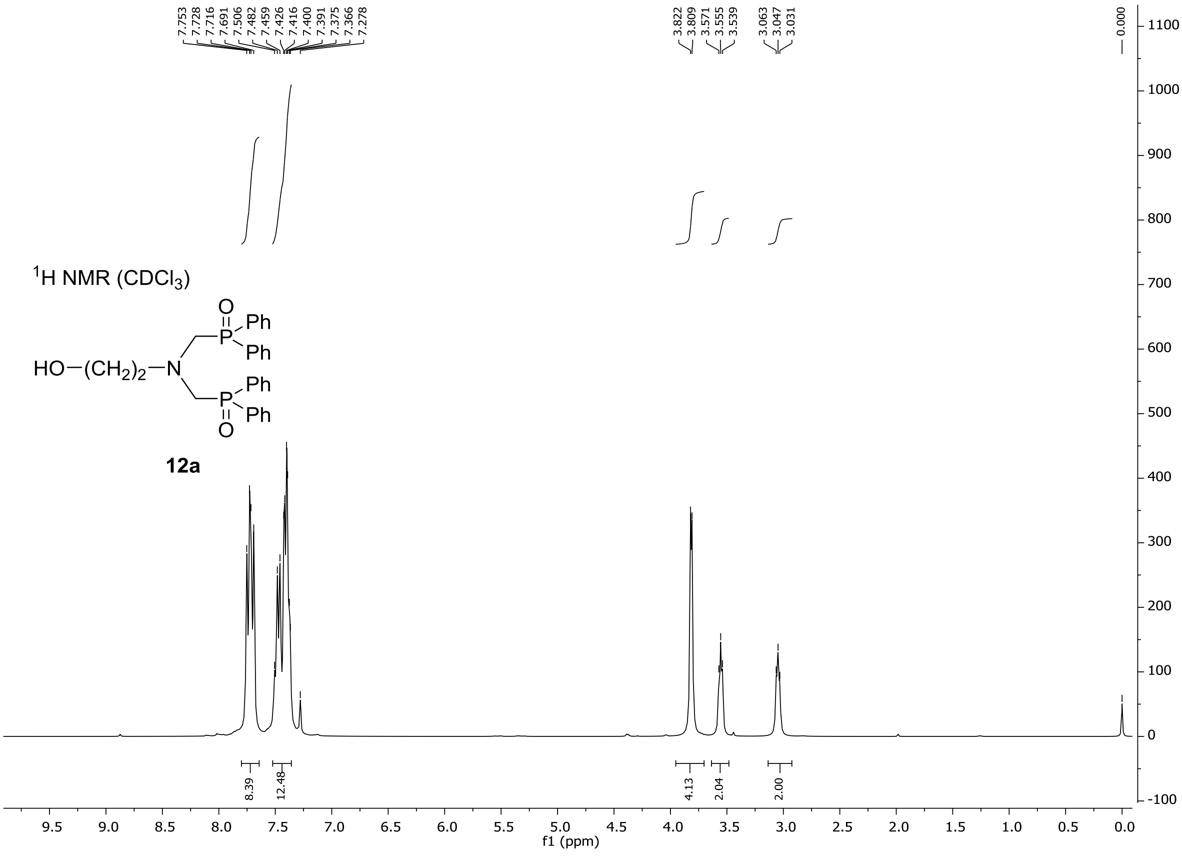

1H NMR (CDCl3)
HO−(CH2)2−N
P
O
Ph
Ph
12a
7.753
7.728
7.716
7.691
7.506
7.482
7.459
7.426
7.416
7.400
7.391
7.375
7.366
7.278
3.822
3.809
3.571
3.555
3.539
3.063
3.047
3.031
0.000
8.39
12.48
4.13
2.04
2.00
f1 (ppm)

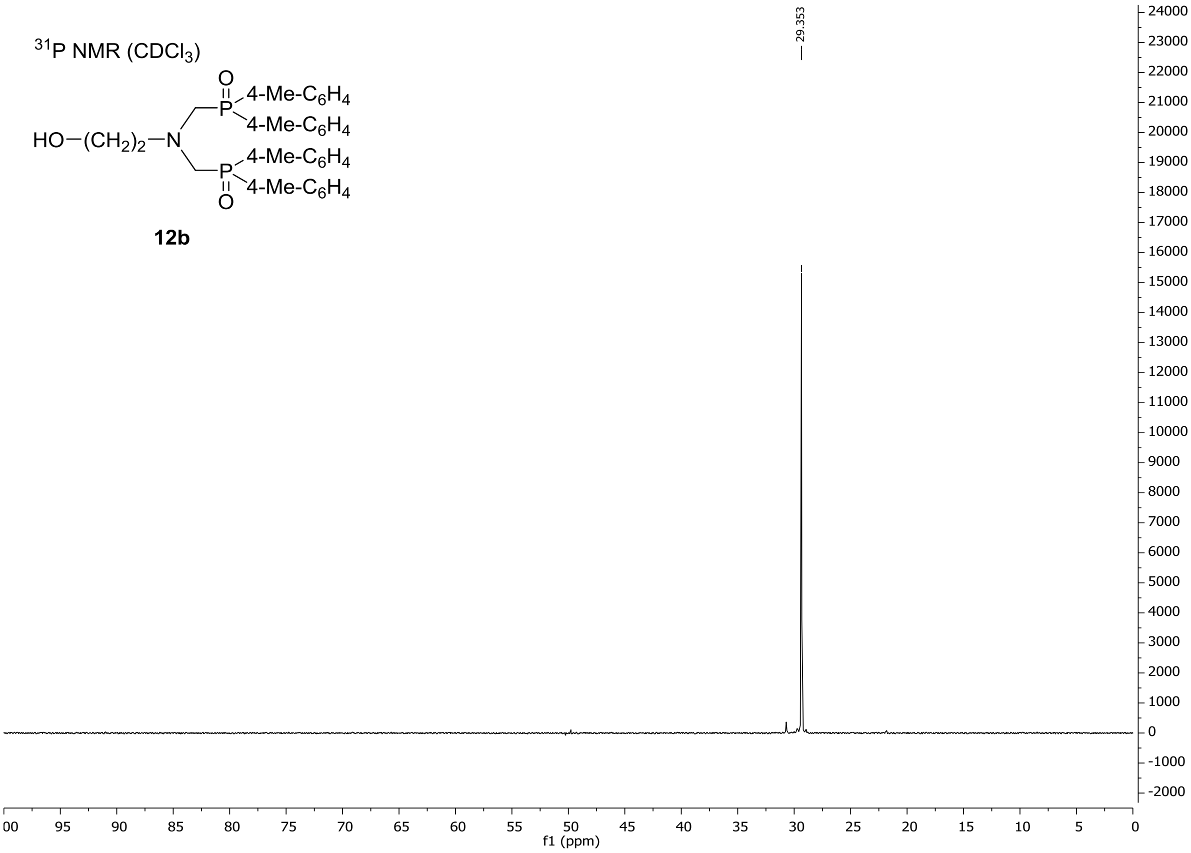

31P NMR (CDCl3)
HO−(CH2)2−N
P
O
4-Me-C6H4
12b
29.353
f1 (ppm)

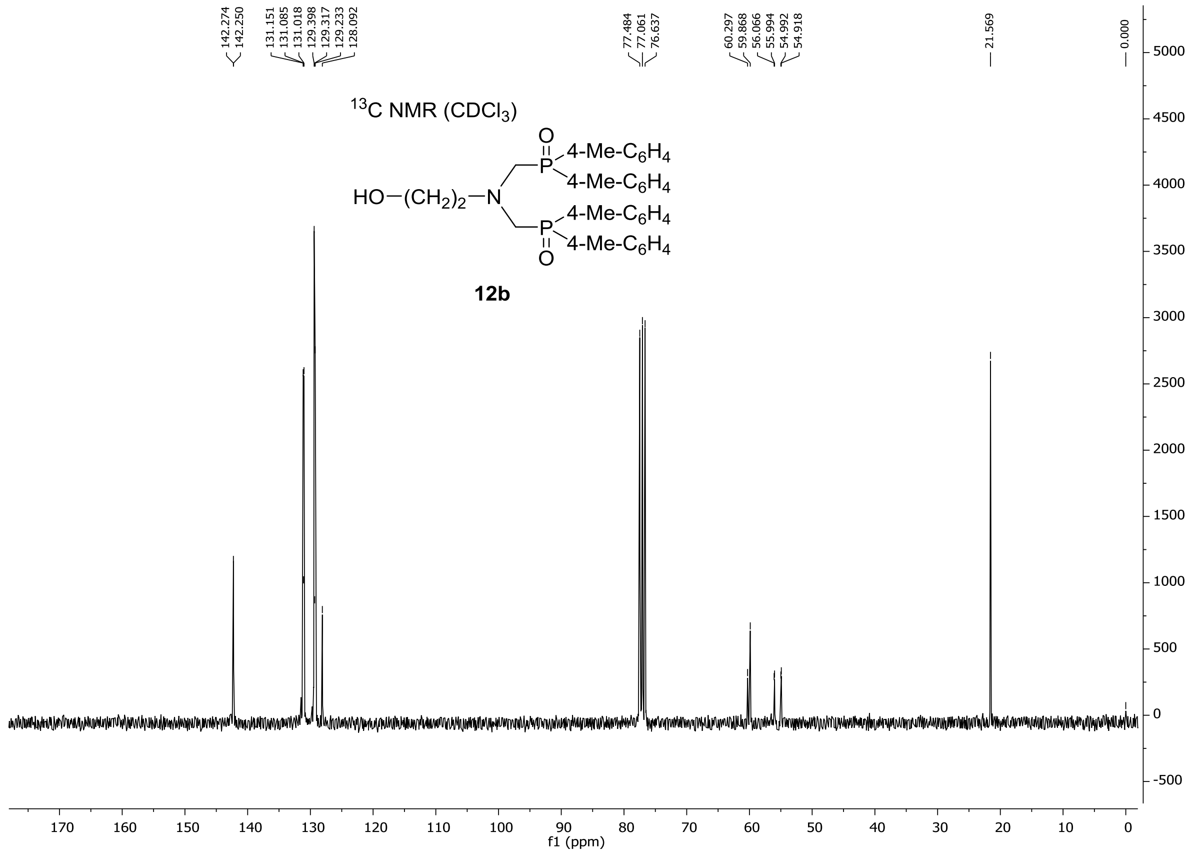

142.274
142.250
131.151
131.085
131.018
129.398
129.317
129.233
128.092
77.484
77.061
76.637
60.297
59.868
56.066
55.994
54.992
54.918
21.569
0.000
^{13}C NMR (CDCl$_3$)
HO−(CH$_2$)$_2$−N
4-Me-C$_6$H$_4$
12b
f1 (ppm)

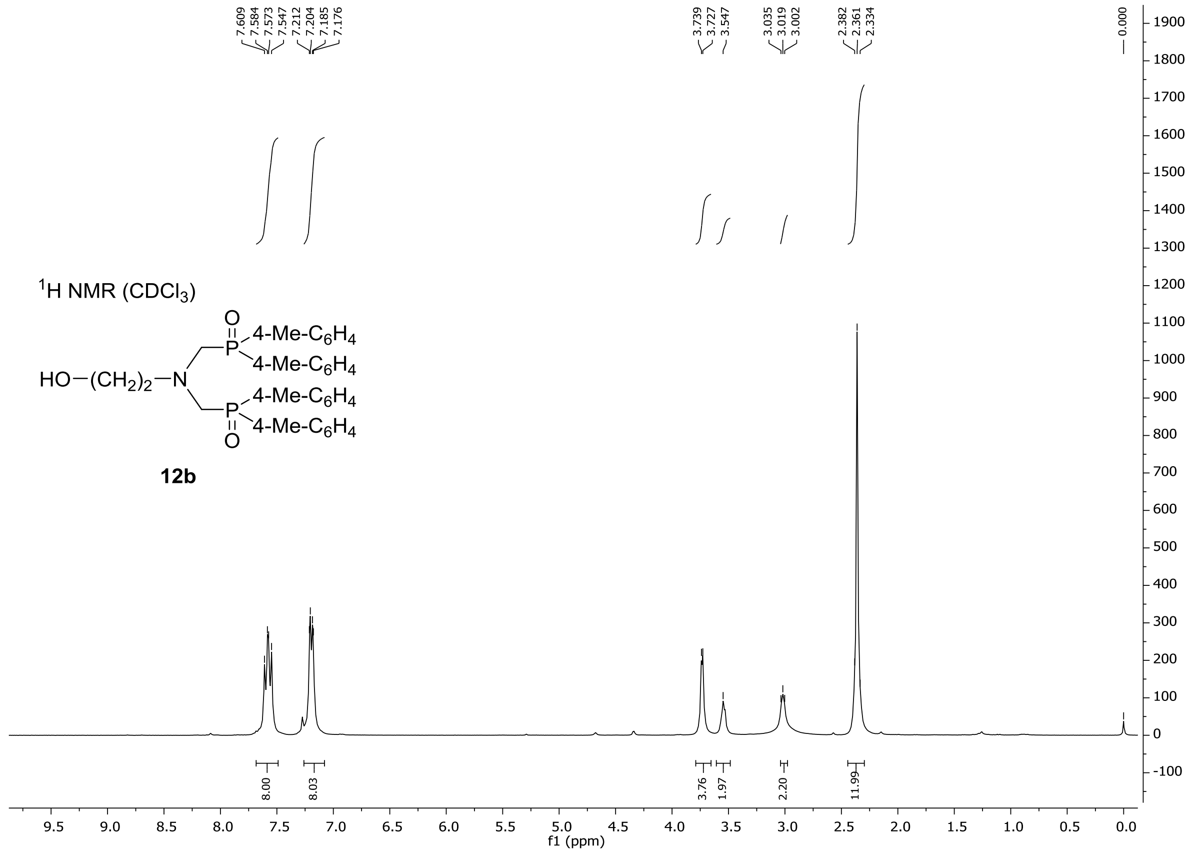

7.609
7.584
7.573
7.547
7.212
7.204
7.185
7.176
3.739
3.727
3.547
3.035
3.019
3.002
2.382
2.361
2.334
0.000
^{1}H NMR (CDCl$_3$)
HO−(CH$_2$)$_2$−N
4-Me-C$_6$H$_4$
12b
8.00
8.03
3.76
1.97
2.20
11.99
f1 (ppm)

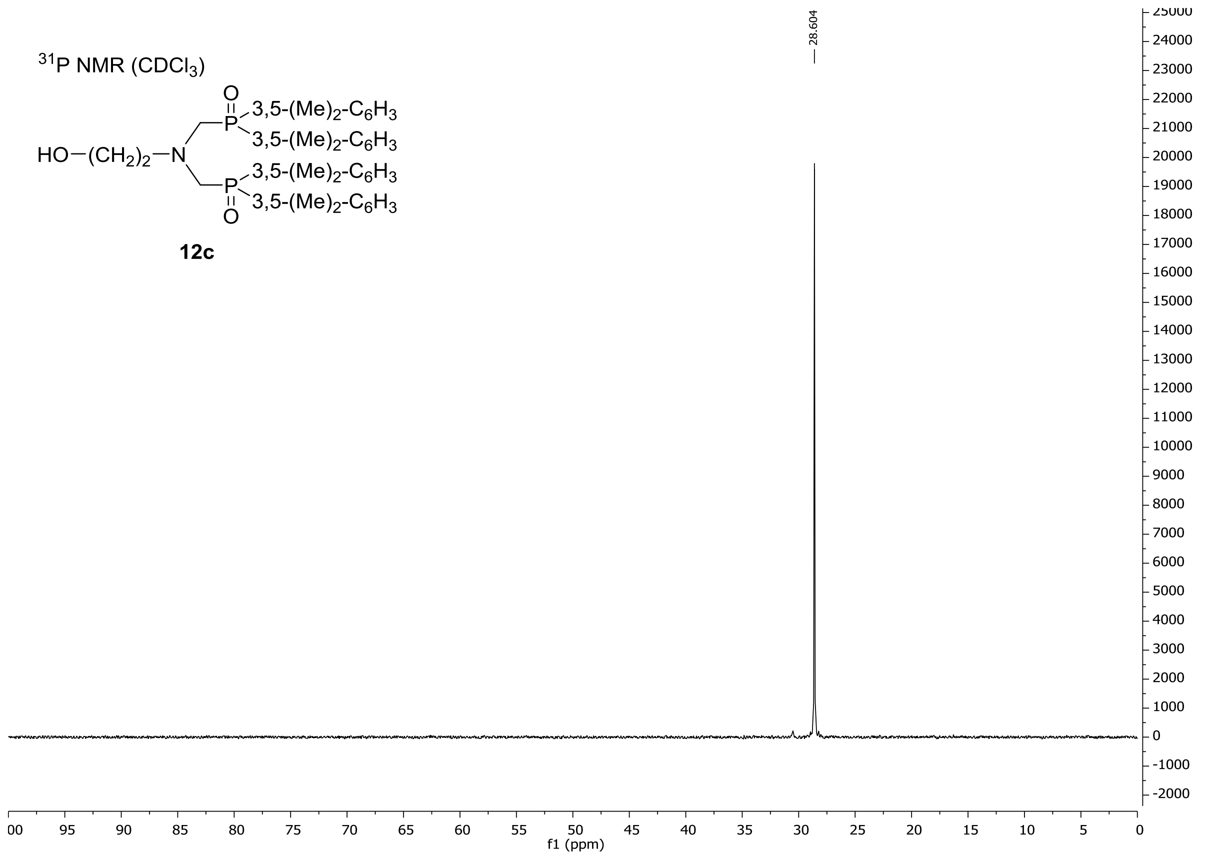
31P NMR (CDCl3)
3,5-(Me)2-C6H3
3,5-(Me)2-C6H3
HO−(CH2)2−N
3,5-(Me)2-C6H3
3,5-(Me)2-C6H3
12c
28.604
f1 (ppm)

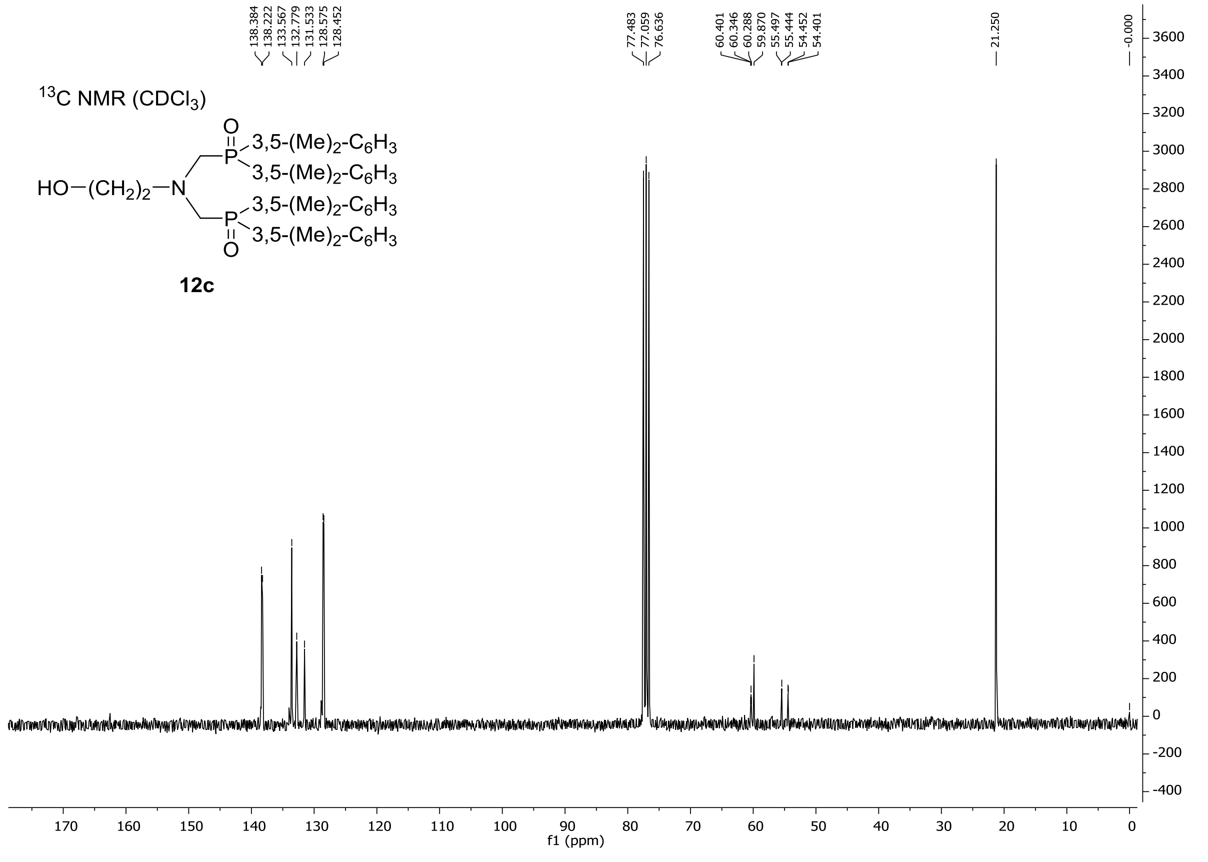
13C NMR (CDCl3)
3,5-(Me)2-C6H3
3,5-(Me)2-C6H3
HO−(CH2)2−N
3,5-(Me)2-C6H3
3,5-(Me)2-C6H3
12c
138.384
138.222
133.567
132.779
131.533
128.575
128.452
77.483
77.059
76.636
60.401
60.346
60.288
59.870
55.497
55.444
54.452
54.401
21.250
-0.000
f1 (ppm)

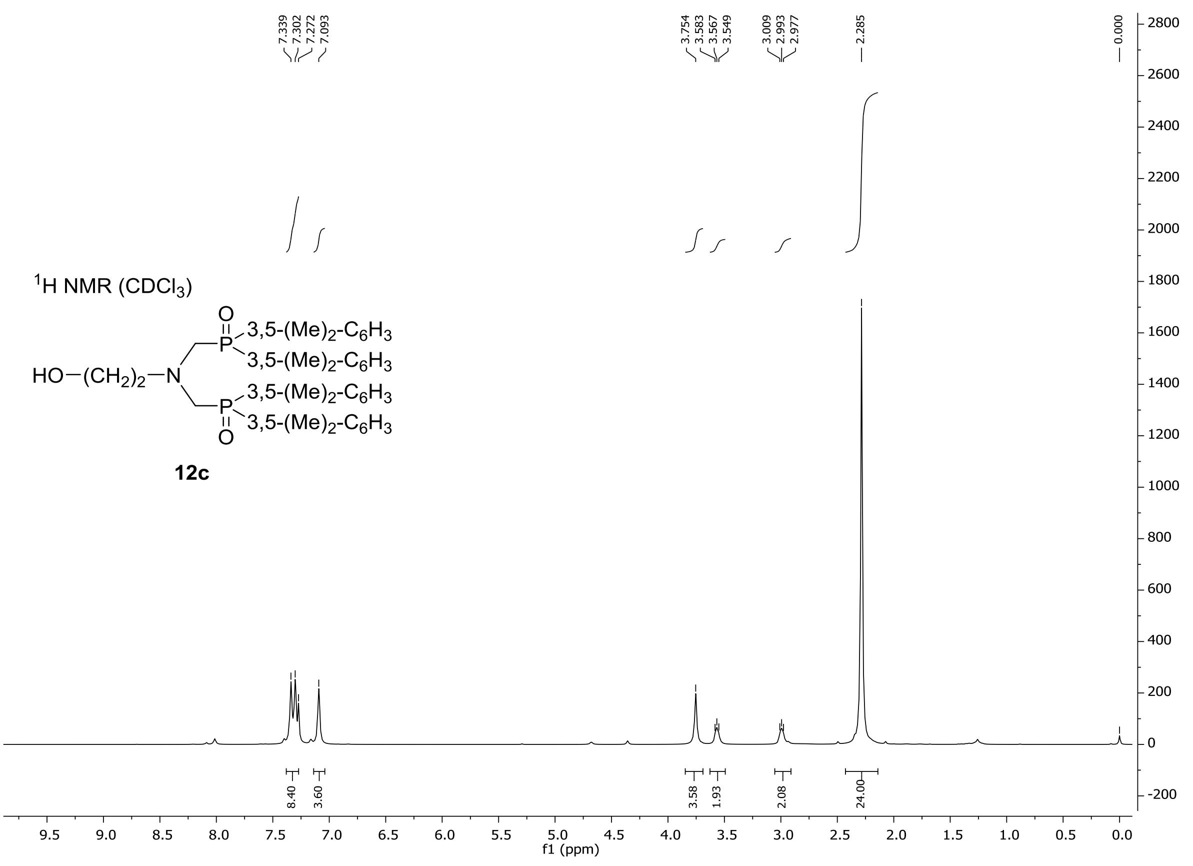

1H NMR (CDCl3)
HO—(CH2)2—N
3,5-(Me)2-C6H3
12c
7.339
7.302
7.272
7.093
3.754
3.583
3.567
3.549
3.009
2.993
2.977
2.285
0.000
8.40
3.60
3.58
1.93
2.08
24.00
f1 (ppm)
